# ATM orchestrates the DNA-damage response to counter toxic non-homologous end-joining at broken replication forks

**DOI:** 10.1038/s41467-018-07729-2

**Published:** 2019-01-08

**Authors:** Gabriel Balmus, Domenic Pilger, Julia Coates, Mukerrem Demir, Matylda Sczaniecka-Clift, Ana C. Barros, Michael Woods, Beiyuan Fu, Fengtang Yang, Elisabeth Chen, Matthias Ostermaier, Tatjana Stankovic, Hannes Ponstingl, Mareike Herzog, Kosuke Yusa, Francisco Munoz Martinez, Stephen T. Durant, Yaron Galanty, Petra Beli, David J. Adams, Allan Bradley, Emmanouil Metzakopian, Josep V. Forment, Stephen P. Jackson

**Affiliations:** 1 The Wellcome Trust and Cancer Research UK Gurdon Institute and Department of Biochemistry, University of Cambridge, Tennis Court Road, Cambridge, CB2 1QN UK; 2 Wellcome Trust Sanger Institute, Hinxton, Cambridge CB10 1SA UK; 3 UK Dementia Research Institute and Department of Clinical Neurosciences, University of Cambridge, Cambridge, CB2 0AH UK; 4 Institute of Molecular Biology (IMB), 55128 Mainz, Germany; 5 Institute of Cancer and Genomic Sciences, College of Medical and Dental Sciences, University of Birmingham, Edgbaston, Birmingham B15 2TT UK; 6 DNA Damage Response Biology, Bioscience Oncology IMED Biotech Unit, AstraZeneca, Cambridge, CB4 0WG UK

**Keywords:** Cancer, Cell biology, Genetics, Cancer therapeutic resistance

## Abstract

Mutations in the *ATM* tumor suppressor gene confer hypersensitivity to DNA-damaging chemotherapeutic agents. To explore genetic resistance mechanisms, we performed genome-wide CRISPR-Cas9 screens in cells treated with the DNA topoisomerase I inhibitor topotecan. Thus, we here establish that inactivating terminal components of the non-homologous end-joining (NHEJ) machinery or of the BRCA1-A complex specifically confer topotecan resistance to ATM-deficient cells. We show that hypersensitivity of *ATM*-mutant cells to topotecan or the poly-(ADP-ribose) polymerase (PARP) inhibitor olaparib reflects delayed engagement of homologous recombination at DNA-replication-fork associated single-ended double-strand breaks (DSBs), allowing some to be subject to toxic NHEJ. Preventing DSB ligation by NHEJ, or enhancing homologous recombination by BRCA1-A complex disruption, suppresses this toxicity, highlighting a crucial role for ATM in preventing toxic LIG4-mediated chromosome fusions. Notably, suppressor mutations in *ATM*-mutant backgrounds are different to those in *BRCA1*-mutant scenarios, suggesting new opportunities for patient stratification and additional therapeutic vulnerabilities for clinical exploitation.

## Abstract (web summary)

Mutations in the ATM tumor suppressor gene confer hypersensitivity to DNA-damaging chemotherapeutic agents. Here, the authors provide evidence that these hypersensitivities reflect a crucial role for ATM at damaged replication forks being to prevent toxic DNA end-joining leading to chromosome fusions and cell death.

## Introduction

Rapid recognition and accurate repair of DNA damage is crucial for all organisms. Amongst the various types of DNA lesions that can occur, DNA double-strand breaks (DSBs) are generally considered the most toxic, and can be caused either directly by ionizing radiation (IR) or reactive chemicals, or indirectly via the processing of other types of DNA lesions or breakdown of DNA replication forks. DSB accrual causes physical discontinuities in the genome and can generate pathogenic mutations and chromosomal rearrangements (deletions, inversions, duplications, or translocations) that are hallmarks of cancer^1^. Upon detecting DSBs, cells activate the DNA damage response (DDR), a signal-transduction pathway that, among other roles, promotes activation of DNA-damage-dependent checkpoints that slow or halt cell-cycle progression. This allows more time for DNA repair, which in the case of DSBs mainly involves the use of non-homologous end-joining (NHEJ) or homologous recombination (HR) repair (HRR) pathways^2,3^.

NHEJ is employed to rejoin double-ended DSBs that occur when the two strands of the DNA double helix are simultaneously broken in close proximity. Classical NHEJ is initiated by DSB recruitment of the Ku70/80 heterodimer, which constrains the DSB and engages with the DNA-dependent protein kinase catalytic subunit (DNA-PKcs) to form the DNA-PK complex that is subsequently stabilized on damaged chromatin by PAXX. Upon DNA-PK activation, XRCC4, XLF, and DNA ligase IV (LIG4) are recruited to align and ligate the ends independently of sequence homology^4^. On the other hand, HRR involves binding of the MRE11-RAD50-NBS1 (MRN) complex to tether the DSB ends and to recruit and activate the ataxia telangiectasia-mutated (ATM) protein kinase^4^. Together with CTIP, the MRN complex promotes resection of DSB ends to produce single-stranded DNA (ssDNA) overhangs that are protected from forming secondary structures or from nuclease degradation by binding of replication protein A (RPA). RPA is then replaced by the recombinase RAD51, which together with other proteins, mediates strand invasion into the homologous sister chromatid to allow error-free repair^5^. Effective RAD51 loading onto ssDNA requires the actions of the tumor suppressor proteins BRCA1 and BRCA2 as well as other accessory proteins, deficiencies of which cause HRR defects and predisposition to breast, ovarian, and other cancers^6^. Although BRCA1 exists in the cell in several different protein complexes (named BRCA1-A, -B, and -C), their contributions to the different phenotypes of BRCA1-deficient cells are, as yet, poorly understood^7^.

While NHEJ is active throughout interphase, HRR is restricted to the S and G2 phases of the cell-cycle, where a homologous sister chromatid is available as repair template. Several layers of control dictate DSB-repair-pathway choice between NHEJ and HRR, including activation of HR by cyclin-dependent kinase activity^8^, or direct competition between HR- and NHEJ-promoting factors at DSB sites^9^. The latter involves, but is not limited to, regulation of the recruitment kinetics of the MRN and Ku complexes^10^, as well as MRN/CTIP-dependent removal of Ku from DSBs^11^. While it appears that ATM modulates the ability of CTIP to promote Ku removal^11^, it is not yet clear what the impact of losing this function would be in ATM-deficient cells. Additionally, much research interest has been recently focused on the competition between the HRR-promoting factor BRCA1 and the NHEJ-promoting factor 53BP1, although the mechanisms underlying this antagonism are not yet clear^12^. The potential effects of other BRCA1-interacting proteins on DNA end-resection dynamics are not yet well studied either, although defects in components of the BRCA1-A complex (BRCC45, ABRAXAS, MERIT40, RAP80, and BRCC36) have been shown to increase HRR efficiency in a manner that has been linked to enhanced DSB resection^13^.

In addition to being produced by chromosomal rupture, DSBs can also arise when DNA replication forks break down upon encountering DNA lesions such as single-strand breaks, or protein-DNA complexes such as abortive DNA topoisomerase I (TOP1) catalytic intermediates or inhibited/trapped poly(ADP-ribose) polymerase 1 (PARP1) enzyme. In these circumstances, single-ended DSBs (seDSBs) are generated. These structures are not physiologically suited to being acted upon by NHEJ factors, as they would antagonize HRR processes and might produce fusions with other seDSBs to yield chromosomal aberrations. Instead, a specific form of HRR termed break-induced replication (BIR) is employed to restore the replication fork by using the sister chromatid as template, a process that invariably produces sister chromatid exchanges (SCEs)^14,15^. Consequently, drugs producing seDSBs, such as the TOP1 inhibitor (TOP1i) camptothecin (CPT and its derivatives topotecan and irinotecan) or PARP inhibitors such as olaparib, have been shown to be particularly effective in killing cells displaying HRR defects. Indeed, the selective killing of tumor cells carrying mutations in *BRCA1* or *BRCA2* by olaparib has led to the clinical registrations of PARP inhibitors in such settings, and there is hope that this potential will be extended to tumors with mutations in other genes, such as *ATM*, that are thought to share molecular features with *BRCA*-mutant cells^16^.

In this study, we perform genome-wide CRISPR-Cas9 loss-of-function genetic screens to identify suppressors of cell killing by the TOP1i topotecan in ATM-proficient and ATM-deficient cells. We show that, in the absence of ATM, NHEJ-mediated repair of seDSBs induced by TOP1 or PARP1 inhibition results in aberrant chromatid fusions and cell death. Strikingly, both phenotypes can be rescued by impairing NHEJ, either via loss/mutation of LIG4, XLF, or XRCC4, or by promoting increased engagement of HRR via loss of specific components of the BRCA1-A complex. In addition to highlighting potential mechanisms for therapeutic resistance in ATM-deficient cancers, our results suggest that the prime mechanism by which ATM promotes cell survival in response to seDSB generation is not to remove Ku from such structures but to promote efficient DSB resection and thereby prevent seDSB repair by toxic NHEJ.

## Results

### CRISPR screens for suppression of TOP1i sensitivity

**Fig. 1:**
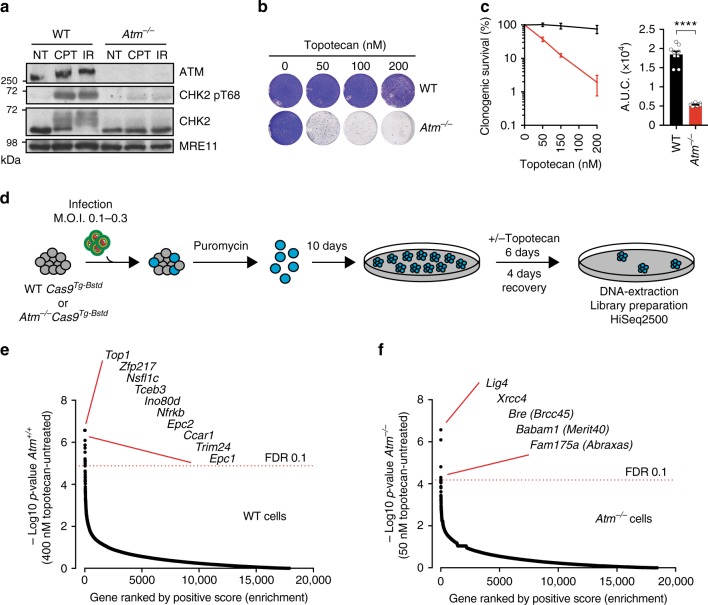
CRISPR-Cas9 screening in WT and ATM-deficient mESCs. **a** Representative immunoblot images show the absence of ATM protein and defective signaling through phosphorylation of its substrate CHK2 on Thr-68. NT untreated, CPT camptothecin (1 μM, 1 h), IR ionizing radiation (10 Gy, 1 h). **b**, **c** Crystal violet cell viability assay (**b**) and clonogenic survival assays (**c**) showing hypersensitivity of ATM-deficient cells to topotecan; *n* = 9/genotype; error bars s.e.m.; *t* = 15.17; df = 4; ****p < 0.0001; two-tailed Student’s *t* test based on AUC (area under the curve). **d** Outline of the CRISPR screen. ATM wild-type or ATM-deficient cells stably expressing Cas9 nuclease were infected with lentiviral particles containing the whole-genome sgRNA library, subjected to puromycin selection, and passaged to ensure loss of affected protein products. Puromycin-resistant *Atm*^*+/+*^ or *Atm*^−/−^ cells were exposed, respectively, to 400 and 50 nM topotecan for 6 days, and resistant pools isolated. Genomic DNA was extracted from these and from parallel cell cultures treated in the absence of topotecan, and DNA libraries were prepared and sequenced using HiSeq2500. MOI multiplicity of infection. **e**, **f** Classification of the most enriched CRISPR-targeted genes in topotecan-resistant wild-type (WT) (**e**) and *Atm*^−/−^ (**f**) mESCs. Dotted red lines represent positive enrichment false discovery rate (FDR) thresholds. Represented are the names of top hits with highest enrichment scores. The two other specific components of the BRCA1-A complex ranked 201/18,424 (*Brcc3*/*Brcc36*) and 1200/18,424 (*Uimc1*/*Rap80*). All data were analyzed by using MAGeCK and are available in Supplementary Data files 1 and 2. Images are representative of three individual experiments. Panels containing clonogenic survival assays (left) and AUC (right) were generated using GraphPad Prism 7. Bars represent mean ± s.e.m.; ****p < 0.0001; NS = not significant (*p* > 0.05); two-tailed Student’s *t* test following *F* test to confirm equal variance; df = 4. For each clonogenic experiment data is pooled from *n* = 3 individual experiments. Supporting data, including selection of Cas9 clones, crystal violet cell viability assays, library coverage plots and dropout as well as pathway enrichment analysis for both WT and *Atm*^−/−^ cells are presented in Supplementary Figures 1 and 2 and Supplementary Data 3 and 4. Source data are provided as a Source Data file

To better understand cellular responses to seDSBs resulting from replication fork breakdown and how ATM affects such responses, we derived wild-type (WT) and *Atm*-null isogenic mouse embryonic stem cells (mESCs) from an established mouse model of ATM deficiency^17^. As expected, *Atm*^−/−^ mESCs exhibited complete loss of ATM protein and, upon DNA damage induction with CPT or IR, they failed to effectively mediate ATM-dependent signaling as measured by phosphorylation of CHK2 Thr-68 (Fig. 1a). In line with these findings and with previous reports^18^, ATM-deficient mESCs were substantially more sensitive than WT cells to the CPT derivative topotecan, which is used clinically to treat ovarian, cervical, and small-cell lung cancers (Fig. 1b, c).

To explore mechanisms of topotecan resistance in ATM-null cells, we expanded specific WT or *Atm*^−/−^ clones stably expressing active Cas9 nuclease (Supplementary Figure 1a), and transduced them in triplicate with a pooled genome-wide lentiviral CRISPR small-guide RNA (sgRNA) library^19^ at 500-fold coverage. After puromycin selection for successfully transduced cells, we treated the WT and *Atm*^−/−^ mESC populations chronically for 6 days with concentrations of topotecan pre-determined to kill more than 90% of the cells (>IC90; Fig. 1d; Supplementary Figure 1b). Surviving cell pools were then isolated, DNA extracted from them, and the regions encoding sgRNAs PCR amplified and subjected to next-generation DNA-sequencing (see Supplementary Figure 1c for library coverages).

We next analyzed ensuing DNA sequence data by comparing raw sgRNA counts in samples arising from topotecan-treated cells with those from untreated cells. As perhaps expected, the most enriched sgRNAs in topotecan-selected WT cells corresponded to *Top1*, the drug target^20^ (Fig. 1e; Supplementary Figure 1d; Supplementary data file 1; Supplementary data file 2). Interestingly, amongst the other sgRNA sequences enriched in WT cells were those corresponding to the genes encoding the TRRAP, EPC1, EPC2, and ING3 subunits of the NuA4 chromatin-remodeling complex^21^ (Fig. 1e), suggesting that this complex may promote topotecan toxicity in WT cells. Although our screens were optimized to identify genetic suppressors, dropout analyses identified deficiencies in genes involved in DDR-related pathways and in production of the matrisome (extracellular matrix and associated components) as potentially promoting topotecan hypersensitivity in WT and ATM-deficient backgrounds, respectively (Supplementary Figure 2).

In contrast to our results relating to WT cells, in the topotecan-resistant *Atm*^−/−^ cell populations the most enriched sgRNAs targeted genes encoding the core NHEJ factors XRCC4 or LIG4, or the BRCA1-A complex components BRCC45 (BRE), ABRAXAS (FAM175A), and MERIT40 (BABAM1), which have been shown to negatively modulate resection and HRR^22^ (Fig. 1f; Supplementary Figure 1e; Supplementary Data file 3; Supplementary Data file 4). Notably, although sgRNAs corresponding to BRCA1-A complex genes were enriched in topotecan-resistant *Atm*^−/−^ cells, sgRNAs targeting *Brca1* or the genes for factors present in other BRCA1-containing complexes were not (Supplementary Data file 3). While it will be of interest to examine many factors identified in our screens for their impacts on seDSB generation and repair and/or on associated cellular responses, for our ensuing studies, we chose to focus on NHEJ and BRCA1-A components in the context of ATM deficiency.

### NHEJ and BRCA1-A mediate topotecan toxicity in ATM-null cells

**Fig. 2:**
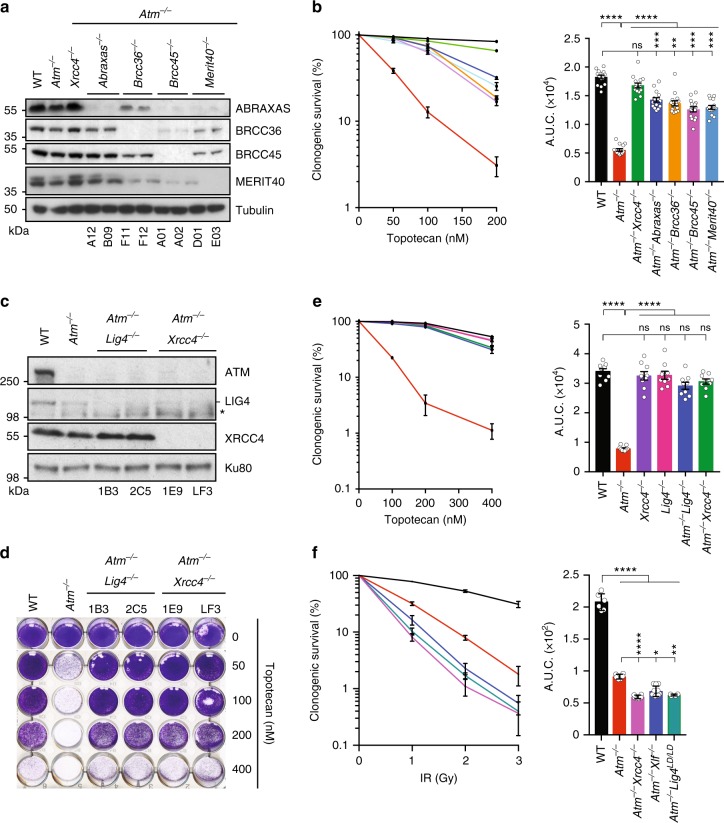
Loss of NHEJ factors or BRCA1-A complex members confers resistance to topotecan in ATM-deficient cells. **a** Representative immunoblot images depicting abundance of ABRAXAS, BRCC36, BRCC45, and MERIT40 proteins in *Atm*^−/−^*Abraxas*^−/−^, *Atm*^−/−^*Brcc36*^−/−^, *Atm*^−/−^*Brcc45*^−/−^, and *Atm*^−/−^*Merit40*^−/−^ cells as compared to *WT*, *Atm*^−/−^, and *Atm*^−/−^*Xrcc4*^−/−^cells. Tubulin is used as a loading control. Two independent clones (numbers identifiers below) were used per genotype. **b** Quantification of clonogenic survival assays towards topotecan in *Atm*^−/−^*Abraxas*^−/−^ (*n* = 15), *Atm*^−/−^*Brcc36*^−/−^ (*n* = 15), *Atm*^−/−^*Brcc45*^−/−^ (*n* = 15), *Atm*^−/−^*Merit40*^−/−^ (*n* = 15), and *Atm*^−/−^*Xrcc4*^−/−^ (*n* = 15) cells as compared to *WT* (*n* = 15) and *Atm*^−/−^ (*n* = 15) cells. **c** Representative immunoblot images depicting abundance of ATM, LIG4 (- indicates LIG4, while * indicates an antibody cross-reacting protein) and XRCC4 proteins in *Atm*^−/−^*Lig4*^−/−^ and *Atm*^−/−^*Xrcc4*^−/−^ cells as compared to *WT* and *Atm*^−/−^ cells. Ku80 is used as a loading control. **d**, **e** Crystal violet cell viability assay (**d**) and quantification of clonogenic survival assays (**e**) indicating suppression of *Atm*^−/−^- dependent (*n* = 9) hypersensitivity to topotecan in *Atm*^−/−^*Lig4*^−/−^ (*n* = 9) and *Atm*^−/−^*Xrcc4*^−/−^ (*n* = 9) cells as compared to *WT* cells (*n* = 9). Note that *Lig4*^−/−^ (*n* = 9) or *Xrcc4*^−/−^ (*n* = 9) single mutants do not exhibit increased topotecan resistance. **f** Quantification of clonogenic survival assays showing that *Atm*^−/−^*Xrcc4*^−/−^ (*n* = 6)*, Atm*^−/−^*Xlf*^−/−^ (*n* = 10), *and Atm*^−/−^*Lig4*^*LD/LD*^ (*n* = 6) cells are more sensitive to IR than *Atm*^−/−^ (*n* = 6) cells. For all clonogenic survival, curves (left) and AUCs (right) were generated by using GraphPad Prism 7 (see Methods). Bars represent mean ± s.e.m.; ****p < 0.0001; ***p < 0.001; **p < 0.01; *p < 0.05; NS = not significant (*p* > 0.05); two-tailed Student’s *t* test following *F* test to confirm equal variance; df = 4 (three independent experiments; *n* = 5 in **b**; *n* = 3 in **e**; *n* = 2 in **f** for each experiment). Additional supporting data, are presented in Supplementary Figure 3. Data for validation of LIG4 or XRCC4 loss suppressing the hypersensitivity of ATM-deficient cells to topotecan in human RPE-1 cells (using both ATM inhibitor and ATM gene knockouts) are presented in Supplementary Figure 4. Source data are provided as a Source Data file

To validate impacts of BRCA1-A components on the topotecan sensitivity of ATM-deficient cells, we used de novo CRISPR-Cas9-mediated gene editing to generate *Atm*^−/−^*Brcc45*^−/−^, *Atm*^−/−^*Merit40*^−/−^, *Atm*^−/−^*Abraxas*^−/−^, and *Atm*^−/−^*Brcc36*^−/−^ double-mutant mESCs (Fig. 2a; as found previously^23–25^, some BRCA1-A components were destabilized in the absence of certain other components). Notably, the absence of each of these BRCA1-A complex proteins markedly suppressed topotecan toxicity in ATM-deficient mESCs (Fig. 2b), thus validating results from the genome-wide CRISPR screen.

Similarly, to validate *Xrcc4* and *Lig4* as suppressor genes in ATM-null cells, we generated *Atm*^−/−^*Lig4*^−/−^ and *Atm*^−/−^*Xrcc4*^−/−^ double-mutant cells by de novo CRISPR-Cas9 gene editing (Fig. 2c; as shown previously^26^, loss of XRCC4 led to LIG4 destabilization). Strikingly, inactivation of *Xrcc4* or *Lig4* in *Atm*^−/−^ mESCs made them almost as resistant to topotecan as WT cells (Fig. 2d, e). Furthermore, re-expression of XRCC4 in *Atm*^−/−^*Xrcc4*^−/−^ cells restored their topotecan hypersensitivity (Supplementary Figure 3a). Importantly, the effects of XRCC4 or LIG4 loss on topotecan resistance seemed to be specific to *Atm*^−/−^ mutant settings, as inactivating *Xrcc4* or *Lig4* in ATM-proficient cells (Supplementary Figure 3b) did not visibly enhance their topotecan resistance (Supplementary Figure 3c) but did confer IR hypersensitivity (Supplementary Figure 3d). Notably, in stark contrast to LIG4 or XRCC4 loss producing topotecan resistance in ATM-deficient cells, we found that combined loss of ATM and either XRCC4 or LIG4 caused cells to be even more sensitive to IR than cells lacking ATM alone (Fig. 2f). As discussed in following sections, these findings likely reflect ATM and NHEJ components playing complementary roles in responding to IR-induced two-ended DSBs, while acting in antagonistic ways at seDSBs arising during DNA replication.

### Topotecan toxicity is mediated by LIG4 catalytic activity

To complement our mESC studies, we generated and validated *ATM*^−/−^, *LIG4*^−/−^, and *ATM*^−/−^*LIG4*^−/−^ clones of TERT-immortalized, non-transformed human RPE-1 cells (Supplementary Figure 4a–g). Ensuing analyses revealed that *ATM*^−/−^*LIG4*^−/−^ RPE-1 cells were significantly more resistant to CPT than *ATM*^−/−^ cells (Supplementary Figure 4h). Moreover, chemical inhibition of ATM kinase activity^27^ in WT RPE-1 cells sensitized them to topotecan, while LIG4 deficiency partially suppressed this phenotype (Supplementary Figure 4i). These results thus indicated that ATM kinase activity functions to prevent CPT-induced cell killing by a mechanism that relies, at least in part, on LIG4.

**Fig. 3:**
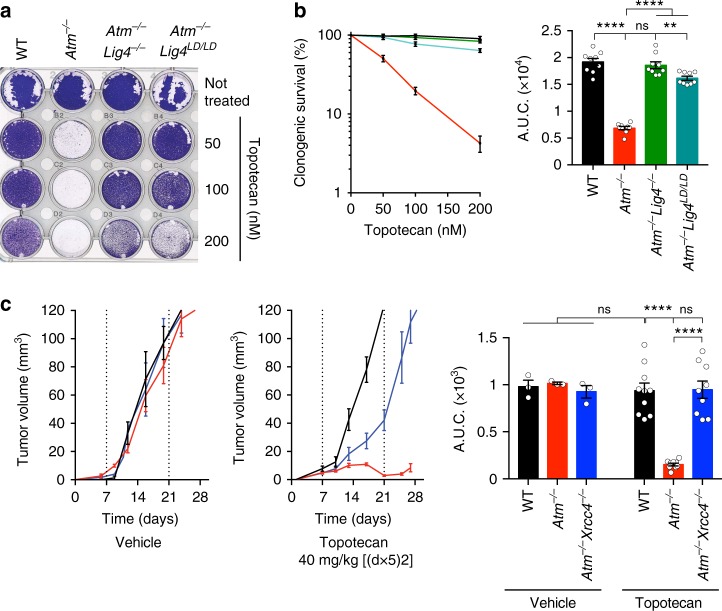
LIG4 catalytic activity mediates topotecan sensitivity in ATM-deficient cells. **a** Crystal violet cell viability assay shows that LIG4 catalytic activity mediates hypersensitivity of ATM-deficient cells to topotecan. LD ligase-dead allele. **b** Quantification of clonogenic survival assays indicating suppression of *Atm*^−/−^ (*n* = 9) cell hypersensitivity to topotecan upon abrogation of LIG4 catalytic activity in *Atm*^−/−^*Lig4*^*LD/LD*^ (*n* = 9) as compared to WT (*n* = 9) and *Atm*^−/−^*Lig4*^−/−^ (*n* = 9). Data from *n* = 3 individual experiments. **c** Mouse xenograft studies indicating that *Atm*^−/−^*Xrcc4*^−/−^-deficient tumors are more resistant than *Atm*^−/−^ single mutant tumors to 40 mg kg^−1^ topotecan treatment (days 1–5 and 8–12 equivalent to [(*d*×5)2] schedule via intraperitoneal (i.p.) injections—see Methods). Growth of untreated tumors (*n* = 3 mice/genotype) was compared to growth of topotecan-treated tumors (*n* = 9 mice/genotype) (left) and AUCs calculated and graphed (right). For the panel containing clonogenic survival assays as well as tumor volume percentage survival curves (left) and AUCs (right) were generated by using GraphPad Prism 7 (see Methods section). Bars represent mean ± s.e.m.; ****p < 0.0001; ***p < 0.001; **p < 0.01; *p < 0.05; NS = not significant (*p* > 0.05); two-tailed Student’s *t* test following *F* test to confirm equal variance; df = 4 in **b**, df = 12 for the untreated and df = 16 for the topotecan-treated mice in **c**. Additional supporting data, including generation of *Lig4* LD allele, and validation of *Lig4*^−/−^ and *Xrcc4*^−/−^ individual knockouts as well as complementation assays, are presented in Supplementary Figure 5. Source data are provided as a Source Data file

Because the absence of XRCC4 resulted in decreased LIG4 protein levels, but not vice versa (Fig. 2c), our findings suggested that LIG4 function is a prime mediator of the hypersensitivity of ATM-null cells to TOP1i. To evaluate whether LIG4 DNA ligase activity per se was required for such hypersensitivity, we assessed the impact of CRISPR-Cas9-mediated genome engineering of the K273A point mutation that is known to abrogate LIG4 catalytic function^28^ (Supplementary Figure 5a, b) into *Atm*^−/−^ mESCs. Strikingly, similar to complete loss of LIG4, this catalytically inactive *Lig4*^*LD/LD*^ allele conferred strong resistance to topotecan (Fig. 3a, b) but not IR (Fig. 2f) when introduced in *Atm*^−/−^ cells. These observations thereby implicated DNA ligation activity, rather than a structural function of the XRCC4-LIG4 complex, as mediating topotecan toxicity in the absence of ATM. To extend our findings into a more physiological setting, we generated mouse tumor xenografts using our set of mESC lines, and treated the mice with topotecan on a previously established schedule^29^. In agreement with our in vitro data, *Atm*^−/−^ tumors were highly sensitive to topotecan when compared to WT controls, while *Atm*^−/−^*Xrcc4*^−/−^ tumors showed significantly increased resistance to the drug as compared to *Atm*^−/−^ tumors (Fig. 3c). Taken together, these findings highlighted how DNA ligase catalytic activity is a major driver for topotecan toxicity in cells lacking functional ATM, and established that LIG4-XRCC4 function confers topotecan hypersensitivity to ATM-deficient cells both in vitro and in vivo.

### Only some NHEJ factors mediate topotecan sensitivity

**Fig. 4:**
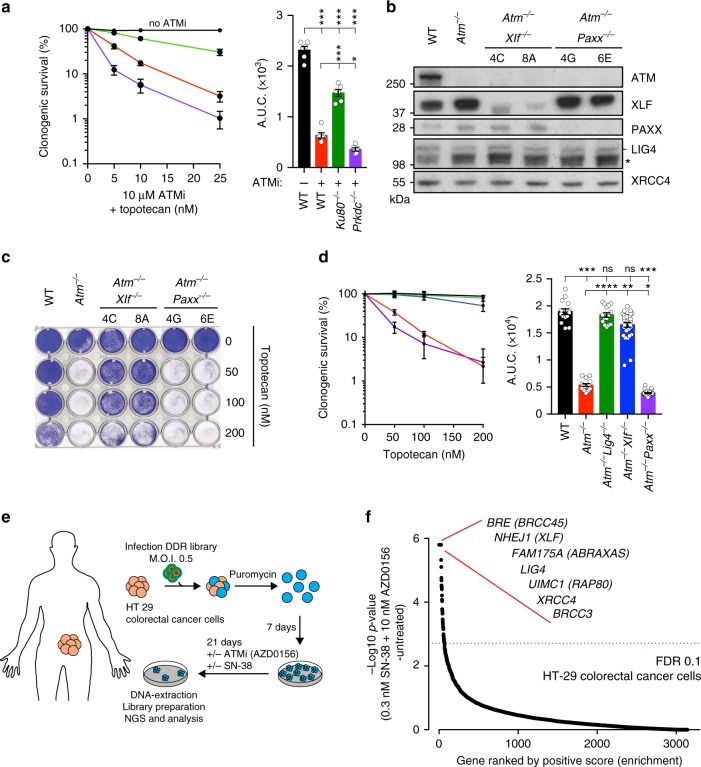
Only certain NHEJ factors are involved in topotecan resistance in ATM-deficient cells. **a** Quantification of clonogenic survival assays showing that inhibiting ATM kinase activity sensitizes WT cells to topotecan and that inactivation of *Xrcc5/Ku80* but not *Prkdc/DNA-PKcs* partially suppresses this phenotype. *n* = 9/genotype. **b** Representative immunoblot images depicting abundance of ATM, XLF, and PAXX proteins in *Atm*^−/−^*Xlf*^−/−^ and *Atm*^−/−^*Paxx*^−/−^ cells as compared to *WT* and *Atm*^−/−^ cells. LIG4 (- indicates LIG4, while * indicates an antibody cross-reacting protein) and XRCC4 was used as loading controls. **c** Crystal violet cell viability assay showing that loss of XLF but not of PAXX ameliorates the hypersensitivity of ATM-deficient cells to topotecan. **d** Quantification of clonogenic survival assays indicating that the hypersensitivity of *Atm*^−/−^ cells to topotecan is alleviated in *Atm*^−/−^*Xlf*^−/−^ but not in *Atm*^−/−^*Paxx*^−/−^ cells (WT and *Atm*^−/−^*Lig4*^−/−^ cells used as controls). *n* = 15/genotype. **e** Outline of the CRISPR screen in cancer cells. HT-29 colorectal cancer cells were infected with lentiviral particles containing a DDR focused sgRNA library, subjected to puromycin selection, and passaged to ensure loss of affected protein products. Puromycin-resistant cells were exposed to 10 nM ATMi (AZD0156) and 0.3 nM Irinotecan (SN-38) for 21 days, and resistant pools were isolated. MOI multiplicity of infection. **f** Classification of the most enriched CRISPR-targeted genes. Dotted red lines represent positive enrichment false discovery rate (FDR) thresholds. Represented are the names of top hits with highest enrichment scores. All data were analyzed by using MAGeCK and are available in Supplementary Data file 5. Images are representative of three individual experiments; individual clone names are represented below the genotypes. **a**, **d** containing clonogenic survival assays (left) and AUCs (right) were generated by using GraphPad Prism 7. Bars represent mean ± s.e.m.; ****p < 0.0001; ***p < 0.001; **p < 0.01; *p < 0.05; NS = not significant (*p* > 0.05); two-tailed Student’s *t* test following *F* test to confirm equal variance; df = 4. Data from *n* = 3 individual experiments. Additional supporting data, including generation of *Ku80*^−/−^ and *Prkdc*^−/−^ individual knockouts, are presented in Supplementary Figure 5. Source data are provided as a Source Data file

Together with XRCC4 and LIG4, another core component of the NHEJ apparatus is the DNA-PK complex, which comprises the Ku70/80 heterodimer and DNA-PKcs^4,30^. As combined genetic inactivation of ATM and DNA-PK is lethal to mouse cells^31^, we generated *Prkdc*-deficient (encoding DNA-PKcs) or *Xrcc6*-deficient (encoding Ku80) mESCs in an otherwise WT background (Supplementary Figure 5c–f), and treated these and control cells with a combination of ATM inhibitor (ATMi) plus various concentrations of topotecan. In contrast to the effect of Ku80 loss (also, see ref. ^32^), DNA-PKcs deficiency did not increase topotecan resistance in the context of ATM inhibition (Fig. 4a). We also studied the impacts of inactivating the proteins XLF and PAXX, which have recently been shown to play partially redundant roles in NHEJ^33–37^. Thus, when we generated and analyzed *Atm*^−/−^*Xlf*^−/−^ and *Atm*^−/−^*Paxx*^−/−^ cells (Fig. 4b), this revealed that absence of XLF, but not PAXX, significantly suppressed topotecan hypersensitivity in the context of ATM deficiency (Fig. 4c, d; because the *Nhej1/Xlf* locus was not well annotated in the mouse genome, it was not represented in the sgRNA library used in our CRISPR-Cas9 screens). Collectively, our data thus indicated that the hypersensitivity of ATM-deficient cells to TOP1i is mediated by toxic reactions arising from a subset of NHEJ components, likely via them promoting LIG4 catalytic activity towards seDSBs arising during DNA replication.

### Resistance to combined TOP1i and ATMi in cancer cells

Combination treatment with ATMi and TOP1i has been proposed to induce synergistic killing of cancer cells^38^. Indeed, combination of the TOP1i SN-38 and the ATMi AZD0156 is currently being evaluated clinically (NCT02588105), with special emphasis in colorectal cancer. To assess whether the resistance mechanisms we identified might be relevant in this setting, we conducted a CRISPR-Cas9 screen in the colorectal cancer cell line HT-29 (Fig. 4e). Strikingly, the top gene hits that suppressed sensitivity to the combined action of SN-38 and AZD0156 encoded proteins of the NHEJ and BRCA1-A complexes (Fig. 4f; for additional hits see Supplementary Data file 5). This indicates conservation of the suppression mechanism in a human cancer cell line and that it not only operates when ATM protein is absent, but also when its catalytic activity is inhibited. Taken together, these data suggest that exploring the genetic status of NHEJ and BRCA1-A components in ATM-deficient tumors, or when exploring drug combinations with ATMi, might help identify which patients are most likely to benefit from agents such as TOP1 inhibitors.

### 53BP1 loss does not suppress TOP1i or PARPi sensitivity of *Atm*-null cells

**Fig. 5:**
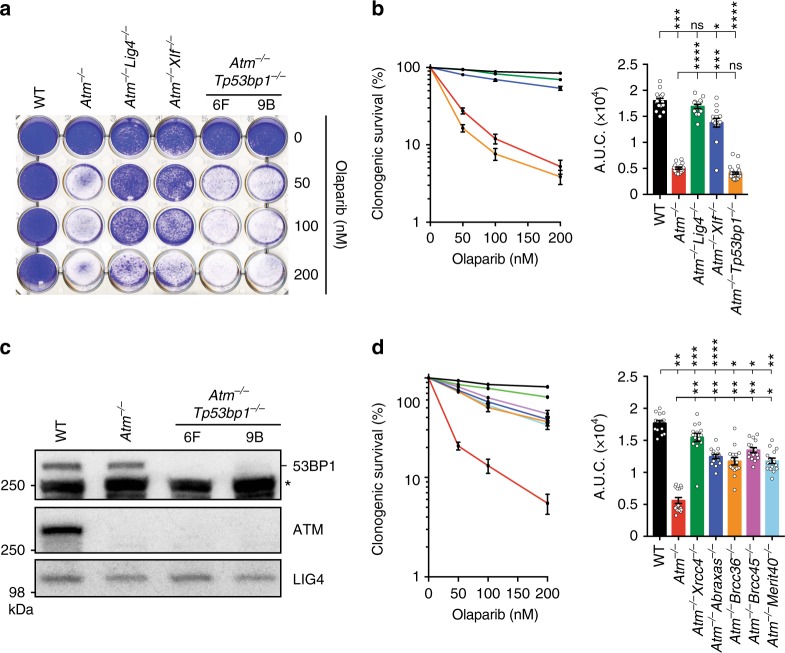
Mechanism of suppression in ATM-deficient cells is different to that in BRCA1-deficient cells. **a** Crystal violet cell viability assay showing that *Atm*-mutant mESCs are hypersensitive to the PARP inhibitor olaparib. Inactivation of *Lig4* or *Xlf*, but not of *Tp53bp1*, rescues the olaparib hypersensitivity of *Atm*-deficient cells. **b** Quantification of clonogenic survival assays showing that loss of XLF (*n* = 15) but not 53BP1 (*n* = 30) can suppress the hypersensitivity of *Atm*^−/−^ cells (*n* = 15) to olaparib as compared to WT control (*n* = 15). **c** Representative immunoblot images depicting 53BP1 (- indicates 53BP1, while * indicates antibody cross-reacting proteins) and ATM protein levels in *Atm*^−/−^*Trp53bp1*^−/−^ cells as compared to WT and *Atm*^−/−^ cells. LIG4 was used as a loading control. **d** Quantification of clonogenic survival assays showing significant rescue of *Atm*^−/−^ dependent sensitivity to olaparib upon loss of individual BRCA1-A complex members in *Atm*^−/−^*Abraxas*^−/−^, *Atm*^−/−^*Brcc36*^−/−^, *Atm*^−/−^*Brcc45*^−/−^, and *Atm*^−/−^*Merit40*^−/−^ cells. *n* = 15/genotype. **b**, **d** containing clonogenic survival curves (left) and AUCs (right) were generated using GraphPad Prism 7. Bars represent mean ± s.e.m.; ****p < 0.0001; ***p < 0.001; **p < 0.01; *p < 0.05; NS = not significant (*p* > 0.05); two-tailed Student’s *t* test following *F* test to confirm equal variance. df = 4 (**b**) and df = 4 (**c**). Data from *n* = 3 individual experiments. Images are representative of three individual experiments; individual clone names are represented below the genotypes. Additional supporting data showing that *Tp53bp1* deficiency cannot rescue hypersensitivity of *Atm*-deficient cells to topotecan are presented in Supplementary Figure 5g. Source data are provided as a Source Data file

Similar to the actions of TOP1i, small-molecule inhibitors of PARP1 cause replication-fork breakage and seDSBs that require HRR in order for cells to survive^15^. In line with this and our findings with topotecan and CPT, we established that *Atm*^−/−^ cells displayed considerable hypersensitivity to the PARP inhibitor, olaparib. Moreover, this hypersensitivity was suppressed by inactivation of *Lig4* or *Xlf* (Fig. 5a, b), suggesting that a similar NHEJ-mediated toxicity mechanism operates for both topotecan and olaparib in ATM-deficient cells.

In experimental and therapeutic contexts, inhibitors of TOP1 or PARP1 have been shown to be particularly toxic to cells with mutations in the HRR genes *BRCA1* or *BRCA2*. Furthermore, there is a growing body of data indicating similar toxicities in cells with mutations in genes such as *ATM* that may share molecular features with *BRCA*-mutant cells^16^. Indeed, terms such as BRCA-ness or HR-deficiency (HRD) are often used to highlight functional similarities between such genetic defects. Intriguingly, however, we noted that our genome-wide screen for topotecan resistance in ATM-null cells did not identify *Tp53bp1*, a gene whose inactivation is well established to restore HRR proficiency to *BRCA1*-mutant cells and thus confer PARP inhibitor resistance^39,40^. In accord with our screening data, we found that unlike loss of LIG4, XRCC4, or XLF, inactivation of 53BP1 (Fig. 5c) did not suppress the hypersensitivity of ATM-deficient cells towards olaparib or topotecan (Fig. 5a, b; Supplementary Figure 5g). Furthermore, in contrast to 53BP1 loss, genetic inactivation of various components of the BRCA1-A complex markedly rescued olaparib hypersensitivity in ATM-deficient cells (Fig. 5d). These data thus suggested that hypersensitivity to olaparib in ATM-deficient cells is mechanistically different from that in BRCA1-deficient settings, where olaparib toxicity seems to arise from an inability to perform HRR even if NHEJ is inactivated^39^.

### ATM-deficient cells exhibit delayed DNA end resection

**Fig. 6:**
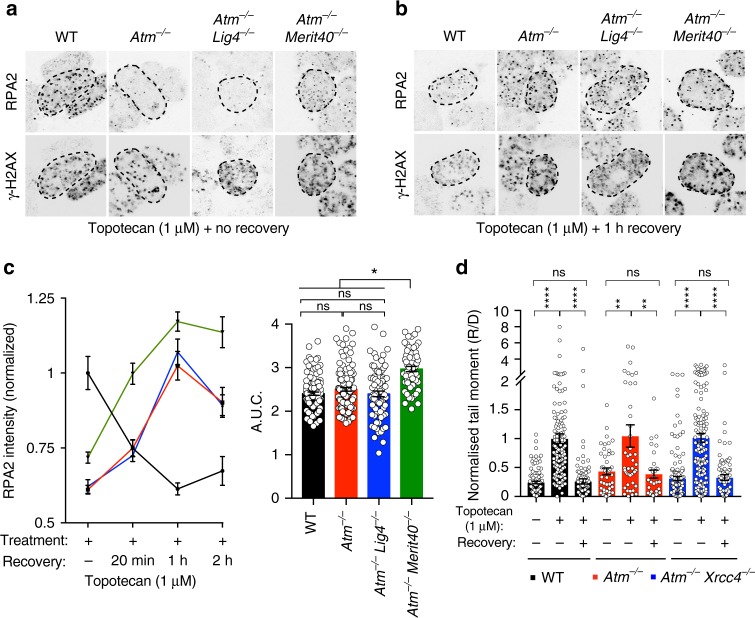
ATM-deficient cells can execute resection and do not accumulate unrepaired seDSBs upon topotecan treatment. **a**, **b** Representative images used for quantification of RPA2 foci accumulation in γH2AX-positive nuclei. *Atm*^−/−^*Lig4*^−/−^ and *Atm*^−/−^*Merit40*^−/−^ cells are compared to WT and *Atm*^−/−^ cells upon topotecan treatment for 1 h (**a**) or 1 h after topotecan was removed (**b**). For better visualization, color images were inverted in black and white; representative color images for all time points are presented in Supplementary Figure 7. Dashed outline indicates periphery of nuclei based on DAPI staining. **c** Quantification of topotecan-induced RPA2 foci formation, showing an initial delay in seDSB end resection in *Atm*^−/−^ (*n* = 122) and *Atm*^−/−^*Lig4*^−/−^ (*n* = 77) cells compared to WT (*n* = 110) cells (no recovery time point), but a recovery in resection during the 2-h timeframe after topotecan withdrawal. Overall, *Atm*^−/−^*Merit40*^−/−^ cells (*n* = 57) show significantly higher resection when compared to all the other genotypes. RPA2 intensity quantifications were analyzed exclusively in γH2AX-positive nuclei, representing S-phase cells that had encountered topotecan-induced TOP1-DNA cleavage complexes during replication. Cells were treated for 1 h with 1 µM topotecan and recovered for 20 min (20 min), 1 h, or 2 h without the drug. Graphs quantifying RPA intensity (left) and AUC (right) were generated by using GraphPad Prism 7. Bars represent mean ± s.e.m.; ****p < 0.0001; NS = not significant (*p* > 0.05); two-tailed Student’s *t* test following *F* test to confirm equal variance; df = 4. Data from *n* = 3 individual experiments. **d** Neutral comet assays showing similar DNA damage generation and repair patterns upon seDSB induction in WT (*n* = 86), *Atm*^−/−^ (*n* = 32), and *Atm*^−/−^*Xrcc4*^−/−^ (*n* = 89) cells. Cells were treated for 1 h with 1 µM topotecan, the drug removed, and cells allowed to recover for a further 6 h. Bar graphs represent the mean ± s.e.m. of the normalized ratio of recovery to damage (R/D) tail moments. The graph was generated by using GraphPad Prism 7; ****p < 0.0001; **p < 0.01; NS = not significant (*p* > 0.05); two-tailed Student’s *t* test following *F* test to confirm equal variance; df = 4. Source data are provided as a Source Data file

HRR of DSBs, such as those created by topotecan or olaparib treatment, starts with DNA end resection. While ATM has been implicated in this process and is widely regarded as a HR-promoting factor, its importance for HRR has recently been disputed, at least in the context of repairing DSBs arising from endonuclease activity^41,42^. To explore the involvement of ATM in HRR of seDSBs, we treated cells with topotecan and then assessed the accumulation of RPA into nuclear foci, a well-established resection marker^43^. To assess the kinetics of this process, intensity of RPA foci was measured after 1 h of acute topotecan exposure, as well as during the following 2 h after drug removal^44^. Notably, compared to WT controls, *Atm*^−/−^ cells displayed significantly reduced intensity of RPA foci 1 h after continuous topotecan treatment, indicative of delayed DNA end resection (Fig. 6a; Supplementary Figure 6a). Importantly, this did not seem to be due to slower replication or generation of less DNA damage in ATM-deficient cells, as co-staining with antibodies recognizing the DNA damage marker Ser-139 phosphorylated histone H2AX (γH2AX), or measurement of replication dynamics by incorporation of the nucleotide analog EdU, did not reveal any significant differences between WT and *Atm*^−/−^ cells (Fig. 6a; Supplementary Figure 6a–c). Strikingly, however, while RPA focus intensity decreased after topotecan withdrawal in WT cells—presumably reflecting replacement of RPA with RAD51 and ensuing HRR—RPA-focus intensity increased during this period in *Atm*^−/−^ cells (Fig. 6b). Indeed, the overall levels of RPA focus formation/intensity during the experiment were very similar in cells containing or lacking ATM (Fig. 6a–c; Supplementary Figure 6a).

Collectively, the above findings implied that ATM deficiency does not prevent DNA resection but instead delays its kinetics. This conclusion is in accord with our findings that there were no significant differences in the generation and repair of seDSBs produced by topotecan between WT and ATM-deficient cells, as assessed by neutral comet assays (Fig. 6d). Furthermore, we found that loss of LIG4 or XRCC4 in *Atm*^−/−^ cells had no perceptible impact on topotecan-induced RPA focus generation or seDSB repair, implying that LIG4 does not markedly affect resection in ATM-deficient settings (Fig. 6c, d; Supplementary Figure 6a). On the other hand, we found that *Atm*^−/−^*Merit40*^−/−^ cells exhibited overall higher levels of RPA-focus intensity than *Atm*^−/−^ or *Atm*^−/−^*Lig4*^−/−^ cells (Fig. 6a–c; Supplementary Figure 6a), a finding in line with the documented role of the BRCA1-A complex in suppressing DSB resection^45,46^.

### ATM-deficient cells mediate HRR of seDSBs

**Fig. 7:**
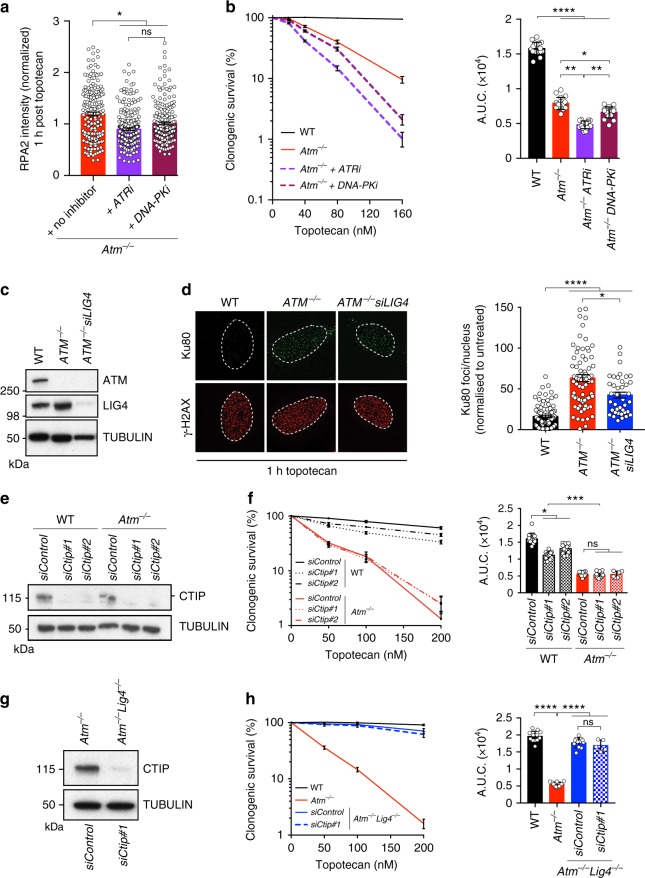
CTIP-dependent Ku removal plays a minor role in toxicity to topotecan in ATM-deficient cells. **a** Bar graph of the extent of RPA2 foci formation in ATM-deficient cells after 1 h recovery from 1 h topotecan treatment (no inhibitor) compared to treatment with topotecan and ATR (ATRi; AZD6738; 1 µM) or DNA-PK (DNA-PKi; NU7441; 3 µM) inhibitors. *n* ≥ 30 replicates in each time point. **b** Quantification of clonogenic survival assays showing significant increased sensitivity of *Atm*^−/−^ cells treated with ATRi or DNA-PKi compared to *Atm*^−/−^ cells (no inhibitor) upon topotecan treatment; *n* = 18/genotype. **c** Representative images of immunoblots showing absence of ATM protein in *ATM*^−/−^ U2OS cells. LIG4 proteins levels were analyzed in *ATM*^−/−^ cells treated with *siLIG4*. **d** Representative images and quantifications of Ku80 foci in γH2AX-positive nuclei in U2OS cells treated with topotecan. Cells were transfected as in **c**. Dashed outline indicates periphery of nuclei. **e** Representative immunoblot analysis of lysates from cells from the representative genotypes upon transfection with siControl as compared to *siCtip #1* and *siCtip #2* and analyzed for CTIP protein levels. **f** Panels containing clonogenic survival assays (left) and AUC (right) upon topotecan treatment in cells depleted of *Ctip* in WT (*Atm*^*+/+*^; *n* = 12 for each siRNA) and *Atm*^−/−^ (*n* = 12 for each siRNA) backgrounds as compared to control treatment with siControl (*n* = 12/genotype). **g** Representative immunoblot analysis of lysates from cells from the representative genotypes upon transfection with siControl as compared to *siCtip #1* and analyzed for CTIP protein levels. **h** Panels containing clonogenic survival assays (left) and AUC (right) upon topotecan treatment in cells depleted of *Ctip* in *Atm*^−/−^ *Lig4*^−/−^ (*Atm*^−/−^ *Lig4*^−/−^; *n* = 12 for each siRNA) as compared to control treatment with siControl (*n* = 12/genotype) in *Atm*^*+/+*^*, Atm*^−/−^ and *Atm*^−/−^ *Lig4*^−/−^ backgrounds. In **b**, **f,** **h**, bars represent mean ± s.e.m; ****p < 0.0001; ***p < 0.001; **p < 0.01; *p < 0.05; NS = not significant (*p* > 0.05); **b** two-tailed Student’s *t* test following *F* test to confirm equal variance**;** df = 4; **f**, **h** one-way Anova**;** df = 5. **a**, **b**, **d** Data from *n* = 3 individual experiments; **f**, **h** data from *n* = 2 individual experiments. Source data are provided as a Source Data file

ATM-dependent phosphorylation of CTIP was recently connected to pathways counteracting Ku loading and persistence at seDSBs, presumably to promote HRR^11^. To investigate whether ATR or DNA-PKcs could perform such phosphorylations in the absence of ATM, we treated cells with topotecan in the absence or presence of selective ATR or DNA-PKcs inhibitors, then generated cell extracts and analyzed them by sodium dodecyl sulfate (SDS)-polyacrylamide gel electrophoresis for CTIP mobility^43,47^. While a DNA-damage-induced shift in CTIP mobility indicative of its phosphorylation was readily apparent in WT cells, this mobility shift was absent in ATM-deficient cells, regardless of whether ATR or DNA-PK was inhibited (Supplementary Figure 7a). Nevertheless, inhibiting the activity of ATR, and to a lesser extent of DNA-PKcs, reduced topotecan-induced RPA focus formation in ATM-deficient cells (Fig. 7a) and further increased the sensitivity of these cells to topotecan (Fig. 7b). Collectively, these findings suggested that while ATR and DNA-PKcs may cooperate with ATM at seDSB sites, they are unable to compensate for ATM loss by restoring normal CTIP phosphorylation.

Recent studies have documented persistent Ku foci at sites of seDSBs in ATM-deficient cells^11,32^, suggesting that the hypersensitivity of such cells to TOP1i reflects defective Ku removal impairing HRR of replication-associated seDSBs^11,32^. However, while we indeed found that ATM-deficient cells displayed increased topotecan-induced Ku foci compared to WT cells^11,32^, depletion of LIG4 only partially reduced their numbers (Fig. 7c, d) despite LIG4 loss almost fully alleviating the topotecan hypersensitivity of ATM-null cells (see Figs. 2 and 3). Taken together with our findings showing that LIG4 catalytic activity drives topotecan toxicity in ATM-deficient cells, these findings suggested that although Ku is connected to driving topotecan toxicity in such settings, this most likely reflects it promoting LIG4/XRCC4 recruitment and LIG4 catalytic activity at seDSBs. In this regard, we noted that ATM inhibition also led to increased XRCC4 foci formation following topotecan treatment (Supplementary Figure 7b). In accord with these observations and given the involvement of CTIP counteracting Ku at seDSBs, we found that CTIP depletion did not further enhance the sensitivity of ATM-deficient cells to topotecan (Fig. 7e, f), thus placing ATM and CTIP in an epistatic relationship in this regard. Furthermore, while CTIP depletion enhanced the sensitivity of WT cells to topotecan (Fig. 7f), it did not significantly affect topotecan sensitivity in *Atm*^−/−^*Lig4*^−/−^ cells (Fig. 7g, h).

**Fig. 8:**
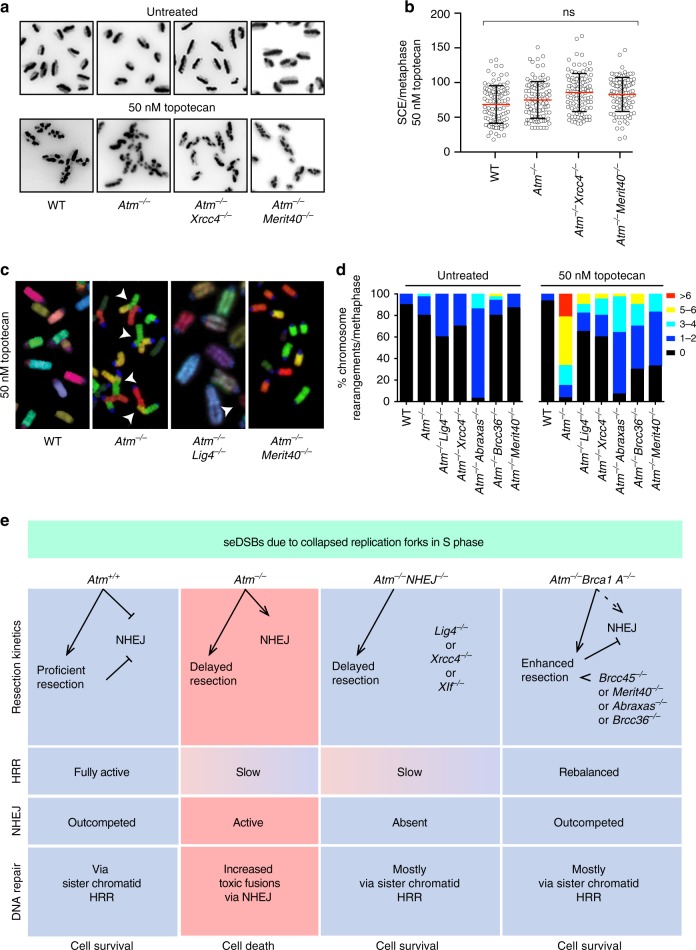
ATM counteracts toxic NHEJ of seDSBs in the S phase. **a**, **b** ATM is not required for BIR-mediated repair of collapsed replication forks. Representative images (**a**) and quantification (**b**) of sister chromatid exchanges (SCEs) in cells treated with 50 nM topotecan (*n* = 100/genotype). Quantifications of chromosome numbers and SCEs in untreated cells are presented in Supplementary Figure 8c, d. Scatter dot plots showing mean ± s.d. number of SCEs across the representative genotypes. Data from *n* = 3 technical replicates. The graph was generated by using GraphPad Prism 7; NS = not significant (*p* > 0.05); one-way ANOVA; total df = 11. **c** ATM is required to prevent toxic fusions upon formation of seDSBs. Representative images of metaphase spreads depicting multicolor fluorescent in situ hybridization (M-FISH) using mouse 21-color painting chromosome probes. White arrows indicate fusions. Representative karyotypes are presented in Supplementary Figure 9a. **d** Contingency graphs showing the percentages of chromosome rearrangements from chromosomal spreads of untreated cells and cells treated with 50 nM topotecan, generated by using GraphPad Prism 7. *n* = 3 technical replicates measuring *n* ≥ 20 metaphases/genotype in each experiment. Statistical analysis is presented in Supplementary Figure 9c. **e** Model for the role of ATM in the repair of seDSBs resulting from collapsed replication forks in S phase of the cell cycle. Column 1, ATM promotes resection of seDSBs, thereby speeding up their repair by HRR and minimizing the time-window during which toxic NHEJ might take place. As shown, ATM also counteracts NHEJ by other mechanisms (see main text). Column 2, in the absence of ATM, seDSB resection is delayed and NHEJ is not suppressed, leading to some seDSBs being subject to illegitimate NHEJ, causing chromosome fusions and ensuing cell death. Column 3, inactivating NHEJ alleviates the hypersensitivity of ATM-null cells to agents that generate seDSBs because toxic, illegitimate NHEJ is absent. Column 4, modifying seDSB end-resection dynamics by loss of BRCA1-A complex components alleviates (rebalances) the seDSB resection defect of ATM-deficient cells, thereby minimizing the potential for illegitimate NHEJ. Source data are provided as a Source Data file

Restart of a broken replication fork that has generated a seDSB (as is the case upon topotecan or olaparib treatment) requires HRR using the homologous sister chromatid as template. This mechanism involves RAD51-dependent strand invasion and formation of a Holliday junction, which upon resolution results in a SCE^14^. It has been recently shown that absence of CTIP or impaired MRE11 exonuclease activity results in defective accumulation of RAD51 onto seDSBs, and this has been suggested to be due to persistence of Ku foci^11^, implying that ATM-deficient cells would show defective SCE formation upon seDSB induction. Strikingly, we found that ATM deficiency did not affect the extent of topotecan-induced SCE formation when compared to WT cells or to *Atm*^−/−^*Xrcc4*^−/−^ or *Atm*^−/−^*Merit40*^−/−^ cells (Fig. 8a, b; for controls showing equivalent cell-cycle progression see Supplementary Figure 6b, c; for karyotypes and SCEs, see Supplementary Figure 7c, d). These results thereby supported our other findings indicating that, although delayed, HRR of seDSBs at broken replication forks can be successfully completed in the absence of ATM, and that overall HRR efficiency is not overtly affected by LIG4/XRCC4 or components of the BRCA1-A complex. Furthermore, they suggested that persistence of Ku at seDSBs in the absence of ATM activity does not overtly impair their repair by HR.

### Toxic NHEJ-mediated chromosomal aberrations in *Atm*^−/−^ cells

Significantly, we observed that while ATM-deficient cells were competent to eventually execute HRR of seDSBs, *Atm*^−/−^ cells treated with topotecan displayed high levels of chromosomal aberrations, especially in the form of radial chromosomes involving fusions of chromatids from different chromosomes (Fig. 8c, d; Supplementary Figure 8a, b). Indeed, continuous exposure to 50 nM topotecan for 24 h resulted in almost all metaphases from *Atm*^−/−^ cells exhibiting at least one chromosomal aberration, while this was the case for only ~5% of WT cells (Fig. 8d, right panel). Crucially, we found that the extent of such chromosomal aberrations in *Atm*^−/−^ cells was markedly reduced in the *Atm*^−/−^*Lig4*^−/−^ or *Atm*^−/−^*Xrcc4*^−/−^ double-mutant backgrounds, and was also substantially reduced in *Atm*^−/−^*Abraxas*^−/−^, *Atm*^−/−^*Brcc36*^−/−^, or *Atm*^−/−^*Merit40*^−/−^ cells (Fig. 8c,d; Supplementary Figure 8c), paralleling the effects we observed on cell survival. Together, these findings supported a model in which topotecan-induced killing of ATM-deficient cells is largely mediated via the formation of chromosomal aberrations by a mechanism(s) that can be circumvented by inactivation of certain NHEJ components or deficiency in proteins specific for the BRCA1-A complex.

## Discussion

*ATM* mutations are found in various cancers^48^ and also cause the neurodegenerative and cancer-predisposition syndrome, ataxia telangiectasia (OMIM; #208900)^49^. Similar to loss of function of breast cancer susceptibility genes *BRCA1* or *BRCA2*, *ATM* loss or mutation causes hypersensitivity to various clinical DNA-damaging agents^50^. In light of these findings and because ATM has been linked to controlling DSB resection, a key early step in HRR, it has been suggested that hypersensitivity of ATM-deficient cancer cells to PARP inhibitors, TOP1i, and other S-phase DNA-damaging agents arises from HRD^16^.

Through CRISPR-Cas9 genetic screening and ensuing studies, we have established that inactivation of genes encoding a subset of classical NHEJ proteins (LIG4, XRCC4, and XLF) or components of the BRCA1-A complex (BRCC45, BRCC36, ABRAXAS, and MERIT40) alleviates the toxicity exerted by TOP1i and PARPi on ATM-null cells. Furthermore, we found that this toxicity is largely mediated by LIG4 catalytic activity, implying that it is not the recruitment or persistence of Ku and the NHEJ machinery at seDSBs per se but lack of NHEJ-mediated DNA end ligation that drives drug sensitivity in ATM-deficient settings. Importantly, and in accord with recently published data^42,51^, we found that ATM-null cells, although presenting delayed resection kinetics, are proficient in HRR of replication-associated seDSBs. Based on these findings, we thus conclude that, unlike wild-type cells, *ATM*-mutant cells fail to prevent some seDSBs being converted into toxic chromosomal aberrations by NHEJ during S phase, with these abnormalities ending up killing cells via apoptosis, mitotic catastrophe, and/or other mechanisms. Accordingly, resistance to TOP1i or PARPi ensues in ATM-deficient cells when such toxic NHEJ is prevented, either directly by loss of NHEJ end-ligation factors or indirectly via inactivating BRCA1-A complex components, which modifies DSB resection dynamics to increase HRR efficiency (Fig. 8e).

The molecular characterization of the resistance mechanisms we have described has important implications for our understanding of seDSB repair. First, our results show that the overall efficiency of HRR of seDSBs is not strongly affected by ATM loss. Second, they also suggest that in the absence of ATM, CTIP is not required for HRR of seDSBs, as its depletion does not further increase sensitivity to topotecan in such a scenario. Third, we observed that HRR of seDSBs as measured by SCEs is not impaired by the absence of ATM, even though Ku foci persist under these circumstances. Fourth, we have established that the absence of LIG4 is sufficient to suppress the sensitivity of ATM-deficient cells to topotecan, even though LIG4 loss has a minor impact on the persistence of Ku at seDSBs. Collectively, these results imply that toxicity to seDSB-inducing agents in ATM-deficient cells is primarily driven by the completion of the ligation step of NHEJ at a limited number of seDSBs, and not by the inability to load RAD51 onto resected DSBs due to inefficient Ku removal. As ATM phosphorylates hundreds of substrates^52^, it may be that it operates at multiple, other levels^11^ to prevent NHEJ and ensure HRR of broken replication forks. Indeed, by carrying out SILAC mass-spectrometry analysis to identify ATM-dependent phosphorylation events upon TOP1i, we discovered over 100 phosphorylation sites that are under ATM control (Supplementary Figure 9a–d, Supplementary Data file 6). These data argue that ATM orchestrates responses to seDSB accumulation at multiple levels that could collectively control resection speed as well as inhibit NHEJ. It will be of interest to investigate the effect of mutation of some of these phosphorylation sites on resection speed as well as on NHEJ activity.

Notably, we found that DNA-PKcs or PAXX deficiency did not rescue the hypersensitivity of ATM-deficient cells to TOP1 or PARP inhibitors, implying that toxic end-joining events at seDSBs can happen independently of these factors. This is also in line with previous findings indicating that unlike XLF, PAXX does not play a significant role in NHEJ in S/G2-phase cells^34^. It is tempting to speculate that this might reflect replication-associated seDSBs being relatively simple substrates that can be ligated together by the actions of the Ku, XRCC4, XLF, and LIG4 proteins without DNA end-processing activities dependent on DNA-PKcs^53^.

The hypersensitivity of Fanconi anemia cells to DNA inter-strand crosslinking agents such as cisplatin or mitomycin C (MMC) has been reported to be mediated by toxic NHEJ^54,55^, although recent work has cast doubt on this conclusion^56^. To test whether a similar mechanism operates in response to seDSB-inducing agents, we treated FA-patient derived FANCD2-deficient cells with topotecan or MMC, and compared their sensitivities to cells complemented with wild-type FANCD2. We found that while FANCD2-deficient cells were hypersensitive to MMC, they exhibited no detectable hypersensitivity to topotecan, arguing that the mechanisms of FA cell killing by these two drugs are inherently different (Supplementary Figure 9e, f; data are for *FANCD2*^−/−^ cells complemented with an empty plasmid vector or vector containing WT *FANCD2*). Furthermore, our other findings have highlighted how the hypersensitivity of ATM-deficient cells to seDSB-inducing agents is mechanistically different from the scenario in BRCA1-deficient cells, where hypersensitivity seems to arise from inability to perform HR even if NHEJ is inactivated^39,40^. Accordingly, the genetic suppression mechanisms that we have defined for *ATM*-mutant cells appear distinct from those reported in *BRCA1*-mutant contexts, where suppression occurs specifically upon inactivation of the DNA-end resection and HR-antagonist protein 53BP1 and its interactors^39,40,57,58^. However, we cannot exclude the possibility that 53BP1 recruitment to seDSBs is promoted by ATM function, as has been shown at double-ended DSBs^59^. Based on the above issues, we suggest caution when interpreting HRD in isolation as a clinical prognostic tool. The HRD score (Myriad Genetics HRD™)^60^ or the HRDetect mutational signature model^61^ have been proposed as predictive biomarkers of response to treatment with agents such as PARP inhibitors, regardless of etiology or mechanism of action. Based on our findings, we suggest that these approaches could miss opportunities presented by deficiencies in ATM and/or in other factors involved in suppressing NHEJ at broken DNA replication forks during S phase^62^.

Intrinsic or acquired tumor cell resistance to established chemotherapeutics, and towards newer molecularly targeted agents such as PARP inhibitors, is a major problem in cancer management^63^. Understanding the molecular bases for drug resistance is thus crucial in order to establish better patient stratification and combination chemotherapy regimens, as well as to better understand mechanisms and relationships between cellular DDR and other processes. Based on our findings, we suggest that exploring the genetic and transcriptional status of genes for BRCA1-A complex members and of the NHEJ components Ku70/80, XRCC4, XLF, and LIG4 in ATM-deficient tumors might help in predicting responses to seDSB-inducing agents such as TOP1 or PARP inhibitors. Moreover, our finding that LIG4 or XRCC4 loss further sensitizes ATM-deficient cells to IR is in line with recent data describing increased sensitivity to IR in BRCA1-deficient cells that have acquired resistance to PARP inhibitors through loss of components of the 53BP1-Shieldin complex^64^. These findings highlight the potential for exploiting a resistance mechanism towards one drug type as a sensitization mechanism towards another therapeutic regime, through a process of acquired vulnerability.

## Methods

### Animals

Care and use of all mice used for this paper was carried out in accordance with UK Home Office regulations, UK Animals (Scientific Procedures) Act of 2013 under UK Home Office licenses which approved this work and is reviewed regularly by the WTSI Animal Welfare and Ethical Review Board and along the ARRIVE guidelines^65^. *Atm (129S6-Atm*^*tm1Awb*^*/J;* stock no:008671) and NSG (NOD.Cg-*Prkdc*^*scid*^ *Il2rg*^*tm1Wjl*^/SzJ; stock no: 005557) knockout (KO) mice were imported from Jackson Laboratories. Mouse genotyping was performed from tail biopsies. Mice were maintained in a specific pathogen-free unit on a 12 h light:12 h dark cycle with lights off at 19:30 and no twilight period. The ambient temperature is 21 ± 2 °C, and the humidity is 55 ± 10%. Mice were housed using a stocking density of 3–5 mice per cage (overall dimensions of caging: 365 × 207 × 140 mm^3^ (length × width × height), floor area 530 cm^2^) in individually ventilated caging (Tecniplast, Sealsafe 1284L) receiving 60 air changes per hour. In addition to Aspen bedding substrate, standard environmental enrichment of two Nestlets, a cardboard fun tunnel, and three wooden chew blocks are provided. Mice were given water and diet ad libitum.

### Tumor xenografts

Tumor xenografts were induced based on established protocols and using the guidelines for the welfare and use of animals in cancer research^66,67^. Briefly, ten million WT, *Atm*^*−/−*^, or *Atm*^*−/−*^*Xrcc4*^*−/−*^ mESC cells suspended in 100 μL 1× phosphate-buffered saline (PBS) and 100 μl Matrigel (Corning^®^ Matrigel^®^ Basement Membrane Matrix, *LDEV-Free, #356234) were injected subcutaneously on one flank of female NSG mice. Following the injections, mice were permitted to recover and monitored daily, including tumor measurement using calipers. Once the majority of tumors reached a threshold size of 200 mm^3^, mice were treated with 40 mg/kg topotecan (days 1–5 and 8–12 equivalent to [(*d*x5)2] schedule) or vehicle (water) via intraperitoneal injections^66^. When mice met humane endpoint criteria or passed over 120 mm^3^ tumor volume, mice were euthanized by CO_2_ asphyxiation. Tissues were collected, and then fixed with 10% neutral-buffered formalin.

### Cell lines, culture conditions, and treatments

*Atm*^*+/+*^ (WT) and *Atm*^−/−^ mESCs were obtained from oocytes of *Atm*^*+/−*^ mice^17^, and NSG mESCs (*Prkdc* mutant) were obtained from NSG mice^68^. Unfertilized oocytes generated by superovulation were isolated at E0.5, with cumulus masses digested using hyaluronidase on a stereomicroscope with heat stage set at 37 °C. Embryos were washed through three drops of M2 medium. For the oocyte activation, 150 µl 100 mM SrCl_2_ and 12 µl 0.5 M EGTA (pH = 8) were added to 3 ml of potassium-supplemented simplex optimised medium (KSOM) (GSM-5140, AMS Biotechnology). The activation medium was sterile filtered and pre-equilibrated in a 60 × 15 mm^2^ center well in vitro fertilization dish without oil overlay in a humidified tissue culture incubator set at 37 °C and 5% CO_2_. Oocytes were moved via mouth pipetting to the activation medium and incubated for 90 min. Oocytes were washed through three drops of M2 with any lysed or fragmented embryos removed before returning to pre-equilibrated KSOM to culture. Embryos were checked at day 3, with any eight-cell embryos transferred to KSOM supplemented with CHIR99021 (3 µM; Abcam ab120890) and PD0325901 (1 µM; Sigma PZ0162) for 24 h before being returned to KSOM for further culture to blastocyst. Only well expanded blastocysts were selected for denuding. Blastocysts with blastocoels expanding to less than half of the embryo were allowed to culture on until well expanded. Blastocysts were frequently denuded on day 5 or 6 post activation. Zonas were removed using pre-warmed (37 °C) acid Tyrode’s solution (T1788, Sigma). An expanded blastocyst with large blastocoel would often take under a minute to lose the zona in warm acid Tyrode’s. Embryos were moved to M2 and observed after 1 min. On occasion the zona appears to go in but can be seen again after rehydrating in M2 and so were put back to acid as many times as necessary to fully denude the zona. Embryos were washed through two more dishes of M2 medium. Subsequently, the parthenogenetic embryos were grown in NDiff 227 neural differentiation medium (Stem Cells Inc.; SCS‐SF‐NB‐02) supplemented with CHIR, PD, and LIF (10 µM, Merk-Millipore; ESG1107) and upon exponential growth passaged to Dulbecco's modified Eagle's medium (DMEM) media (Lonza; BE12-614F) supplemented with fetal bovine serum (Gibco), antibiotics (100× Pen/Strep/Glutamine; Gibco; 10378-016), sodium pyruvate (Gibco;11360-070), β-mercaptoethanol (Sigma, N3148), non-essential amino acids (Gibco; 11140-035), and LIF^69,70^. Upon the first rounds of passage, CHIR and PD were sequentially removed at a three-passage interval to allow for adaptation. All plates and flasks were gelatinized before cell seeding.

Human-immortalized RPE-1 hTERT PuroKO cells (see below) were grown in DMEM F-12 Ham (Sigma) supplemented with glutamine, fetal bovine serum, antibiotics, and sodium pyruvate. U2OS cells were grown in DMEM (Sigma) supplemented with fetal bovine serum and antibiotics. All cells were originally obtained from the ATCC cell repository, and we have authenticated cell lines used in our study by STR profiling, if not otherwise stated. All cells are routinely tested to be mycoplasma free.

Samples treated for immunoblotting were irradiated with 10 Gy IR or with addition of 1 μM CPT or 1 μM topotecan respectively (CPT; Sigma, TPT; Tocris Bioscience) to the medium. IR treatments were performed using a calibrated RX-650 fitted with a 0.5-mm aluminum filter for soft X rays. ATMi, ATR inhibitor and DNA-PK inhibitor (ATMi; KU-55933, Tocris Biosciences, ATRi; AZD6738, AstraZeneca, DNA-PKi; NU7441, Tocris Biosciences) was added 1 h before genotoxic treatment, and samples were collected 1 h after application of DNA-damaging conditions.

### Immunoblotting

Cells were collected in 100–150 μl lysis buffer (50 mM Tris-HCl, pH 7.5, 2% SDS, serine/threonine phosphatase inhibitor cocktail (Sigma-Aldrich), protease inhibitor cocktail (Roche), and 10 mM *N* -ethylmaleimide (Sigma-Aldrich)) and incubated for 5 min at 95 °C. Protein concentrations were determined using a NanoDrop spectrophotometer (Thermo Scientific) at 280 nm. Protein lysates were diluted with 2× Laemmli buffer (120 mM Tric-HCl, pH 6.8, 4% SDS, and 20% glycerol) and SDS–PAGE was performed to resolve proteins on precast NuPAGE Novex 4–12% Bis/Tris gradient gels (Invitrogen). Separated proteins were transferred to nitrocellulose or PVDF membranes (GE Healthcare) and immunoblotted with the indicated antibodies. A list of all antibodies used in this study can be found in Supplementary Table 1. All uncropped images are provided in Supplementary Figure 10a,b c.

### Crystal violet sensitivity assays

Cells were seeded at 125,000 cells per well into 24-well plates, and 24 h after plating were treated with the appropriate drug concentration for 5 days, with daily medium and drug replacement. Topotecan and olaparib were from Tocris Bioscience. When IR treatments were performed, cells were seeded at 500,000 cells per well into 6-well plates, irradiated 24 h after plating, and kept growing until cells turned the culture medium yellow for 2 consecutive days. Surviving cells were fixed and stained with crystal violet.

### Clonogenic survival assays

The day before treatment, cells were seeded in 6-well plates at 500 or 1000 cells per well, dilutions per dose, and three replicates per condition. For inhibition of ATR and DNA-PK, ATR inhibitor (AZD6738, 75 nM, AstraZeneca) and DNA-PK inhibitor (NU7441, 200 nM, Tocris Biosciences) was added 1 h before genotoxic treatment. Upon treatment with the appropriate drug concentration for 5–7 days, cells were stained with crystal violet, and the number of colonies per well was counted and normalized to the initial number of cells. For all experiments, data were normalized to the untreated conditions to consider variations in plating efficiency.

### Generation of Cas9-expressing cells

*Atm*^*+/+*^ and *Atm*^*−/−*^ mESCs were transfected with pPB-LR5.1-EF1a-blast2ACas9^19^ and the *piggyBac* transposase vector pCMV-HyPBase^71^ using TransIT-LT1 transfection reagent (Mirus) and following the manufacturer’s instructions (all transfections described in this work were performed using the same reagent). Forty-eight hours after transfection, selection was applied with 10 μg ml^−1^ blasticidin (Thermo-Fisher; R21009) for 6 days, and resistant colonies were isolated. Cas9 expression was tested by immunoblot using 4–12% Bis-Tris SDS polyacrylamide gels (used for all immunoblot applications in this work). Clones expressing and not expressing Cas9 were tested by transient transfection of pU6-Msh6, a construct produced by cloning of sgRNA DNA sequence templates targeting the mouse *Msh6* gene into the pU6-sgRNA plasmid (a gift from W. Skarnes, The Wellcome Trust Sanger Institute, Cambridge, UK). Forty-eight hours after transfection, cells were treated with 2μM 6-thioguanine (6-TG; Sigma) for 5 days, with daily medium and drug replacement. Disruption of *Msh6* causes resistance to 6-TG^72^, and was used as surrogate for Cas9 activity. Cells were then allowed to recover with no drug for 5 more days, and survivors were stained with crystal violet. Sequences of all sgRNA templates used in this work are in Supplementary Table 2.

### Lentivirus production and transduction

Lentiviral production and transduction^19^ was performed using 3 μg of a lentiviral vector, 9 μg of ViraPower Lentiviral Packaging Mix (Invitrogen), and 12 μl of the PLUS reagent were added to 3 ml of OPTI-MEM (Gibco) and incubated for 5 min at room temperature. Thirty-six microliters of the LTX reagent was then added to the mixture and further incubated for 30 min at room temperature. The transfection complex was added to 80% confluent HEK-293FT cells in a 10-cm dish and incubated for 3 h. The medium was replaced 24 h after transfection. Viral supernatant was harvested 48 h after transfection and stored at −80 °C. Transduction of mESCs was performed in suspension as follows: 15,000 mESCs and diluted virus were mixed in 100 μl of the mESC medium containing 8 μg ml^−1^ polybrene (Millipore), incubated for 30 min at 37 °C in a well of a round-bottomed 96-well plate, plated onto a well of a feeder-containing 96-well plate, and cultured until functional analyses. Transduction volumes were scaled up according to the areas of the culture plates if necessary.

### Screening for resistance to topotecan

mESCs/genotypes (10 × 10^7^) were independently infected with the genome-wide guide RNA (gRNA) lentiviral library at a multiplicity of infection of 0.1–0.2, at a library coverage 1000×. Three days after infection, puromycin (10 µM; Gibco A11138-02) was added to the media. Upon established puromycin resistance, cells were partitioned into three independent replicates and cultured for 10 additional days. Upon passage a minimum of 50 × 10^6^ cells per technical replica was maintained at a library coverage of 500×. For each of the three technical replicates, 50 × 10^6^ cells were pooled and the representation sample was saved; 11 × 10^7^ library-infected mESCs/genotypes (in 11 15-cm plates; 1 × 10^6^ cells per plate) and 2 × 10^7^ non-library-infected mESCs/genotypes were treated with topotecan (400 nM for *Atm*^*+/+*^; 50 nM for *Atm*^−/−^) for 6 days, and further cultured for 4 additional days. Surviving cells were pooled per technical replicate, and genomic DNA was extracted and used for PCR templates. One plate per condition was fixed and stained with crystal violet and is represented in Supplementary Figure 1C.

HT-29 colorectal cancer cells were infected with lentiviral particles containing the whole-genome sgRNA library, subjected to puromycin selection, and passaged to ensure loss of affected protein products. Puromycin-resistant cells were exposed to 10 nM ATMi (AZD0156) and 0.3 nM irinotecan (SN-38) for 21 days, and resistant pools were isolated. Genomic DNA was extracted from these and from parallel cell cultures treated in the absence of topotecan, and DNA libraries were prepared and sequenced.

### Illumina sequencing of gRNAs and statistical analysis

Genomic DNA was extracted and gRNAs sequenced as described previously^19^. Single-end Illumina sequencing reads of 19 nucleotides were counted for each gRNA using in-house written software. Depleted or enriched genes in the drug-treated samples were determined from a comparison of read counts with the respective representation sample using the software package MAGeCK^73^ version 0.5.3. A gene set enrichment analysis using MAGeCK indicated overrepresented pathways as annotated in the Molecular Signatures Database (MSigDB)^74^ version 5.2. The gene symbols file (c2.cp.v5.2.symbols.gmt from http://software.broadinstitute.org/gsea/downloads.jsp) of the “all canonical pathways” curated gene sets (C2) was used as reference input file for MAGeCK. Raw sequencing data will be made available upon acceptance of the manuscript.

### Gene editing

*Atm*^−/−*Tg(Cas9)*^ mESCs were transfected with the appropriate Cas9-sgRNA-expressing plasmid (see Supplementary Figure 1 and Supplementary Table 2). Transfected populations aimed to produce *Atm*^−/−^*Lig4*^−/−^ and *Atm*^−/−^ *Xrcc4*^−/−^ mESCs were cultured for five passages, and then plated into 6-well plates at 500,000 cells per well. Twenty-four hours after seeding, cells were treated with 100 nM topotecan for 5 days with daily medium and drug replacement. Cells were then allowed to recover with no drug and surviving colonies were picked into 96-well plates, and expanded for immunoblot testing. One colony from each Cas9-sgRNA vector for each gene was used for further experiments. Transfected populations aimed to produce *Atm*^−/−^*Xlf*^−/−^, *Atm*^−/−^*Paxx*^−/−^, *Atm*^−/−^*Tp53bp1*^−/−^, *Atm*^−/−^*Abraxas1*^−/−^, *Atm*^−/−^*Babam1*^−/−^, *Atm*^−/−^*Brcc3*^−/−^, and *Atm*^−/−^*Bre*^−/−^ mESCs were sorted based on green fluorescence into 6-well plates at 500, 1000, and 2000 cells per well, in duplicate, using a MoFlo flow sorter (Beckman Coulter) or a SH800Z flow sorter (Sony) 48 h after transfection. Ninety-six of the sorted colonies (48 from each of the two different targeting constructs) were transferred to a 96-well plate, triplicated, and genomic DNA was extracted from one of the replicas as described previously^75^. Diagnostic PCRs were performed using 1 μl of genomic DNA as template, and run on 3% 1× Tris-acetate-EDTA agarose gels. Clones producing PCR products showing obvious differences in size compared to the expected were expanded and tested on immunoblots.

To produce catalytic-dead LIG4 cell lines, *Atm*^−/− *Tg(Cas9)*^ mESCs were transfected with a combination of pAiO-WT-Lig4-2 and a 200 bp single-stranded oligonucleotide (ssODN) spanning the region containing the LIG4 catalytic site Lys-273 at a 6:1 ratio. The ssODN contained mutations Lys-273-Ala (placed in the middle of the ssODN) and several others impairing sgRNA annealing and producing a recognition site for the *Afe*I DNA restriction enzyme (Supplementary Figure 2e). Forty-eight hours after transfection, cells were sorted based on green fluorescence into 6-well plates at 500, 1000, and 2000 cells per well, in duplicate, using a MoFlo flow sorter (Beckman Coulter). Ninety-six of the sorted colonies were transferred to a 96-well plate, triplicated, and genomic DNA was extracted from one of the replicas as described previously^75^. Diagnostic PCR was performed using 1 μl of genomic DNA as template, and PCR products were digested using *Afe*I to identify edited products (Supplementary Figure 2f). Sequences of diagnostic PCR oligonucleotides used in this work are in Supplementary Table 3.

To produce *Lig4*, *Prkdc*, *Xrcc4*, and *Xrcc5 (Ku80)* mutants in the WT background, *Atm*^*+/+*^ mESCs were transfected with a combination of the appropriate Cas9-sgRNA plasmid targeting the gene of interest (Supplementary Table 2) and pU6-Hprt at a 1:1 ratio, or pU6-Hprt plus pAiO-Cas9 WT^3^ to generate *Hprt*^−/−^ cells. Disruption of *Hprt* causes resistance to 6-TG^4^, and selection with the drug allowed faster identification of double-mutant cells. Forty-eight hours after transfection, cells were treated with 2 μM 6-TG for 5 days, with daily medium and drug replacement. Cells were then allowed to recover with no drug for 5 more days, and surviving clones were picked, expanded, and tested on immunoblots for double gene disruption.

RPE-1 hTERT PuroKO cells were produced by transient transfection of pAiO-Cas9^D10A5^, pU6-Puro-1, and pU6-Puro-2 (Supplementary Table 2) by electroporation using the Neon Transfection System according to the manufacturer’s instructions (Life Technologies). Forty-eight hours after transfection, single GFP-expressing cells were sorted into 96-well plates using a MoFlo flow sorter (Beckman Coulter), expanded, and tested for their sensitivity to 1 μg ml^−1^ puromycin. Sensitive clones were expanded and used for further experiments.

To produce *ATM*^−/−^ and *LIG4*^−/−^ human cell lines, RPE-1 hTERT PuroKO cells were transfected with the appropriate Cas9-sgRNA plasmid (Supplementary Table 2) as described above, except that they were tested on IMMUNOBLOT for the absence of protein product directly with no diagnostic PCR step. *ATM*^−/−^ *LIG4*^−/−^ cells were produced by transfecting *ATM*^−/−^ cells (clone 21) with pAiO-NK-LIG4 (Supplementary Table 2), running diagnostic PCRs (Supplementary Table 3), and testing selected clones on immunoblots for absence of protein product as described above.

### siRNA transfection

Small interfering RNA (siRNA) transfections were performed using Lipofectamine RNAiMAX (Life Technologies). Cells were reverse transfected at a final siRNA concentration of ∼60 nM, transfection was repeated 24 h after the first transfection, and cells were assayed 48–72 h after transfection. As a negative control, we used siRNA oligonucleotides targeting Luciferase (siLuc). A list of all siRNAs used in this study can be found in Supplementary Table 4.

### Chromosome preparation, staining, and analysis

Chromosome preparation was performed using a standard protocol^76^. For SCE, cells were incubated with bromodeoxyuridine (5 μg ml^−1^; Sigma 19-160) over two passages and stained following an established protocol^77^. For multiplexed fluorescence in situ hybridization (M-FISH) analysis, mouse chromosome-specific DNA libraries were generated from 5000 copies of flow-sorted chromosomes, provided by the Flow Cytometry Core Facility of Wellcome Trust Sanger Institute, using GenomePlexWhole Genome Amplification (WGA2) kit (Sigma-Aldrich, Dorset, UK). Mouse 21-color painting probe was made following the pooling strategy. Five chromosome pools were each labeled with ATTO 425-, ATTO 488-, CY3-, CY5-, and Texas Red-dUTPs (Stratech, Newmarket, UK), respectively, using WGA3 re-amplification kit (Sigma-Aldrich) as described before^78^. The labeled products were pooled and sonicated to achieve a size range of 200–1000 bp, optimal for use in chromosome painting. To make 10 tests of M-FISH probe, 500 μl sonicated DNA was precipitated down together with 100 μl mouse Cot-1 DNA (Invitrogen) and re-suspended in 120 μl hybridization buffer (50% formamide, 2×  saline-sodium citrate (SSC), 10% dextran sulfate, 0.5 M phosphate buffer, 1× Denhardt’s solution, pH 7.4). Metaphase preparations were dropped onto pre-cleaned microscopic slides, and then fixed in acetone (Sigma-Aldrich) for 10 min followed by dehydration through an ethanol series (70%, 90%, and 100%). Metaphase spreads on slides were denatured by immersion in an alkaline denaturation solution (0.5 M NaOH, 1.0 M NaCl) for approximately 40 s, followed by rinsing in 1 M Tris-HCl (pH 7.4) solution for 3 min, 1× PBS for 3 min, and dehydration through a 70%, 90%, and 100% ethanol series. The M-FISH probe (10 μl for each 22 × 22-mm hybridization area) was denatured at 65 °C for 10 min before being applied onto the denatured slides. The hybridization area was sealed with a 22 × 22mm^2^ coverslip and rubber cement. Hybridization was carried out in a 37 °C incubator for 40–44 h. The post-hybridization washes included a 5-min stringent wash in 0.5× SSC at 75 °C, followed by a 5-min rinse in 2× SSC containing 0.05% Tween-20 (VWR) and a 2-min rinse in 1× PBS, both at room temperature. Finally, slides were mounted with SlowFade^®^ Diamond Antifade Mountant containing 4′,6-diamidino-2-phenylindole (DAPI, Invitrogen). Images were visualized on a Zeiss AxioImager D1 fluorescent microscope equipped with narrow band-pass filters for DAPI, DEAC, FITC, CY3, TEXAS RED, and CY5 fluorescence and an ORCA-EA CCD camera (Hamamatsu). M-FISH digital images were captured using the SmartCapture software (Digital Scientific UK, Cambridge, UK) and processed using the SmartType Karyotyper software (Digital Scientific UK). At least 20 metaphases from each sample were fully karyotyped based on M-FISH and enhanced DAPI banding.

### Neutral comet assay

For neutral comet assay^79^ cells were seeded the day before treatment. After topotecan treatment (1μM for 1 h), cells were washed, and if required, recovered for 6 h in media without topotecan. Following trypsinization, cells were resuspended in PBS (−) (Gibco) at a concentration of 2 × 10^5^ cells per ml. Seventy-five microliters of cell suspension were mixed in 500 μl LMAgarose (Trevigen), placed on gel bon films, covered with a 22-mm cover slide (VWR International), and left in the dark for 15 min at 4 °C. After removal of the coverslip, cells were lysed in the dark for 1 h in Trevigen lysis at 4 °C. Following washing with TBE (90 mM Tris-borate (pH 8.3) and 2 mM EDTA), the samples were subjected to electrophoresis at 35 V for 7 min in TBE. Afterwards, cells were fixed in 70% ethanol and dried at room temperature. The following day, nuclei were stained with SYBR green I (Invitrogen) in 10 mM Tris-HCl, pH 7.5, 1 mM EDTA, pH 8.0. Images were taken with an IX70 fluorescent microscope (Olympus) with the Cell F software (Olympus). Relative tail moments were measured using the CometScore software (TriTek). For each condition, tail moments of at least 50 cells were measured.

### Immunofluorescence

Cells were washed with PBS containing 0.1 % Tween-20 (PBST), followed by pre-extraction for 10 min with the CSK buffer (cytoskeleton buffer) containing 25 mM HEPES, pH 7.4, 50 mM NaCl, 3 mM MgCl_2_, 300 mM sucrose, and 0.5% Triton X-100. For RAD51 staining, cells were pre-extracted in the CSK buffer for 4 min on ice. To visualize KU80 and GFP-XRCC4 by immunofluorescence, cells were washed in PBS before pre-extraction in modified CSK buffer (containing 10 mM PIPES, pH 7, 100 mM NaCl, 300 mM sucrose, 3 mM MgCl_2_, 0.7 % Triton X-100, and 0.3 mg ml^−1^ RNase A) twice for 3 min. After 20 min fixation with 2% paraformaldehyde (w v^−1^) in PBS and following blocking in PBST containing 5% bovine serum albumin (BSA) (wv^−1^), primary antibody incubation with the corresponding antibodies (in 5% BSA PBST) was performed for 1 h at room temperature (RT) or at 4 °C overnight. For KU80 and GFP-XRCC4 immunofluorescence prior to blocking, coverslips were reduced for 7 min in 0.1% NaBH_4_, washed in PBS, and permeabilized for 5 min in 0.2% Triton X-100/PBS. After washing with PBST, cells were incubated with the corresponding secondary antibodies, diluted in 5% BSA PBST, and counterstained with DAPI (2 mg ml^−1^). After washing in PBST the cells were mounted using Vectashield (Vector Labs). Fluorescence microscopy was performed using a FluoView 1000 confocal microscope (Olympus).

### High-resolution imaging using deconvolution microscopy

High-resolution imaging was performed by imaging z-stacks containing the whole-cell nucleus with a wide-field Deltavision PersonalDV microscope (Applied Precision, 1024 × 1024 CoolSNAP HQ or HQ2 camera, z-stack of 0.2 mm interval) equipped with a 60× UPlanSApo/1.40 oil objective (Olympus). Deconvolutions were then performed with SoftWoRx (Applied Precision) in a conservative mode. KU80 and GFP-XRCC4 foci were counted by using the find object tool in Volocity 6.3 (Perkin Elmer).

### DNA-damage checkpoint activation

RPE-1 *ATM*^*+/+*^ and *ATM*^−/−^ (clones 1 and 21) cells were seeded into 6-cm dishes (500,000 cells per plate) and treated or not with 10 Gy IR and 10μM ATMi. Eight hours after irradiation, cells were collected, fixed in ice-cold 70% ethanol, and stained with propidium iodide (PI). Cell-cycle profiles were obtained using a Fortessa cell analyzer (BD Biosciences) and produced using the FlowJo software (Tree Star).

### Mass-spectrometry analysis

Phosphoproteome analysis was performed as described previously^80^. Briefly, cells were cultured in SILAC media containing either l-arginine and l-lysine, l-arginine [13C6] and l-lysine [2H4], or l-arginine [13C615N4] and l-lysine [13C6-15N2] (Cambridge Isotope Laboratories)^81^. Cells were pretreated with 10 µM ATMi KU-55933 (Selleckchem) for 1 h prior to treatment with 5 µM CPT (Sigma) for 2 h. Subsequently, cells were lysed in modified RIPA buffer (50 mM Tris, pH 7.5, 650 mM NaCl, 1 mM EDTA, 1% NP-40, 0.1% sodium deoxycholate) supplemented with protease inhibitors and phosphatase (Sigma). Lysates were cleared by centrifugation, proteins were precipitated in fourfold excess of ice-cold acetone, and subsequently re-dissolved in denaturation buffer (6 M urea, 2 M thiourea in 10 mM HEPES, pH 8.0). Cysteines were reduced with 1 mM dithiothreitol and alkylated with 5.5 mM chloroacetamide. Proteins were digested with endoproteinase Lys-C (Wako Chemicals) and sequencing grade-modified trypsin (Sigma) and peptides were purified using reversed-phase Sep-Pak C18 cartridges (Waters). For the enrichment of phosphorylated peptides, 5 mg of peptides in binding buffer (50% acetonitrile, 6% trifluoroacetic acid in H_2_O) were incubated with 10 mg of TiO_2_ spheres (GL Sciences) for 1 h. The beads were washed twice in binding buffer and subsequently peptides were eluted using elution buffer (10% NH_4_OH, 25% acetonitrile in H_2_O). Peptides were fractionated using micro-column-based strong-cation exchange chromatography and desalted on reversed-phase C18 StageTips^82^.

Peptide fractions were analyzed on a quadrupole Orbitrap mass spectrometer (Q Exactive Plus, Thermo Scientific) equipped with a UHPLC system (EASY-nLC 1000, Thermo Scientific)^83^. Peptide samples were loaded onto C18 reversed-phase columns (15 cm length, 75 µm inner diameter, 1.9 µm bead size) and eluted with a linear gradient from 8 to 40% acetonitrile containing 0.1% formic acid in 2 h. The mass spectrometer was operated in data-dependent mode, automatically switching between MS and MS^2^ acquisition. Survey full-scan MS spectra were acquired in the Orbitrap. The ten most intense ions were sequentially isolated and fragmented by higher energy C-trap dissociation^84^. Fragment spectra were acquired in the Orbitrap mass analyzer. Raw data files were analyzed using MaxQuant (development version 1.5.2.8)^85^. Parent ion and MS2 spectra were searched against a database containing 92,578 human protein sequences obtained from the UniProtKB released in December 2016 using Andromeda search engine^85^. Cysteine carbamidomethylation was searched as a fixed modification, whereas protein N-terminal acetylation, methionine oxidation, and phosphorylation of serine, threonine, and tyrosine were searched as variable modifications. Site localization probabilities were determined by MaxQuant using the PTM scoring algorithm as described previously^85^. The dataset was filtered based on posterior error probability to arrive at a false discovery rate below 1% estimated using a target-decoy approach^86^. Only phosphorylated peptides with a score ≥40, delta score ≥8, score difference ≥5, and localization probability ≥0.75 were consider for downstream analysis. Functional protein interaction network analysis was performed using interaction data from the STRING database^87^. Only interactions with a score >0.7 are represented in the networks. Cytoscape version 3.1.1 was used for visualization of protein interaction networks.

### Statistics

All graphs and part of the statistical analysis in the manuscript (two-tailed Student’s *t* tests; area under curve; Fisher's exact test) were generated and calculated using GraphPad Prism version 7.0a for Mac OS X, GraphPad Software (La Jolla, CA, USA, www.graphpad.com). Area under the curve (AUC) graphs were generated using the integrated Prism 7 formula without any modification (where the baseline is considered Y = 0); detailed description of the method can be found in the GraphPad Statistics Guide/AUC. For the CRISPR screen data analysis, the statistics were calculated from a comparison of read counts with the respective representation sample using the software package MAGeCK^73^ version 0.5.3.

For all panels containing immunoblots, the raw images used can be visualized in Supplementary Figure 10a–c.

## Supplementary information

Supplementary Information

Description of Additional Supplementary Files

Supplementary Data 1

Supplementary Data 2

Supplementary Data 3

Supplementary Data 4

Supplementary Data 5

Supplementary Data 6

Reporting Summary

Source data

## Data Availability

All relevant data are available from the authors upon request. The mass-spectrometry data can be downloaded from ProteomeXchange via the PRIDE database using PXD011108 project accession identifier. The source data underlying Figs. 1a, 2b, e, f, 3b, c, 4a, d, 5b, d, 6c, d, 7a, b, d, f, h and 8b, d are provided as a Source Data file.
