# Supplementary material for: ATM orchestrates the DNA-damage response to counter toxic non-homologous end-joining at broken replication forks
Source: Nat Commun. 2019 Jan 8;10:87. doi: 10.1038/s41467-018-07729-2 (PMC6325118; doi:10.1038/s41467-018-07729-2)
Supplement: Supplementary file 1 — Supplementary Information [file 41467_2018_7729_MOESM1_ESM.pdf]

Supplementary Materials including:

Materials and Methods

Supplementary Tables 1 – 4

Supplementary Figures 1 - 10

ATM orchestrates the DNA-damage response to counter toxic non-homologous end-joining at broken replication forks

Gabriel Balmus[1,2,6,‡], Domenic Pilger[1,‡], Julia Coates[1], Mukerrem Demir[1], Matylda Sczaniecka-Clift[1], Ana Barros[2], Michael Woods[2], Beiyuan Fu[2], Fengtang Yang[2], Elisabeth Chen[2], Matthias Ostermaier[3], Tatjana Stankovic[4], Hannes Ponstingl[2], Mareike Herzog[1,2], Kosuke Yousa[2], Francisco Munoz Martinez[1], Stephen T. Durant[5], Yaron Galanty[1], Petra Beli[3], David J. Adams[2], Allan Bradley[2], Emmanouil Metzakopian[2,6], Josep V. Forment[1,5,7,‡], and Stephen P. Jackson[1,7]

[1]The Wellcome Trust and Cancer Research UK Gurdon Institute and Department of Biochemistry, University of Cambridge, Tennis Court Road, Cambridge CB2 1QN, UK.

[2]Wellcome Trust Sanger Institute, Hinxton, Cambridge CB10 1SA, UK.

[3]Institute of Molecular Biology (IMB), 55128 Mainz, Germany.

[4]Institute of Cancer and Genomic Sciences, College of Medical and Dental Sciences, University of Birmingham, Edgbaston, Birmingham B15 2TT, UK.

[5]DNA Damage Response Biology, Bioscience Oncology IMED Biotech Unit, AstraZeneca, Cambridge CB4 0WG, UK.

[6]UK Dementia Research Institute at University of Cambridge, Department of Clinical Neurosciences, University of Cambridge, Cambridge CB2 0AH, UK.

[‡]These authors contributed equally to this work.

[7]Correspondence should be addressed. Correspondence to s.jackson@gurdon.cam.ac.uk; josep.forment@astrazeneca.com

Supplementary Materials and Methods

Animals

Care and use of all mice used for this paper was carried out in accordance with UK Home Office regulations, UK Animals (Scientific Procedures) Act of 2013 under UK Home Office licenses which approved this work and is reviewed regularly by the WTSI Animal Welfare and Ethical Review Board and along the ARRIVE guidelines[1]. *Atm (129S6-Atm^{tm1Awb}/J;* stock no:008671) and NSG (NOD.Cg-*Prkdc^{scid} Il2rg^{tm1Wjl}*/SzJ; stock no: 005557) knock-out (KO) mice were imported from Jackson Laboratories. Mouse genotyping was performed from tail biopsies. Mice were maintained in a specific pathogen-free unit on a 12h light: 12h dark cycle with lights off at 19:30 and no twilight period. The ambient temperature is 21 ± 2 °C, and the humidity is 55 ± 10%. Mice were housed using a stocking density of 3–5 mice per cage (overall dimensions of caging: 365 × 207 × 140 mm (length × width × height), floor area 530 cm2) in individually ventilated caging (Tecniplast, Sealsafe 1284L) receiving 60 air changes per hour. In addition to Aspen bedding substrate, standard environmental enrichment of two Nestlets, a cardboard fun tunnel and three wooden chew blocks are provided. Mice were given water and diet ad libitum.

Tumor Xenografts

Tumor xenografts were induced based on established protocols and using the guidelines for the welfare and use of animals in cancer research[2,3]. Briefly, ten million WT, *Atm$^{-/-}$* or *Atm$^{-/-}$Xrcc4$^{-/-}$* mESC cells suspended in 100 µL 1 × PBS and 100 µl Matrigel (Corning® Matrigel® Basement Membrane Matrix, *LDEV-Free, #356234) were injected subcutaneously on one flank of female NSG mice. Following the injections, mice were permitted to recover and monitored daily, including tumor

measurement using calipers. Once the majority of tumors reached a threshold size of 200 mm^3, mice were treated with 40mg/kg topotecan (days 1-5 and 8-12 equivalent to [(dx5)2] schedule) or vehicle (water) *via* intraperitoneal injections (i.p.)[2]. When mice met humane endpoint criteria or passed over 120mm^3 tumor volume, mice were euthanized by CO_2 asphyxiation. Tissues were collected, fixed with 10% neutral-buffered formalin.

Cell lines, culture conditions and treatments

Atm$^{+/+}$ (wild type; WT) and *Atm*$^{-/-}$ mouse embryonic stem cells (mESCs) were obtained from oocytes of *Atm*$^{+/-}$ mice[4], and NSG mESCs (*Prkdc* mutant) were obtained from NSG mice[5]. Unfertilised oocytes generated by superovulation were isolated at E0.5, with cumulus masses digested using hyaluronidase on a stereomicroscope with heat stage set at 37°C. Embryos were washed through 3 drops of M2 medium. For the oocyte activation, 150µl 100mM $SrCl_2$ and 12µl 0.5M EGTA (pH=8) were added to 3ml of KSOM (GSM-5140, AMS Biotechnology). The activation medium was sterile filtered and pre- equilibrated in a 60 x 15mm centre well IVF dish without oil overlay in a humidified tissue culture incubator set at 37°C and 5% CO_2. Oocytes were moved via mouth pipetting to the activation medium and incubated for 90 minutes. Oocytes were washed through 3 drops of M2 with any lysed or fragmented embryos removed before returning to pre-equilibrated KSOM to culture. Embryos were checked at day 3, with any 8 cell embryos transferred to KSOM supplemented with CHIR99021 (3µM; Abcam ab120890) and PD0325901 (1µM; Sigma PZ0162) for 24 hours before being returned to KSOM for further culture to blastocyst. Only well expanded blastocysts were selected for denuding. Blastocysts with blastocoels expanding to less than half of the embryo were allowed to culture on until well expanded. Blastocysts were frequently

denuded on day 5 or 6 post activation. Zonas were removed using pre-warmed (37°C) acid Tyrode's solution (T1788, Sigma). An expanded blastocyst with large blastocoel would often take under a minute to lose the zona in warm acid Tyrode's. Embryos were moved to M2 and observed after 1 minute. On occasion the zona appears to go in but can be seen again after rehydrating in M2 and so were put back to acid as many times as necessary to fully denude the zona. Embryos were washed through 2 more dishes of M2 medium. Subsequently the parthenogenetic embryos were grown in NDiff 227 neural differentiation medium (Stem Cells Inc.; SCS-SF-NB-02) supplemented with CHIR, PD and LIF (10μM, Merk-Millipore; ESG1107) and upon exponential growth passaged to DMEM media (Lonza; BE12-614F) supplemented with, fetal bovine serum (Gibco), antibiotics (100X Pen/Strep/Glutamine; Gibco; 10378-016), sodium pyruvate (Gibco;11360-070), beta-mercaptoethanol (Sigma, N3148), non-essential amino acids (Gibco; 11140-035) and LIF[6,7]. Upon the first rounds of passage CHIR and PD were sequentially removed at a 3-passage interval to allow for adaptation. All plates and flasks were gelatinized before cell seeding.

Human immortalized RPE-1 hTERT PuroKO cells (see below) were grown in DMEM F-12 Ham (Sigma) supplemented with glutamine, fetal bovine serum, antibiotics and sodium pyruvate. U2OS cells were grown in DMEM (Sigma) supplemented with fetal bovine serum and antibiotics. HT29 cells were obtained from ECACC (Cat# 91072201). All cells were originally obtained from the ATCC cell repository, and we have authenticated cell lines used in our study by STR profiling, if not otherwise stated. All cells are routinely tested to be mycoplasma free.

Samples treated for western blotting were irradiated with 10 Gy ionizing radiation (IR) or with addition of 1 μM camptothecin or 1 μM topotecan respectively (CPT; Sigma, TPT; Tocris Bioscience) to the medium. IR treatments were performed using a

calibrated RX-650 fitted with a 0.5-mm aluminum filter for soft X rays. ATM inhibitor, ATR inhibitor and DNA PK inhibitor (ATMi; KU55933, Tocris Biosciences, ATRi; AZD6738, AstraZeneca, DNA PKi; NU7441, Tocris Biosciences) was added 1 h before genotoxic treatment, and samples were collected 1 h after application of DNA damaging conditions.

siRNA transfection

siRNA transfections were performed using Lipofectamine RNAiMAX (Life Technologies). Cells were reverse transfected at a final siRNA concentration of ~60 nM, transfection was repeated 24 h after the first transfection and cells were assayed 48–72 h after transfection. As a negative control, we used siRNA oligonucleotides targeting Luciferase (siLuc).

Crystal violet sensitivity assays

Cells were seeded at 125 000 cells/well into 24-well plates, and 24 h after plating were treated with the appropriate drug concentration for 5 days, with daily medium and drug replacement. Topotecan and olaparib were from Tocris Bioscience. When IR treatments were performed, cells were seeded at 500 000 cells/well into 6-well plates, irradiated 24 h after plating, and kept growing until cells turned the culture medium yellow for 2 consecutive days. Surviving cells were fixed and stained with crystal violet.

Clonogenic survival assays

The day before treatment, cells were seeded in 6-well plates at 500 or 1000 cells/well, dilutions per dose and three replicates per condition. For inhibition of ATR and DNA PK, ATR inhibitor (AZD6738, 75 nM, AstraZeneca) and DNA PK inhibitor (NU7441,

200 nM, Tocris Biosciences) was added 1 h before genotoxic treatment. Upon treatment with the appropriate drug concentration for 5 – 7 days, cells were stained with crystal violet, and the number of colonies per well was counted and normalized to the initial number of cells. For all experiments, data were normalized to the untreated conditions to take into account variations in plating efficiency.

Generation of Cas9 expressing cells

Atm$^{+/+}$ and^{-} *Atm*$^{-/-}$ mESCs were transfected with pPB-LR5.1-EF1a-blast2ACas9[8] and the *piggyBac* transposase vector pCMV-HyPBase[9] using TransIT-LT1 transfection reagent (Mirus) and following manufacturer's instructions (all transfections described in this work were performed using the same reagent). 48 h after transfection, selection was applied with 10 μg/ml blasticidin (Thermo-Fisher; R21009) for 6 days, and resistant colonies were isolated. Cas9 expression was tested by western blotting (WB) using 4-12% Bis-Tris SDS polyacrylamide gels (used for all WB applications in this work). Clones expressing and not expressing Cas9 were tested by transient transfection of pU6-Msh6, a construct produced by cloning of sgRNA DNA sequence templates targeting the mouse *Msh6* gene into the pU6-sgRNA plasmid (a gift from W. Skarnes, The Wellcome Trust Sanger Institute, Cambridge, UK). 48 h after transfection, cells were treated with 2 μM 6-thioguanine (6-TG; Sigma) for 5 days, with daily medium and drug replacement. Disruption of *Msh6* causes resistance to 6-TG[10], and was used as surrogate for Cas9 activity. Cells were then allowed to recover with no drug for 5 more days, and survivors were stained with crystal violet. Sequences of all sgRNA templates used in this work are in Supplementary Table 2.

Lentivirus production and transduction.

Lentiviral production and transduction was as previously described [8]. Briefly, 3 μg of a lentiviral vector, 9 μg of ViraPower Lentiviral Packaging Mix (Invitrogen) and 12 μl of the PLUS reagent were added to 3 ml of OPTI-MEM (Gibco) and incubated for 5 min at room temperature. 36 μl of the LTX reagent was then added to the mixture and further incubated for 30 min at room temperature. The transfection complex was added to 80% confluent HEK-293FT cells in a 10-cm dish and incubated for 3 h. Medium was replaced 24 h after transfection. Viral supernatant was harvested 48 h after transfection and stored at −80 °C. Transduction of mESCs was performed in suspension as follows: 15,000 mESCs and diluted virus were mixed in 100 μl of the mESC medium containing 8 μg ml^{-1} polybrene (Millipore), incubated for 30 min at 37 °C in a well of a round-bottomed 96-well plate, plated onto a well of a feeder-containing 96-well plate and cultured until functional analyses. Transduction volumes were scaled up according to the areas of the culture plates if necessary.

Screening for resistance to topotecan

10 × 10^7 mESCs/genotype were independently infected with the genome-wide gRNA lentiviral library at an MOI of 0.1-0.2, at a library coverage 1000X. Three days after infection, puromycin (10 μM; Gibco A11138-02) was added to the media. Upon established puromycin resistance, cells were partitioned in 3 independent replicates and cultured for 10 additional days. Upon passage a minimum of 50 x 10^6 cells/technical replica was maintained at a library coverage of 500X. For each of the 3 technical replicates, 50 x 10^6 cells were pooled and the representation sample was saved; 11 × 10^7 library-infected mESCs/genotype (in 11 15-cm plates; 1x10^6 cells/plate) and 2 x 10^7 non-library infected mESCs/genotype were treated with topotecan (400 nM for *Atm*$^{+/+}$; 50 nM for *Atm*$^{-/-}$) for 6 days, and further cultured for 4

additional days. Surviving cells were pooled per technical replicate, and genomic DNA was extracted and used for PCR templates. 1 plate per condition was fixed and stained with crystal violet and is represented in **Supplementary Figure 1c**. The screen was independently repeated and similar results were obtained (data not shown).

HT-29 colorectal cancer cells were infected with lentiviral particles containing the whole-genome sgRNA library, subjected to puromycin selection, and passaged to ensure loss of affected protein products. Puromycin-resistant cells were exposed to 10nM ATMi (AZD0156) and 0.3nM Irinotecan (SN-38) for 21 days, and resistant pools were isolated. Genomic DNA was extracted from these and from parallel cell cultures treated in the absence of topotecan, and DNA libraries were prepared and sequenced.

Illumina sequencing of guide RNAs and statistical analysis

Genomic DNA was extracted and guide RNAs sequenced as described previously[8]. Single-end Illumina sequencing reads of 19 nucleotides were counted for each gRNA using in-house written software. Depleted or enriched genes in the drug-treated samples were determined from a comparison of read counts with the respective representation sample using the software package MAGeCK[11] version 0.5.3. A gene set enrichment analysis using MAGeCK indicated overrepresented pathways as annotated in the Molecular Signatures Database (MSigDB)[12] version 5.2. The gene symbols file (c2.cp.v5.2.symbols.gmt from http://software.broadinstitute.org/gsea/downloads.jsp) of the 'all canonical pathways' curated gene sets (C2) was used as reference input file for MAGeCK. Raw sequencing data will be made available upon acceptance of the manuscript.

Gene editing

Atm$^{-/-Tg(Cas9)}$ mESCs were transfected with the appropriate Cas9-sgRNA expressing plasmid (see Supplementary Figure S1 and Supplementary Table 2). Transfected populations aimed to produce *Atm*$^{-/-}$*Lig4*$^{-/-}$ and *Atm*$^{-/-}$ *Xrcc4*$^{-/-}$ mESCs were cultured for 5 passages, and then plated into 6-well plates at 500 000 cells/well. 24 h after seeding, cells were treated with 100 nM topotecan for 5 days with daily medium and drug replacement. Cells were then allowed to recover with no drug and surviving colonies were picked into 96-well plates, and expanded for WB testing. One colony from each Cas9-sgRNA vector for each gene was used for further experiments. Transfected populations aimed to produce *Atm*$^{-/-}$*Xlf*$^{-/-}$, *Atm*$^{-/-}$*Paxx*$^{-/-}$, *Atm*$^{-/-}$*Tp53bp1*$^{-/-}$, *Atm*$^{-/-}$ *Abraxas1*$^{-/-}$, *Atm*$^{-/-}$*Babam1*$^{-/-}$, *Atm*$^{-/-}$*Brcc3*$^{-/-}$ and *Atm*$^{-/-}$*Bre*$^{-/-}$ mESCs were sorted based on green fluorescence into 6-well plates at 500, 1000 and 2000 cells/well, in duplicate, using a MoFlo flow sorter (Beckman Coulter) or a SH800Z flow sorter (Sony) 48 h after transfection. 96 of the sorted colonies (48 from each of the two different targeting constructs) were transferred to a 96-well plate, triplicated, and genomic DNA was extracted from one of the replicas as described previously[13]. Diagnostic PCRs were performed using 1 μl of genomic DNA as template, and run on 3% 1x Tris-acetate-EDTA agarose gels. Clones producing PCR products showing obvious differences in size compared to the expected were expanded and tested on WB.

To produce catalytic-dead LIG4 cell lines, *Atm*$^{-/-Tg(Cas9)}$ mESCs were transfected with a combination of pAiO-WT-Lig4-2 and a 200 bp single-stranded oligonucleotide (ssODN) spanning the region containing the LIG4 catalytic site Lys-273 at a 6:1 ratio. The ssODN contained mutations Lys-273-Ala (placed in the middle of the ssODN) and several others impairing sgRNA annealing and producing a recognition site for the *Afe*I DNA restriction enzyme (Supplementary Figure S2E). 48 h after transfection, cells

were sorted based on green fluorescence into 6-well plates at 500, 1000 and 2000 cells/well, in duplicate, using a MoFlo flow sorter (Beckman Coulter). 96 of the sorted colonies were transferred to a 96-well plate, triplicated, and genomic DNA was extracted from one of the replicas as described previously[13]. Diagnostic PCR was performed using 1 μl of genomic DNA as template, and PCR products were digested using *Afe*I to identify edited products (Supplementary Figure S2F). Sequences of diagnostic PCR oligonucleotides used in this work are in Supplementary Table 3.

To produce *Lig4, Prkdc, Xrcc4* and *Xrcc5 (Ku80)* mutants in the WT background, $Atm^{+/+}$ mESCs were transfected with a combination of the appropriate Cas9-sgRNA plasmid targeting the gene of interest (Supplementary Table 2) and pU6-Hprt at a 1:1 ratio, or pU6-Hprt plus pAiO-Cas9 WT[3] to generate $Hprt^{-/-}$ cells. Disruption of *Hprt* causes resistance to 6-TG[4], and selection with the drug allowed faster identification of double mutant cells. 48 h after transfection, cells were treated with 2 μM 6-TG for 5 days, with daily medium and drug replacement. Cells were then allowed to recover with no drug for 5 more days, and surviving clones were picked, expanded and tested on WB for double gene disruption.

RPE-1 hTERT PuroKO cells were produced by transient transfection of pAiO-Cas9^{D10A}[5], pU6-Puro-1 and pU6-Puro-2 (Supplementary Table 2) by electroporation using the Neon Transfection System according to the manufacturer's instructions (Life Technologies). 48 h after transfection, single GFP-expressing cells were sorted into 96-well plates using a MoFlo flow sorter (Beckman Coulter), expanded, and tested for their sensitivity to 1 μg/ml puromycin. Sensitive clones were expanded and used for further experiments.

To produce $ATM^{-/-}$ and $LIG4^{-/-}$ human cell lines, RPE-1 hTERT PuroKO cells were transfected with the appropriate Cas9-sgRNA plasmid (Supplementary Table 2) as

described above, except that they were tested on WB for absence of protein product directly with no diagnostic PCR step. *ATM*$^{-/-}$ *LIG4*$^{-/-}$ cells were produced by transfecting *ATM*$^{-/-}$ cells (clone 21) with pAiO-NK-LIG4 (Supplementary Table 2), running diagnostic PCRs (Supplementary Table 3), and testing selected clones on WB for absence of protein product as described above.

Chromosome preparation, staining and analysis

Chromosome preparation was performed using a standard protocol[14]. For sister chromatid exchange (SCE), cells were incubated with BrdU (5 μg/ml; Sigma 19-160) over two passages and stained following an established protocol[15]. For Multiplexed Fluorescence In-Situ Hybridization (M-FISH) analysis, mouse chromosome-specific DNA libraries were generated from 5,000 copies of flow-sorted chromosomes, provided by the Flow Cytometry Core Facility of Wellcome Trust Sanger Institute, using GenomePlexWhole Genome Amplification (WGA2) kit (Sigma-Aldrich, Dorset, UK). Mouse 21-color painting probe was made following the pooling strategy. Five chromosome-pools were each labeled with ATTO 425-, ATTO 488-, CY3-, CY5-, and Texas Red-dUTPs (Stratech, Newmarket, UK), respectively, using WGA3 re-amplification kit (Sigma-Aldrich) as described before[16]. The labeled products were pooled and sonicated to achieve a size range of 200–1,000 bp, optimal for use in chromosome painting. To make 10 tests of M-FISH probe, 500 μl sonicated DNA was precipitated down together with 100 μl mouse Cot-1 DNA (Invitrogen) and re-suspended in 120 μl hybridization buffer (50% formamide, 2 × SSC, 10% dextran sulfate, 0.5 M phosphate buffer, 1 × Denhardt's solution, pH 7.4). Metaphase preparations were dropped onto pre-cleaned microscopic slides, then fixed in acetone (Sigma-Aldrich) for 10 min followed by dehydration through an ethanol series (70%,

90%, and 100%). Metaphase spreads on slides were denatured by immersion in an alkaline denaturation solution (0.5 M NaOH, 1.0 M NaCl) for approximately 40 s, followed by rinsing in 1 M Tris-HCl (pH 7.4) solution for 3 min, 1 × PBS for 3 min, and dehydration through a 70%, 90%, and 100% ethanol series. The M-FISH probe (10 µl for each 22 × 22-mm hybridization area) was denatured at 65°C for 10 min before being applied onto the denatured slides. The hybridization area was sealed with a 22 × 22-mm coverslip and rubber cement. Hybridization was carried out in a 37 °C incubator for 40 - 44 h. The post-hybridization washes included a 5-min stringent wash in 0.5 × SSC at 75°C, followed by a 5-min rinse in 2 × SSC containing 0.05% Tween20 (VWR) and a 2-min rinse in 1 × PBS, both at room temperature. Finally, slides were mounted with SlowFade® Diamond Antifade Mountant containing 4',6-diamidino-2-phenylindole (DAPI, Invitrogen). Images were visualized on a Zeiss AxioImager D1 fluorescent microscope equipped with narrow band-pass filters for DAPI, DEAC, FITC, CY3, TEXAS RED, and CY5 fluorescence and an ORCA-EA CCD camera (Hamamatsu). M-FISH digital images were captured using the SmartCapture software (Digital Scientific UK, Cambridge, UK) and processed using the SmartType Karyotyper software (Digital Scientific UK). At least 20 metaphases from each sample were fully karyotyped based on M-FISH and enhanced DAPI-banding.

<u>Neutral comet assay</u>

Neutral comet assay was performed as previously described[17]. In brief, cells were seeded the day before treatment. After topotecan treatment (1μM for 1h), cells were washed and if required, recovered for 6h in media without topotecan. Following trypsinization, cells were resuspended in PBS (-) (Gibco) at a concentration of 2x 10^5 cells/ml. 75 μl of cell suspension were mixed in 500 μl LMAgarose (Trevigen), placed

on gel bon films, covered with a 22-mm cover slide (VWR International) and left in the dark for 15 min at 4°C. After removal of the cover slip, cells were lysed in the dark for 1h in Trevigen lysis at 4°C. Following washing with TBE (90 mM Tris-borate (pH 8.3) and 2 mM EDTA), the samples were subjected to electrophoresis at 35 V for 7min in TBE. Afterwards, cells were fixed in 70 % ethanol and dried at room temperature. The following day, nuclei were stained with SYBR green I (Invitrogen) in 10 mM Tris-HCL pH 7.5, 1mM EDTA pH8.0. Images were taken with an IX70 fluorescent microscope (Olympus) with Cell F software (Olympus). Relative tail moments were measured using CometScore software (TriTek). For each condition, tail moments of at least 50 cells were measured.

Immunofluorescence

Cells were washed with PBS containing 0.1 % Tween20 (PBST), followed by pre-extraction for 10 min with CSK-buffer (cytoskeleton-buffer) containing 25mM HEPES pH7.4, 50 mM NaCl, 3mM $MgCl_2$, 300 mM sucrose and 0.5 % Triton X-100. For RAD51 staining, cells were pre-extracted in CSK-buffer for 4 min on ice. To visualize KU80 and GFP-XRCC4 by immunofluorescence, cells were washed in PBS before pre-extraction in modified CSK-buffer (containing 10 mM PIPES pH 7, 100 mM NaCl, 300 mM sucrose, 3 mM $MgCl_2$, 0.7 % Triton X-100 and 0.3 mg ml^{-1} RNase A.) twice for 3 min. After 20 min fixation with 2 % paraformaldehyde (w/v) in PBS and following blocking in PBST containing 5 % bovine serum albumin (BSA) (w/v), primary antibody incubation with the corresponding antibodies (in 5% BSA PBST) was performed for 1h at room temperature (RT) or at 4°C overnight. For KU80 and GFP-XRCC4 immunofluorescence prior to blocking, coverslips were reduced for 7 min in 0.1 % $NaBH_4$, washed in PBS and permeabilised for 5 min in 0.2 % Triton X-100/PBS. After

washing with PBST, cells were incubated with the corresponding secondary antibodies, diluted in 5 % BSA PBST, and counterstained with DAPI (2 mg/ml). After washing in PBST the cells were mounted using Vectashield (Vector Labs). Fluorescence microscopy was performed using a FluoView 1000 confocal microscope (Olympus).

High-resolution imaging using deconvolution microscopy

High-resolution imaging was performed by imaging z-stacks containing the whole-cell nucleus with a wide-field Deltavision PersonalDV microscope (Applied Precision, 1,024 x 1,024 CoolSNAP HQ or HQ2 camera, z-stack of 0.2 mm interval) equipped with a 60x UPlanSApo/1.40 oil objective (Olympus). Deconvolutions were then performed with SoftWoRx (Applied Precision) in conservative mode. KU80 and GFP-XRCC4 foci were counted by using the find object tool in Volocity 6.3 (Perkin Elmer).

DNA-damage checkpoint activation

RPE-1 $ATM^{+/+}$ and $ATM^{-/-}$ (clones 1 and 21) cells were seeded into 6-cm dishes (500 000 cells/plate) and treated or not with 10 Gy IR and 10 μM ATMi. 8 h after irradiation, cells were collected, fixed in ice-cold 70% ethanol, and stained with propidium iodide (PI). Cell cycle profiles were obtained using a Fortessa cell analyzer (BD Biosciences) and produced using FlowJo software (Tree Star).

Mass spectrometry analysis

Phosphoproteome analysis was performed as described previously[18]. Briefly, cells were cultured in SILAC media containing either L-arginine and L-lysine, L-arginine [13C6] and L-Lysine [2H4], or L-arginine [13C615N4] and L-lysine [13C6-15N2]

(Cambridge Isotope Laboratories) [19]. Cells were pretreated with 10 µM ATM inhibitor KU-55933 (Selleckchem) for 1 h prior to treatment with 5 µM camptothecin (Sigma) for 2 h. Subsequently, cell were lysed in modified RIPA buffer (50 mM Tris pH 7.5, 650 mM NaCl, 1 mM EDTA, 1% NP-40, 0.1% sodium deoxycholate) supplemented with protease inhibitors and phosphatase (Sigma). Lysates were cleared by centrifugation, proteins were precipitated in fourfold excess of ice-cold acetone and subsequently re-dissolved in denaturation buffer (6 M urea, 2 M thiourea in 10 mM HEPES pH 8.0). Cysteines were reduced with 1 mM dithiothreitol and alkylated with 5.5 mM chloroacetamide. Proteins were digested with endoproteinase Lys-C (Wako Chemicals) and sequencing grade-modified trypsin (Sigma) and peptides were purified using reversed-phase Sep-Pak C18 cartridges (Waters). For the enrichment of phosphorylated peptides, 5 mg of peptides in binding buffer (50% acetonitrile, 6% trifluoroacetic acid in H_2O) were incubated with 10 mg of TiO_2 spheres (GL Sciences) for 1 h. The beads were washed twice in binding buffer and subsequently peptides were eluted using elution buffer (10% NH_4OH, 25% acetonitrile in H_2O). Peptides were fractionated using micro-column-based strong-cation exchange chromatography and desalted on reversed-phase C18 StageTips [20].

Peptide fractions were analyzed on a quadrupole Orbitrap mass spectrometer (Q Exactive Plus, Thermo Scientific) equipped with a UHPLC system (EASY-nLC 1000, Thermo Scientific)[21]. Peptide samples were loaded onto C18 reversed phase columns (15 cm length, 75 µm inner diameter, 1.9 µm bead size) and eluted with a linear gradient from 8 to 40% acetonitrile containing 0.1% formic acid in 2 h. The mass spectrometer was operated in data dependent mode, automatically switching between MS and MS^2 acquisition. Survey full-scan MS spectra were acquired in the Orbitrap. The ten most intense ions were sequentially isolated and fragmented by higher energy

C-trap dissociation (HCD)[22]. Fragment spectra were acquired in the Orbitrap mass analyzer. Raw data files were analyzed using MaxQuant (development version 1.5.2.8)[23]. Parent ion and MS2 spectra were searched against a database containing 92,578 human protein sequences obtained from the UniProtKB released in December 2016 using Andromeda search engine[23]. Cysteine carbamidomethylation was searched as a fixed modification, whereas protein N-terminal acetylation, methionine oxidation and phosphorylation of serine, threonine and tyrosine were searched as variable modifications. Site localization probabilities were determined by MaxQuant using the PTM scoring algorithm as described previously[23]. The dataset was filtered based on posterior error probability to arrive at a false discovery rate below 1% estimated using a target-decoy approach[24]. Only phosphorylated peptides with a score ≥40, delta score ≥8, score difference ≥5 and localization probability ≥0.75 were consider for downstream analysis. Functional protein interaction network analysis was performed using interaction data from the STRING database[25]. Only interactions with a score > 0.7 are represented in the networks. Cytoscape version 3.1.1 was used for visualization of protein interaction networks.

Statistics

All graphs and part of the statistical analysis in the manuscript (Two-tailed Student's t-tests; Area Under Curve; Fishers exact test) were generated and calculated using GraphPad Prism version 7.0a for Mac OS X, GraphPad Software, La Jolla California USA, www.graphpad.com. Area under the curve (A.U.C.) graphs were generated using the integrated Prism 7 formula without any modification (where the baseline is considered Y=0); detailed description of the method can be found in the GraphPad Statistics Guide/ Area under the curve. For the CRISPR screen data analysis the

statistics were calculated from a comparison of read counts with the respective representation sample using the software package MAGeCK[11] version 0.5.3.

Antigen	Antibody	Application (dilution)	Species
53BP1	Novus NB100-304	WB 1:5000	Mouse
ABRAXAS1	Bing Wang lab	WB 1:10000	Mouse
ATM	Serotec AHP392	WB 1:1000	Mouse
	Epitomics 1549-1	WB 1:10000	Human
ATM pS1981	Abcam ab81292	WB 1:5000	Human
BABAM1/MERIT40	Cell Signaling 12711	WB 1:1000	Mouse
BRCC3/BRCC36	Abcam ab207913	WB: 1:1000	Mouse
BRE/BRCC45	Cell Signaling 12457S	WB 1:1000	Mouse
Cas9	Diagenode C15200203	WB 1:2000	N/A
CHK2	Millipore 05-649	WB 1:2000	Mouse
CHK2 pT68	Cell Signaling 2661	WB 1:1000	Mouse
CTIP	Richard Baer lab	WB 1:40	Mouse/Human
DNAPKcs	Thermo Scientific MA5-13404	WB 1:200	Mouse
GFP	Thermo Scientific A-11122	IF 1:1000	Human
γH2AX	Cell Signaling 2577	FC 1:200	Mouse
		IF 1:200	
	Upstate (Millipore) 05-636	WB 1:1000	Human
		IF 1:100	
HPRT	Abcam ab10479	WB 1:10000	Mouse
KAP1	Abcam ab10483	WB 1:10000	Mouse/Human
KAP1 pS824	Bethyl A300-767A	WB 1:1000	Human
KU70	Abcam ab3114	WB 1:500/1:2000	Mouse/Human
KU86	Santa Cruz sc-1484	WB 1:100	Mouse/Human
KU86	Thermo Scientific Ab-2	IF 1:100	Human
LIG4	Abcam ab80514	WB 1:1000	Mouse
	Abcam ab193353	WB 1:1000	Human
MRE11	Abcam ab33125	WB 1:10000	Mouse
P53	Cell Signaling 2524	WB 1:1000	Human
P53 pS15	Cell Signaling 9284	WB 1:1000	Human
PAXX	Abcam ab126353	WB 1:200	Mouse
RAD51	Santa Cruz sc-8349	IF 1:100	Human
RPA2	Abcam ab2175	FC 1:500	Mouse

RPA2	Abcam ab2175	IF 1:200	Mouse
TUBULIN	Sigma-Aldrich T9026	WB 1:5000	Mouse
XLF	Bethyl A300-730A	WB 1:5000	Mouse
XRCC4	Santa Cruz sc-8285	WB 1:200	Mouse
	Abcam ab145	WB 1:5000	Human

Supplementary Table 1. Antibodies used in this work.

WB: western blotting; FC: flow cytometry; IF: immunofluorescence; N/A: not applicable, as Cas9 is a bacterial protein. The ABRAXAS1 antibody was a kind gift of Bin Wang[26]. The CTIP antibody was a kind gift of Richard J. Baer[27].

**Targeted gene**	**Species**	**DNA sequence (5’-3’)**	**Plasmid name**
Abraxas	Mouse	CTTCACCTTTCATTTCACCG**AGG**	pAiO-WT-Abraxas1-1
		CTGGGAATGTCTGATCAAC**TGG**	pAiO-WT-Abraxas1-2
Babam1/Merit40	Mouse	CATCTGTCTGGATCTTTCGG**AGG**	pAiO-WT-Babam1-1
		ACGCCGTCTCCAGGTCATAC**AGG**	pAiO-WT-Babam1-2
Brcc3/Brcc36	Mouse	TACAGGTTGGCTGAACTAAC**AGG**	pAiO-WT-Brcc3-1
		TCTAGTTGAATGATGACATA**AGG**	pAiO-WT-Brcc3-2
Bre/Brcc45	Mouse	AACTGCACATCCCGTACGCT**GGG**	pAiO-WT-Bre-1
		AATGAGACATCCTCCTCCG**GGG**	pAiO-WT-Bre-2
ATM	Human	GTTGGTTACATACTTGGACT**TGG**	pAiO-WT-ATM
Hprt	Mouse	GTGCTTTAATGTAATCCAGC**AGG**	pU6-Hprt-1
Lig4	Mouse	ACAAAGATGGCGCGCTGTAC**CGG**	pAiO-WT-Lig4-1
		TACATCGAAACTAAGCTTGA**TGG**	pAiO-WT-Lig4-2
LIG4	Human	GAAAGAGAGAGAATGGCCTA**TGG**	pAiO-NK-LIG4
		GGAAGAATTAGTCTCATTGC**TGG**	
Msh6	Mouse	GGTATCACGAAACTTTAGAA**TGG**	pU6-Msh6
Xlf	Mouse	AGCAGCATTGCTTCGGATGA**AGG**	pAiO-WT-Xlf-1
		CCCCACTCTCCTGGTAGGCC**TGG**	pAiO-WT-Xlf-2
pac	*S.a.*	CGCTCAACTCGGCCATGCGC**GGG**	pU6-Puro-1
		GCAACAGATGGAAGGCCTCC**TGG**	pU6-Puro-2
Paxx	Mouse	TGACGGACGCCGCCGAGCTC**TGG**	pAiO-WT-Paxx-1
		AGCCCGATTTGGCCTAAGTG**GGG**	pAiO-WT-Paxx-2
Prkdc	Mouse	AGCCACTGGCTCCTCGGGAT**TGG**	pAiO-WT-Prkdc-83
Tp53bp1	Mouse	GCCTTACCCAGTTCCCGAGG**AGG**	pAiO-WT-53bp1-1
		CATTCCTGACACTCTACGAT**TGG**	pAiO-WT-53bp1-2
Xrcc4	Mouse	TTACTGACGGCCATTCAGCC**TGG**	pU6-Xrcc4-1
		TTGCAGAAAATAAGGTACGT**TGG**	pU6-Xrcc4-2
Xrcc5	Mouse	GACGCCCTGATTGTGTGCA**TGG**	pAiO-WT-Xrcc5

Supplementary Table 2. Sequences of DNA templates used for sgRNA production.

Protospacer Adjacent Motif (PAM) sequences are highlighted in bold. pAiO-WT (Cas9 wild-type nuclease; pAiO-Cas9) and pAiO-NK (Cas9 nickase; pAiO-Cas9^{D10A}) have been described previously[13]. *S.a.*: *Streptomyces alboniger*.

Targeted gene	Species	Targeted exon	DNA sequence oligonucleotide pairs (5’-3’)
Abraxas	Mouse	2	CTCGGCATGCAAACTACCAA
		7	ATACTGGGTGAGATGGCAGG
Babam1/Merit40	Mouse	2	CCACCCCTGAGCCCTTTT
		4	GACCCAGCACCAAAGTCTTG
Brcc3/Brcc36	Mouse	2	TGTGTGTGAACATATGGCCT
		5	ACATTGGCAGTTCTGGTCATG
Bre/Brcc45	Mouse	2	TTGACTCCTGGACCCAACTG
		6	AGTCTTACCTGTGTGTCCTCA
Lig4	Mouse	2	CATCTGTCCCGAACGCATTG
			GAGCGTGTGGAGAAGGACAT
LIG4	Human	2	GAGGGCATCTTTTCCATCTCTAGGTAA
			GATTCTTGGAGAAAATTTCATGATGC
Xlf	Mouse	4*	CCCACCCATTCCTCTGAGAA
			GTTGGTGGCTAATTGAGGGC
Paxx	Mouse	2	AAACTGCCCTACCCTGGAAA
			GTGGAGTCTAGGGATGTGGG
		3	CAGTTACTGCCTGCTGTTGG
			GCGGGGTTAGTGACAATTCC
Tp53bp1	*Mouse*	19	AGCTCAAGGTGGGGCATTC
			AGCCATCACCTTAGCTTCCT
pac	*S.a.*		pU6-Puro-1

Supplementary Table 3. Sequences of DNA oligonucleotides used for diagnostic PCR of CRISPR-Cas9 gene-editing events.

In *Xlf* the (*) symbol denotes possible targeted exon due to incorrect annotation of the *Xlf* gene in the GRCm38.p4 genome assembly.

Gene	Sequence
Ctip (#1)	GGACAAAACUAAAAGAGUA
Ctip (#2)	ACUUAAUGGUCAAGAAGAA
LIG4	AGGAAGUAUUCUCAGGAAUUA
Luciferase (siControl)	CGUACGCGGAAUACUUCGA

Ctip (#1) and *Ctip (#2)* represent two siRNA against the mouse *Ctip* gene.

Supplementary Table 4. siRNA sequences used for protein depletion.

Supplementary References

1. Improving bioscience research reporting: the ARRIVE guidelines for reporting animal research. (2010).

2. Zamboni, W. C. *et al.* Studies of the efficacy and pharmacology of irinotecan against human colon tumor xenograft models. *Clin Cancer Res* **4,** 743–753 (1998).

3. Guidelines for the welfare and use of animals in cancer research. **102,** 1555–1577 (2010).

4. Barlow, C. *et al.* Atm-deficient mice: a paradigm of ataxia telangiectasia. *Cell* **86,** 159–171 (1996).

5. Shultz, L. D. *et al.* Human lymphoid and myeloid cell development in NOD/LtSz-scid IL2R gamma null mice engrafted with mobilized human hemopoietic stem cells. *J Immunol* **174,** 6477–6489 (2005).

6. Elling, U. *et al.* Forward and reverse genetics through derivation of haploid mouse embryonic stem cells. *Cell Stem Cell* **9,** 563–574 (2011).

7. Leeb, M. & Wutz, A. Derivation of haploid embryonic stem cells from mouse embryos. *Nature* **479,** 131–134 (2011).

8. Koike-Yusa, H., Li, Y., Tan, E.-P., Del Castillo Velasco-Herrera, M. & Yusa, K. Genome-wide recessive genetic screening in mammalian cells with a lentiviral CRISPR-guide RNA library. *Nature Biotechnology* **32,** 267–273 (2014).

9. Yusa, K. *et al.* Targeted gene correction of α1-antitrypsin deficiency in induced pluripotent stem cells. *Nature* **478,** 391–394 (2011).

10. Swann, P. F. *et al.* Role of postreplicative DNA mismatch repair in the cytotoxic action of thioguanine. *Science* **273,** 1109–1111 (1996).

11. Li, W. *et al.* MAGeCK enables robust identification of essential genes from

genome-scale CRISPR/Cas9 knockout screens. *Genome Biol.* **15,** 554 (2014).

12. Liberzon, A. *et al.* The Molecular Signatures Database (MSigDB) hallmark gene set collection. *Cell Syst* **1,** 417–425 (2015).
13. Chiang, T.-W. W., le Sage, C., Larrieu, D., Demir, M. & Jackson, S. P. CRISPR-Cas9(D10A) nickase-based genotypic and phenotypic screening to enhance genome editing. *Sci Rep* **6,** 24356 (2016).
14. Balmus, G. *et al.* Cross-species chromosome painting among camel, cattle, pig and human: further insights into the putative Cetartiodactyla ancestral karyotype. *Chromosome Res.* **15,** 499–514 (2007).
15. Bayani, J. & Squire, J. A. *Sister Chromatid Exchange*. *Current Protocols in Cell Biology* **65,** 22.7.1–22.7.4 (John Wiley & Sons, Inc., 2005).
16. Elling, U. *et al.* A reversible haploid mouse embryonic stem cell biobank resource for functional genomics. *Nature* **550,** 114 (2017).
17. Nishi, R. *et al.* Systematic characterization of deubiquitylating enzymes for roles in maintaining genome integrity. *Nature Cell Biology* **16,** 1016–1026 (2014).
18. Borisova, M. E., Wagner, S. A. & Beli, P. Mass Spectrometry-Based Proteomics for Quantifying DNA Damage-Induced Phosphorylation. *Methods Mol. Biol.* **1599,** 215–227 (2017).
19. Ong, S.-E. *et al.* Stable isotope labeling by amino acids in cell culture, SILAC, as a simple and accurate approach to expression proteomics. *Mol. Cell Proteomics* **1,** 376–386 (2002).
20. Rappsilber, J., Mann, M. & Ishihama, Y. Protocol for micro-purification, enrichment, pre-fractionation and storage of peptides for proteomics using StageTips. *Nat Protoc* **2,** 1896–1906 (2007).
21. Michalski, A. *et al.* Mass spectrometry-based proteomics using Q Exactive, a

high-performance benchtop quadrupole Orbitrap mass spectrometer. *Mol. Cell Proteomics* **10,** M111.011015 (2011).

22. Olsen, J. V. *et al.* Higher-energy C-trap dissociation for peptide modification analysis. *Nat Methods* **4,** 709–712 (2007).
23. Cox, J. & Mann, M. MaxQuant enables high peptide identification rates, individualized p.p.b.-range mass accuracies and proteome-wide protein quantification. *Nature Biotechnology* **26,** 1367–1372 (2008).
24. Elias, J. E. & Gygi, S. P. Target-decoy search strategy for increased confidence in large-scale protein identifications by mass spectrometry. *Nat Methods* **4,** 207–214 (2007).
25. Franceschini, A. *et al.* STRING v9.1: protein-protein interaction networks, with increased coverage and integration. *Nucleic Acids Res.* **41,** D808–15 (2013).
26. Wu, Q. *et al.* Structure of BRCA1-BRCT/Abraxas Complex Reveals Phosphorylation-Dependent BRCT Dimerization at DNA Damage Sites. *Mol. Cell* **61,** 434–448 (2016).
27. Yu, X. & Baer, R. Nuclear localization and cell cycle-specific expression of CtIP, a protein that associates with the BRCA1 tumor suppressor. *J. Biol. Chem.* **275,** 18541–18549 (2000).
28. Kim, Y. M., Lee, J.-Y., Xia, L., Mulvihill, J. J. & Li, S. Trisomy 8: a common finding in mouse embryonic stem (ES) cell lines. *Mol Cytogenet* **6,** 3 (2013).
29. Morey, R. & Laurent, L. C. Getting off the ground state: X chromosome inactivation knocks down barriers to differentiation. *Cell Stem Cell* **14,** 131–132 (2014).

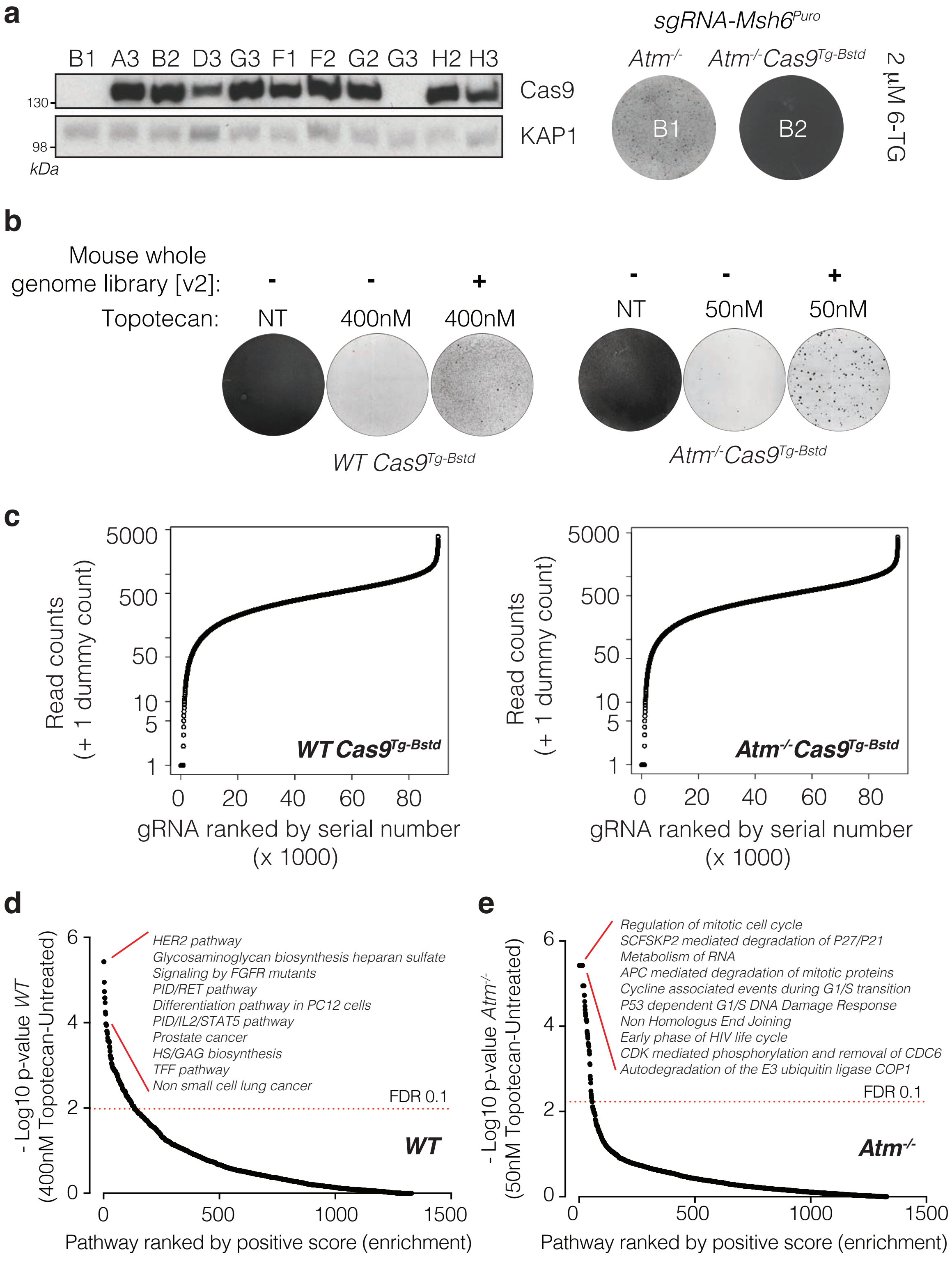
a
B1 A3 B2 D3 G3 F1 F2 G2 G3 H2 H3
130
98
kDa
Cas9
KAP1
sgRNA-Msh6Puro
Atm-/-
Atm-/-Cas9Tg-Bstd
B1
B2
2 μM 6-TG
b
Mouse whole genome library [v2]:
Topotecan:
NT
400nM
400nM
50nM
50nM
WT Cas9Tg-Bstd
Atm-/-Cas9Tg-Bstd
c
Read counts (+ 1 dummy count)
5000
500
50
10
5
1
WT Cas9Tg-Bstd
Atm-/-Cas9Tg-Bstd
0 20 40 60 80
gRNA ranked by serial number (x 1000)
d
- Log10 p-value WT (400nM Topotecan-Untreated)
HER2 pathway
Glycosaminoglycan biosynthesis heparan sulfate
Signaling by FGFR mutants
PID/RET pathway
Differentiation pathway in PC12 cells
PID/IL2/STAT5 pathway
Prostate cancer
HS/GAG biosynthesis
TFF pathway
Non small cell lung cancer
FDR 0.1
WT
0 500 1000 1500
Pathway ranked by positive score (enrichment)
e
- Log10 p-value Atm-/- (50nM Topotecan-Untreated)
Regulation of mitotic cell cycle
SCFSKP2 mediated degradation of P27/P21
Metabolism of RNA
APC mediated degradation of mitotic proteins
Cycline associated events during G1/S transition
P53 dependent G1/S DNA Damage Response
Non Homologus End Joining
Early phase of HIV life cycle
CDK mediated phosphorylation and removal of CDC6
Autodegradation of the E3 ubiquitin ligase COP1
FDR 0.1
Atm-/-
Pathway ranked by positive score (enrichment)

Supplementary Figure 1. Set-up for CRISPR-Cas9 screening. (a) Left panel: western blot showing Cas9 expression in various *Atm*$^{-/-}$ mutant clones. Right panel: efficiency of Cas9 gene-editing activity is shown by the ability of sgRNAs targeting the mismatch repair gene *Msh6* to generate resistance to the toxic antimetabolite 6-thioguanine (6-TG) in cells expressing Cas9 nuclease (clone B2) compared to cells that do not express Cas9 (clone B1). Similar analysis was done for the *Atm*$^{+/+}$ isogenic cells (not shown). **(b)** Representative scans of 15-cm screen plates of the representative genotypes that were either infected or not with the mouse whole genome library and subjected to chronic topotecan treatment at the indicated doses for 6 days, recovered for 4 days, fixed with methanol and stained using crystal violet. **(c)** Graphs showing the read counts of the library in the representation samples depicting good coverage. **(d and e)** Classification of the most enriched CRISPR-targeted pathways in wild-type **(d)** and *Atm*$^{-/-}$ **(e)** mESCs resistant to topotecan. Dotted red lines represent false-discovery-rate (FDR) thresholds. Represented are the names of the top hits with highest enrichment scores. All data were analyzed using MAGeCK (see Materials and methods) and are available in **Supplementary Data file 1-4**.

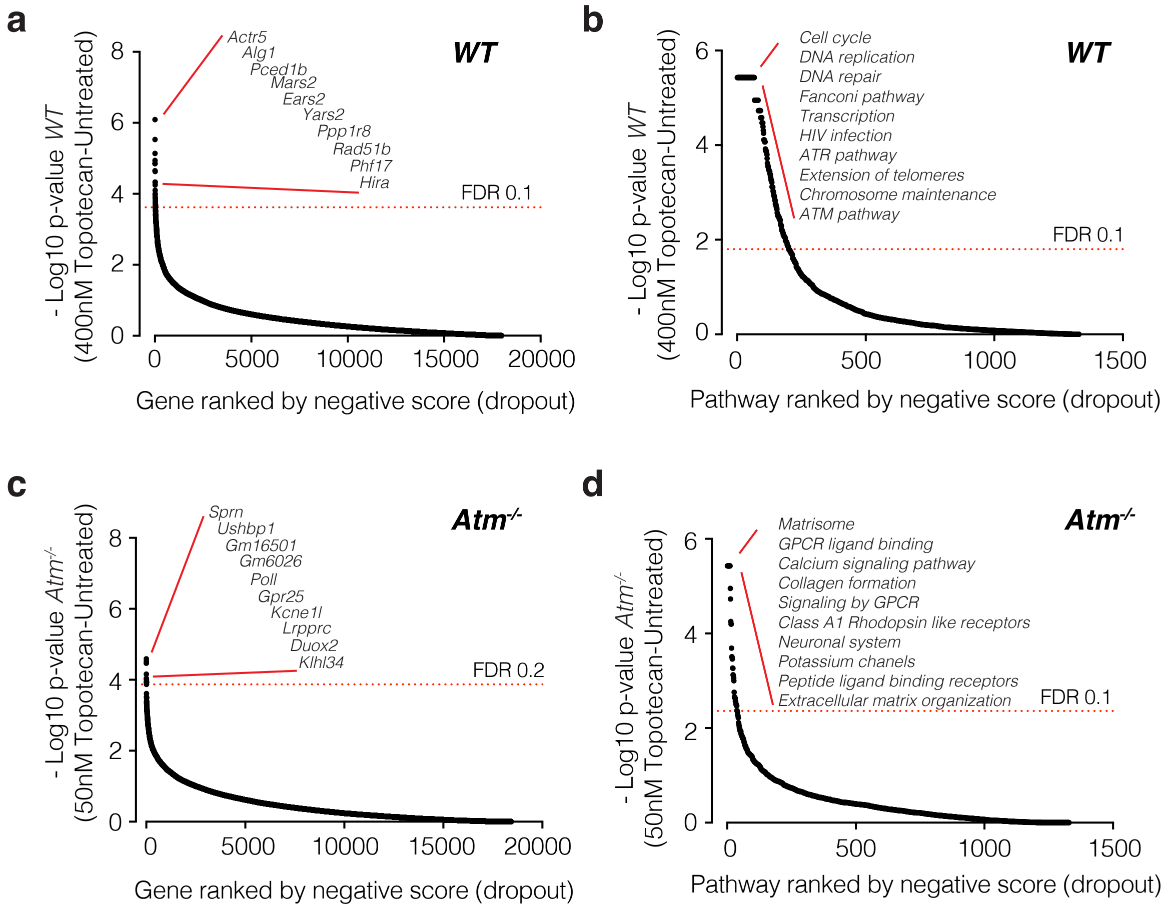

Supplementary Figure 2. Dropout analysis in response to topotecan treatment. Classification of depleted CRISPR-targeted genes **(a** and **c)** and pathways **(b** and **d)** in wild-type (**a** and **b**) or *Atm*$^{-/-}$ (**c** and **d**) mESCs treated with topotecan. Dotted red lines represent false discovery rate (FDR) thresholds. Represented are the names of the top hits. All data were analyzed using MAGeCK (see Materials and Methods) and are available in **Supplementary Data file 1-4**.

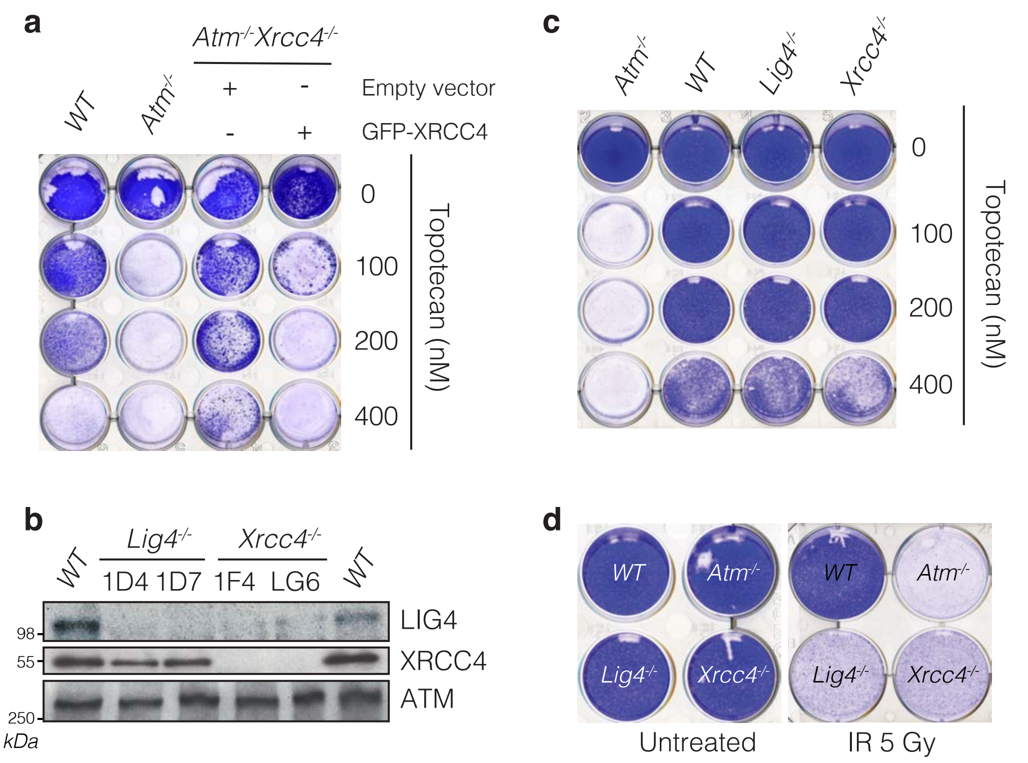

Supplementary Figure 3. Generation of *Lig4* and *Xrcc4* mutants in the *Atm*$^{+/+}$ background. (a) Re-expression of exogenous XRCC4 restores topotecan hypersensitivity in *Atm*$^{-/-}$ *Xrcc4*$^{-/-}$ cells. **(b)** Representative western blot image showing absence of LIG4 or XRCC4 in mutant cells generated by CRISPR-Cas9 gene editing. For detailed protocol see associated Materials and Methods text. **(c)** LIG4 or XRCC4 inactivation in an ATM-proficient background does not result in increased topotecan resistance. (**d**) *Lig4* and *Xrcc4* mutant cells in an *Atm* wild-type background display IR hypersensitivity. ATM-deficient cells were used as positive control.

For all panels containing western blot images, the raw images used can be visualized in **Supplementary Figure S10**; images are representative of three individual experiments; individual clone names are represented below the genotypes.

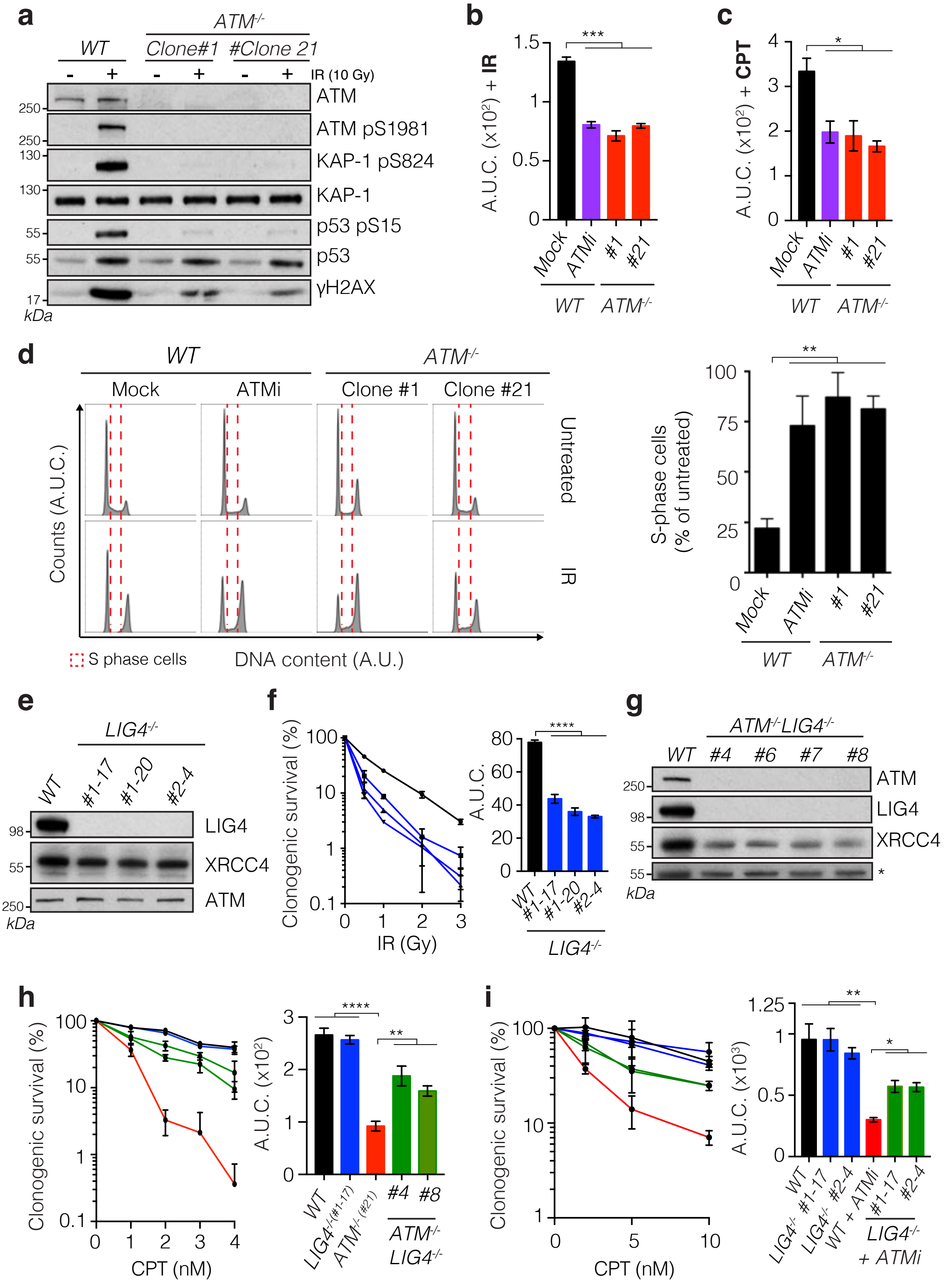
a
ATM-/-
WT
Clone#1
#Clone 21
IR (10 Gy)
ATM
ATM pS1981
KAP-1 pS824
KAP-1
p53 pS15
p53
γH2AX
kDa
b
A.U.C. (x10²) + IR
***
Mock
ATMi
#1
#21
WT
ATM-/-
c
A.U.C. (x10²) + CPT
*
d
WT
ATM-/-
Mock
ATMi
Clone #1
Clone #21
Untreated
IR
Counts (A.U.C.)
S phase cells
DNA content (A.U.)
S-phase cells (% of untreated)
**
e
LIG4-/-
WT
#1-17
#1-20
#2-4
LIG4
XRCC4
ATM
kDa
f
Clonogenic survival (%)
IR (Gy)
A.U.C.
****
g
ATM-/-LIG4-/-
WT
#4
#6
#7
#8
ATM
LIG4
XRCC4
*
kDa
h
Clonogenic survival (%)
CPT (nM)
A.U.C. (x10²)
****
**
LIG4-/-(#1-17)
ATM-/-(#21)
ATM-/-
LIG4-/-
i
A.U.C. (x10³)
LIG4-/- #1-17
LIG4-/- #2-4
WT + ATMi
LIG4-/-
+ ATMi

Supplementary Figure 4. Generation of *ATM*$^{-/-}$*, LIG4*$^{-/-}$ and *ATM*$^{-/-}$*LIG4*$^{-/-}$ human RPE-1 cells. (a) Representative image of western blot showing absence of ATM protein, and defects in ATM-mediated cell signaling in two different ATM-deficient clones. Cells were not treated of treated with 10 Gy IR, and samples were collected 1 h afterwards. **(b** and **c)** A.U.C. quantification based on clonogenic survival assays showing that ATM-deficient cells are hypersensitive to IR **(b)** and camptothecin **(c)**. A.U.C.: area under the curve **(d)** ATM-deficient cells do not activate the G1/S DNA damage checkpoint. Right panel: flow-cytometry based quantification of cells in S phase 8 h after 10 Gy IR. ATM-deficient cells or wild-type cells treated with ATM inhibitor do not arrest in G1 after DNA damage and enter S phase. Left panels: representative cell-cycle profiles used for quantifications described above. S-phase cells are shown delimited by red lines. A.U.: arbitrary units. **(e)** Representative image of western blot showing absence of LIG4 in three different LIG4-deficient clones. **(f)** Clonogenic survival assays showing that LIG4-deficient cells are hypersensitive to IR. **(g)** Representative image of western blot showing absence of ATM and LIG4 proteins in four different *ATM*$^{-/-}$ *LIG4*$^{-/-}$ clones. **(h)** Clonogenic survival assays showing that *ATM*$^{-/-}$ *LIG4*$^{-/-}$ cells are resistant to camptothecin. **(i)** Clonogenic survival assays showing that ATM-inhibited *LIG4*$^{-/-}$ cells are resistant to camptothecin.

All panels containing clonogenic survival assays (left) and A.U.C. (Area Under Curve) (right) were generated by using GraphPad Prism 7. Bars represent mean ± s.e.m.; ****p<0.0001; ***p<0.001; **p<0.01; *p<0.05; ns= not significant (p>0.05); two-tailed Student's t test following F test to confirm equal variance**;** n=3 individual experiments/n≥3 technical repeats for each genotype in each replicate.

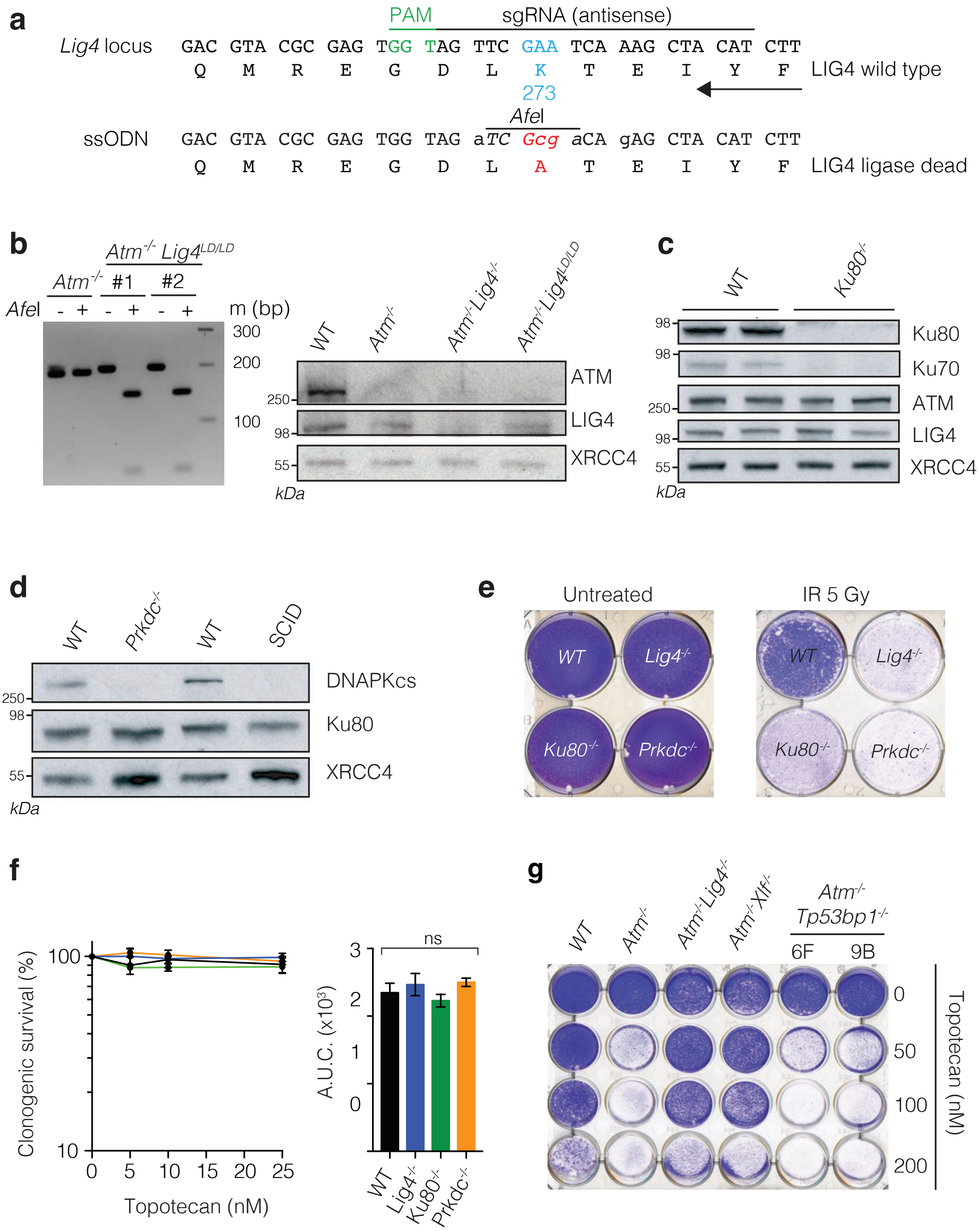
a
PAM
sgRNA (antisense)
Lig4 locus GAC GTA CGC GAG TGG TAG TTC GAA TCA AAG CTA CAT CTT
Q M R E G D L K T E I Y F LIG4 wild type
273
AfeI
ssODN GAC GTA CGC GAG TGG TAG aTC Gcg aCA gAG CTA CAT CTT
Q M R E G D L A T E I Y F LIG4 ligase dead
b
Atm-/- Lig4LD/LD
Atm-/- #1 #2
AfeI - + - + - +
m (bp)
300
200
100
WT
Atm-/-
Atm-/-Lig4-/-
Atm-/-Lig4LD/LD
250
98
55
kDa
ATM
LIG4
XRCC4
c
WT
Ku80-/-
98
98
250
98
55
kDa
Ku80
Ku70
ATM
LIG4
XRCC4
d
WT
Prkdc-/-
WT
SCID
250
98
55
kDa
DNAPKcs
Ku80
XRCC4
e
Untreated
IR 5 Gy
WT
Lig4-/-
Ku80-/-
Prkdc-/-
f
Clonogenic survival (%)
100
10
0 5 10 15 20 25
Topotecan (nM)
ns
3
2
1
0
A.U.C. (x10³)
WT
Lig4-/-
Ku80-/-
Prkdc-/-
g
WT
Atm-/-
Atm-/-Lig4-/-
Atm-/-Xlf-/-
Atm-/-
Tp53bp1-/-
6F
9B
0
50
100
200
Topotecan (nM)

Supplementary Figure 5. Generation of *Atm*$^{-/-}$*Lig4*$^{LD/LD}$ cells, and characterization of *Ku80* and *DNAPKcs* knock-out cells. (a) Top: DNA and amino acid sequence of the region surrounding the LIG4 catalytic site K273 (highlighted in blue) in the *Lig4* wild-type locus. Nucleotides comprising the sgRNA annealing site are marked with a line, and the Protospacer Adjacent Motif (PAM) is highlighted in green. Direction of the *Lig4* open reading frame is marked with an arrow. Bottom: DNA and amino acid sequence of the region containing mutations (all in lowercase) designed to inactivate LIG4 ligase activity (K273A, highlighted in red) and the annealing site for the sgRNA in the single-stranded oligonucleotide (ssODN) used as repair template. The new recognition site for the restriction enzyme *Afe*I is marked with a line and shown in italics. **(b)** Left: DNA restriction analysis of the *Lig4* locus in *Atm* mutant cells and in two gene-edited clones. Right: representative image of western blot analysis showing presence of the LIG4 protein in *Atm*$^{-/-}$*Lig4*$^{LD/LD}$ cells. Protein extracts from *Atm*$^{-/-}$ and LIG4-deficient cells are shown for comparative purposes. LD: ligase-dead allele. (c,d) Representative image of western blot showing absence of KU70/KU80 protein products and DNA-PKcs (PRKDC) protein products in corresponding mutant cells generated by CRISPR-Cas9 gene editing. Protein extracts from mESCs derived from a DNA-PKcs mutant SCID mouse were used as positive control. **(e)** *Ku80*$^{-/-}$ and *Prkdc*$^{-/-}$ mutant cells in an *Atm*$^{+/+}$ background exhibit IR hypersensitivity. LIG4-deficient cells were used as positive control. **(f)** Clonogenic survival assays showing no differences in sensitivity to topotecan between wild-type, *Ku80*$^{-/-}$, *Lig4*$^{-/-}$ and *Prkdc*$^{-/-}$ cells. The clonogenic survival assay (left) and A.U.C. (Area Under Curve) (right) were generated by using GraphPad Prism 7. Bars represent mean ± s.e.m.; ns= not significant ($p>0.05$); two-tailed Student's t test following F test to confirm equal variance; n=3 individual experiments/n≥3 technical repeats for each genotype in each replicate. **(g)**

Crystal violet cell viability assay showing that *Atm* mutant mESCs are hypersensitive to topotecan. Inactivation of *Lig4* or *Xlf*, but not of *Tp53bp1*, rescues the topotecan hypersensitivity of *Atm*$^{-/-}$ cells. Images are representative of three individual experiments; n≥3 for each genotype).

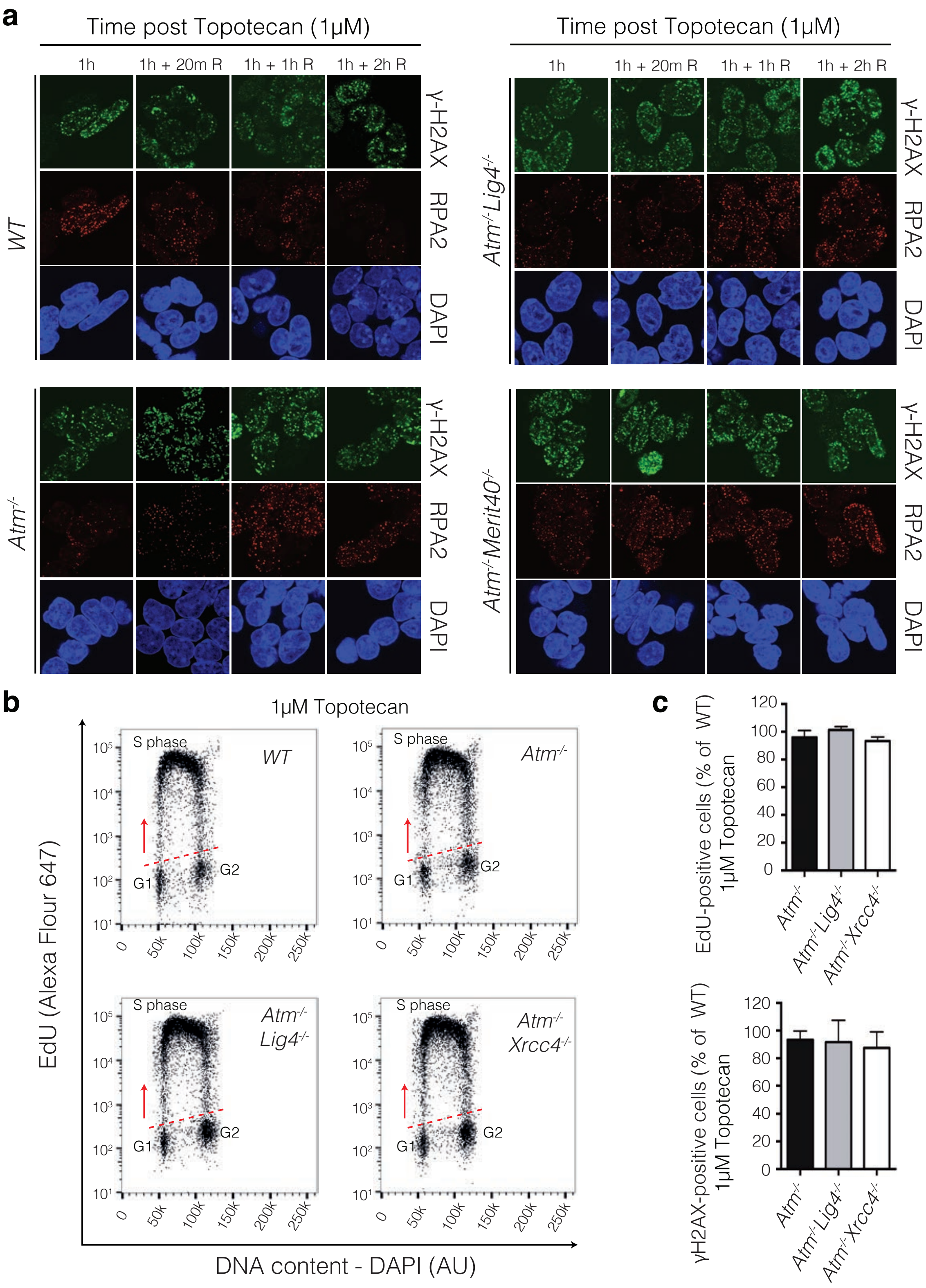
a
Time post Topotecan (1μM)
1h
1h + 20m R
1h + 1h R
1h + 2h R
γ-H2AX
RPA2
DAPI
WT
Atm-/-
Atm-/-Lig4-/-
Atm-/-Merit40-/-
b
1μM Topotecan
S phase
WT
Atm-/-
Atm-/- Lig4-/-
Atm-/- Xrcc4-/-
G1
G2
EdU (Alexa Flour 647)
DNA content - DAPI (AU)
c
EdU-positive cells (% of WT) 1μM Topotecan
γH2AX-positive cells (% of WT) 1μM Topotecan
Atm-/-
Atm-/-Lig4-/-
Atm-/-Xrcc4-/-

Supplementary Figure 6. Representative images of RPA2 kinetics in *WT*, *Atm*$^{-/-}$, *Atm*$^{-/-}$*Lig4*$^{-/-}$ and *Atm*$^{-/-}$*Merit40*$^{-/-}$ cells. (a) Delayed RPA2 foci formation in ATM-null cells. Cells were treated for 1 hour with 1 μM topotecan and recovered for the indicated times after treatment. RPA2 foci intensity was used as a readout for DNA-end resection in γH2AX positive cells and quantified (see **Figure 6c**). Images are representative of three individual experiments; n≥50 nuclei for each genotype were quantified. **(b-c)** Absence of LIG4 or XRCC4 does not impair DNA replication or DNA-damage accumulation in ATM-deficient cells upon topotecan treatment. Representative flow cytometry charts of cells incorporating EdU in the indicated genetic backgrounds upon 1μM topotecan treatment for 1 hour **(b)** and quantification **(c)** of cells incorporating EdU in each of the mutant backgrounds (left) and of cells positive for the DNA-damage marker γ-H2AX (right) in samples treated with the TOP1 poison topotecan. Incorporation of the nucleotide analogue EdU measures replication rates in the *y* axis, and distinguishes S phase cells from cells in either of the gap phases (G1 and G2). DNA content is measured by DAPI staining in the *x* axis. A.U.: arbitrary units. Error bars represent the standard error of the mean (SEM) from three independent experiments (n≥3 for each genotype).

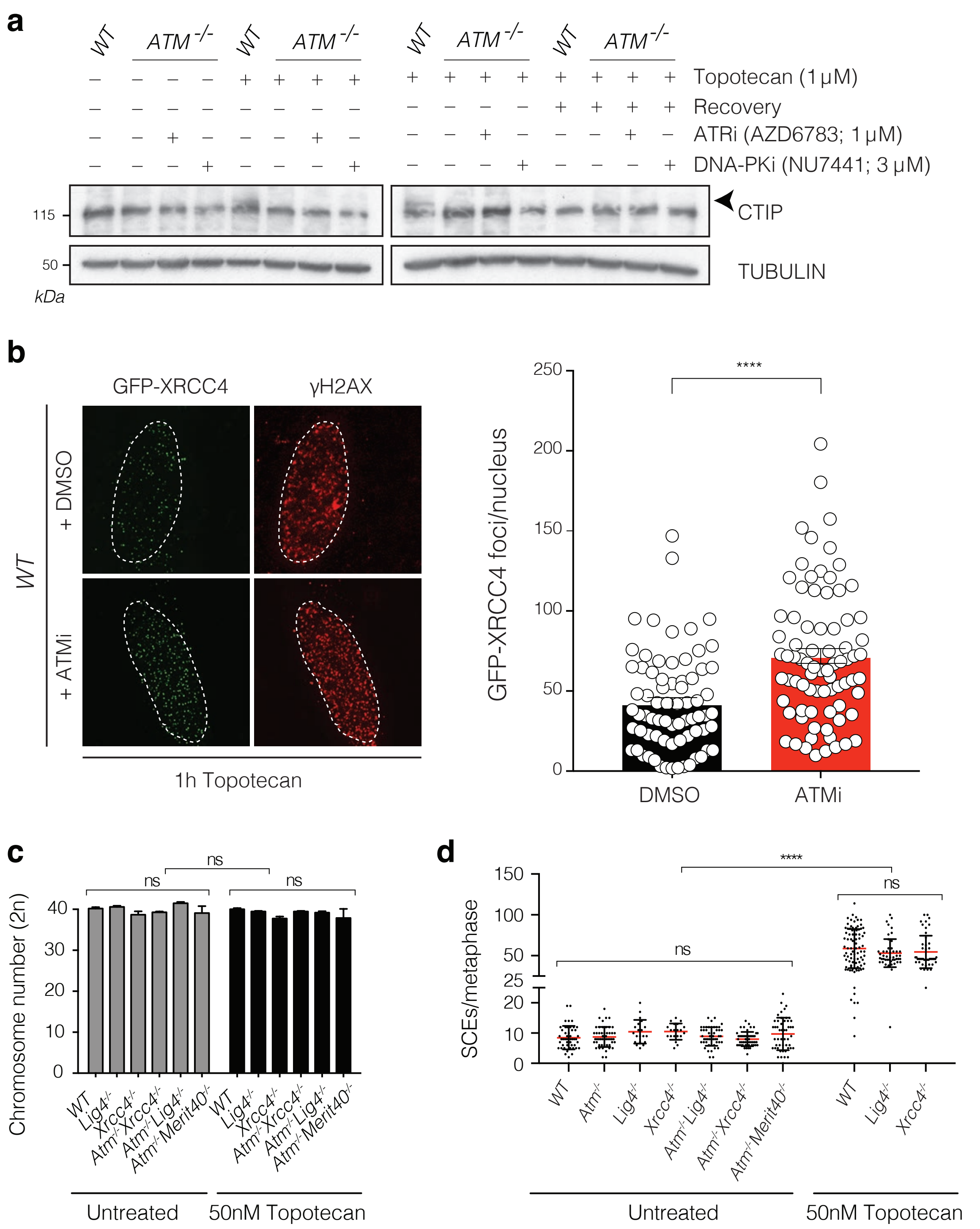

Supplementary Figure 7. Analysis of replication proficiency and chromosome number in mutant mESCs. (a) Representative western blot analysis of lysates from cells from the representative genotypes upon 1 hour 1 μM topotecan treatment (top panel) or 1 hour after drug removal (bottom panel), treated or not with ATR (ATRi;

AZD6738) or DNA-PKcs (DNA-PKi; NU7441) inhibitors. The arrow indicates the mobility shift of the CTIP protein caused by DNA-damage induction. **(b)** Representative images and quantifications of GFP-XRCC4 foci in γH2AX-positive nuclei in U2OS cells treated with topotecan. Cells were pre-incubated with DMSO or ATM inhibitor. Dashed outline indicates periphery of nuclei. Bars represent mean ± s.e.m.; ****p<0.0001; *p<0.05; two-tailed Student's t test following F test to confirm equal variance; df=4. **(c)** Chromosome number analysis of cells of the representative genotypes either untreated or treated with 50 nM topotecan shows no overt effect on the karyotype of the mouse cells (*Mus musculus*; 2n = 40). **(d)** Quantification of sister chromatid exchanges (SCEs) in cells of the representative genotypes cells treated or not with topotecan (for representative images and additional data of the comparison between the topotecan treated samples see **Figure 8a, b**). Scatter dot plots showing the number of SCEs across the representative genotypes were generated by using GraphPad Prism 7. n=3 individual experiments measuring n≥15 metaphases/genotype (for the untreated) and n≥33 for the topotecan treated in each experiment. Bars represent mean ± s.d.; ****p>0.0001; ns= not significant (p>0.05); two-tailed Student's t test following F test to confirm equal variance.

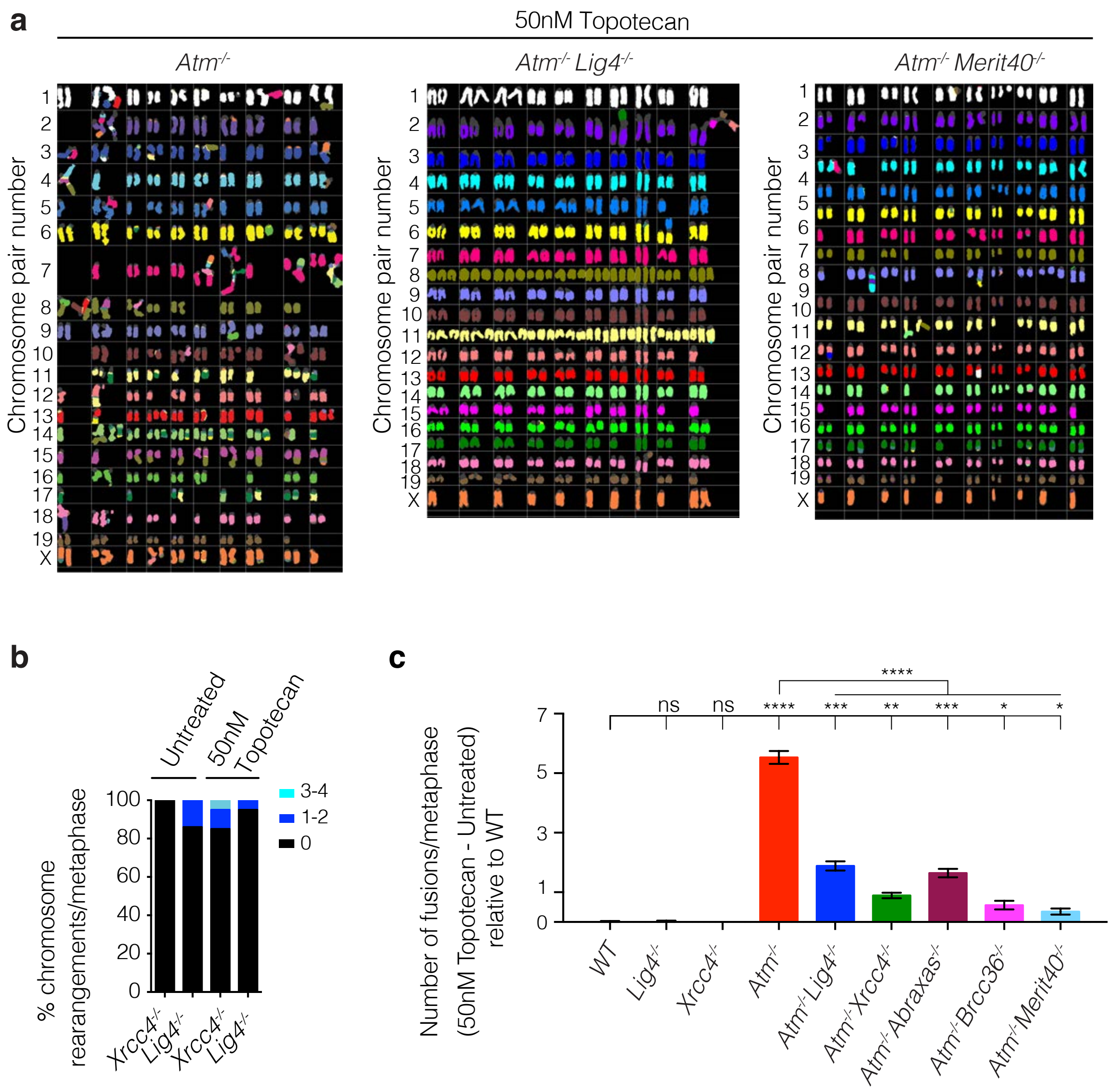

Supplementary Figure 8. Karyotype analysis of chromosome spreads mutant mESCs. (a) Representative Fluorescence In-Situ Hybridization (FISH) karyotypes of ten random and independent metaphase spreads from either *Atm*$^{-/-}$, *Atm*$^{-/-}$*Lig4*$^{-/-}$ or *Atm*$^{-/-}$*Merit40*$^{-/-}$ mESCs treated with 50nM topotecan reveal increased chromosomal instability in ATM-deficient cells that was suppressed by loss of LIG4 or MERIT40. Similar observations to those with *Atm*$^{-/-}$*Lig4*$^{-/-}$ mESCs were made with *Atm*$^{-/-}$*Xrcc4*$^{-/-}$, *Atm*$^{-/-}$*Abraxas*$^{-/-}$ and *Atm*$^{-/-}$*Brcc36*$^{-/-}$ mESCs (karyotype data not shown) and quantified in **Figure 8d**. Note that the *Atm*$^{-/-}$*Lig4*$^{-/-}$ represented clone had an inherent trisomy 8

and 11 that are common in mESCs[28]; mESCs are reported to randomly loose one X chromosome[29]. **(b)** LIG4/XRCC4-deficient cells do not show increased chromosomal rearrangements. Quantifications of radial chromosomes in cells treated with topotecan. **(c)** ATM-deficient cells show increased number of chromosome fusions. Quantification of chromosome fusions in cells treated with 50nM topotecan as compared to the baseline in untreated cells (all relative to WT). Graph was generated using GraphPad Prism 7. Bars represent mean ± s.e.m.; ****p<0.0001; ***p<0.001; **p<0.01; *p<0.05; ns= not significant (p>0.05); two-tailed Student's t test following F test to confirm equal variance**;** n=3 individual experiments measuring n≥20 metaphases/genotype in each experiment were karyotyped.

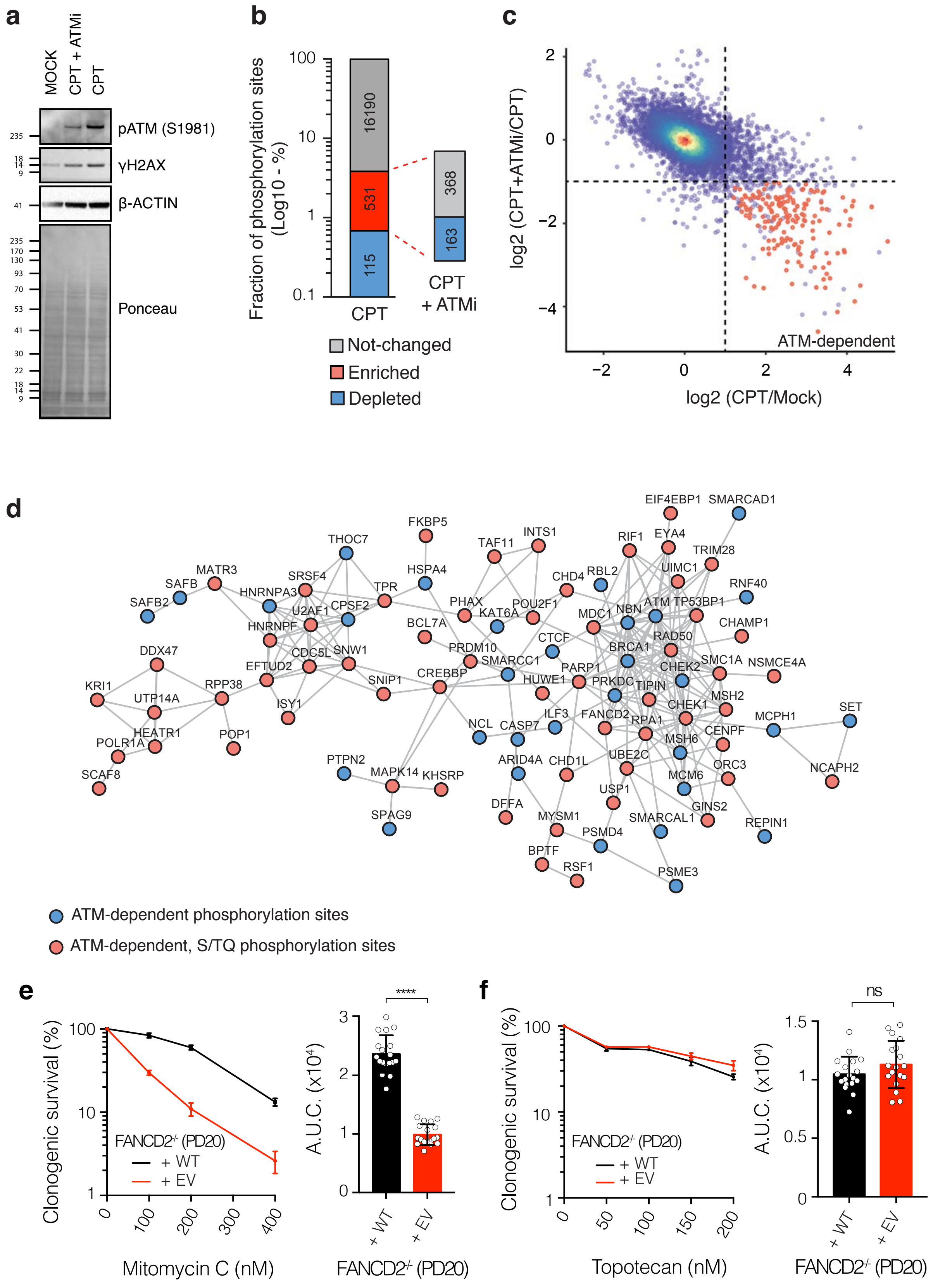
a
MOCK
CPT + ATMi
CPT
pATM (S1981)
γH2AX
β-ACTIN
Ponceau
b
Fraction of phosphorylation sites (Log10 - %)
16190
531
115
368
163
CPT
CPT + ATMi
Not-changed
Enriched
Depleted
c
log2 (CPT+ATMi/CPT)
log2 (CPT/Mock)
ATM-dependent
d
ATM-dependent phosphorylation sites
ATM-dependent, S/TQ phosphorylation sites
e
Clonogenic survival (%)
FANCD2-/- (PD20)
+ WT
+ EV
Mitomycin C (nM)
A.U.C. (x10^4)
****
FANCD2-/- (PD20)
f
Clonogenic survival (%)
FANCD2-/- (PD20)
+ WT
+ EV
Topotecan (nM)
A.U.C. (x10^4)
ns
FANCD2-/- (PD20)

Supplementary Figure 9. Phosphoproteomics reveals ATM-dependent phosphorylation sites after camptothecin treatment and clonogenic survival of FANCD2-deficient cells. (a) Triple SILAC-labeled HEK293T cells were mock-treated or treated with camptothecin (CPT) for 2 hours with or without 1 hour pre-incubation with ATM inhibitor (ATMi; KU-55933; 10 µM). **(b)** Phosphorylated peptides were enriched and analyzed by LC-MS/MS. The bar graph shows the number of enriched, not changed or depleted sites after camptothecin treatment as well as the number of not changed or depleted sites after inhibition of ATM identified from two independent biological experiments. Phosphorylation of 531 sites increases at least two-fold in abundance after camptothecin treatment and 163 of these sites are dependent on ATM. **(c)** The scatter plot shows the logarithmised SILAC ratios of quantified phosphorylation sites from two independent biological replicates. The color-coding indicates the density. Phosphorylation sites color-coded in red are induced after camptothecin treatment in an ATM-dependent manner. Dotted lines indicate the SILAC ratio of 2 (CPT/Mock) and (CPT+ATMi/CPT). **(d)** Functional interaction network shows proteins that are phosphorylated in an ATM-dependent manner after camptothecin treatment. Proteins phosphorylated on an S/TQ motif are indicated. Proteins with ATM-dependent phosphorylation sites that do not form a network are not shown. **(e)** Survival assays showing no differences in sensitivity to topotecan between FANCD2-deficient PD20 cells complemented with either wild-type (WT) FANCD2 or empty vector (EV). Error bars represent standard error of the mean (s.e.m.) from three independent experiments. A.U.C.: area under the curve. (**f**) Survival assays showing hypersensitivity to mitomycin C of FANCD2-deficient PD20 cells compared to PD20 cells complemented with wild-type FANCD2. Error bars represent standard error of the mean (s.e.m.) from three independent experiments. A.U.C.: area under the curve.

Bars represent mean ± s.e.m.; ****$p<0.0001$; ns= not significant ($p>0.05$); two-tailed Student's t test following F test to confirm equal variance**;** $n=3$ individual experiments/$n\geq3$ technical repeats for each genotype in each replicate.

Balmus et al.Supplementary Figure 10a

Related to Fig. 1a

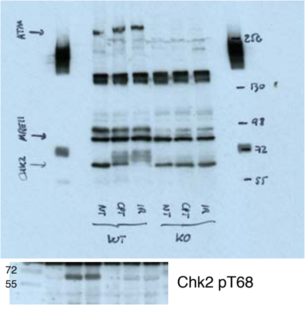

Related to Fig. 2a

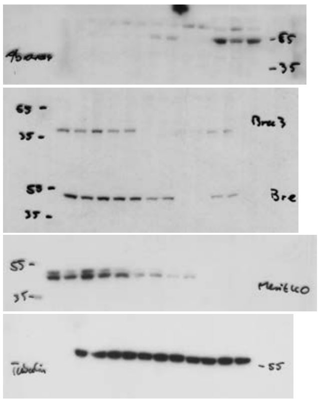

Related to Fig. 2c

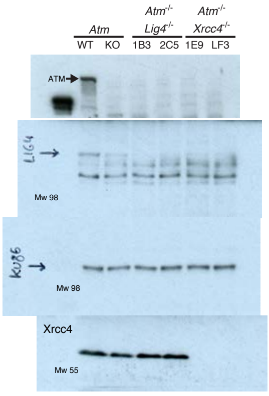

Related to Fig. 4b

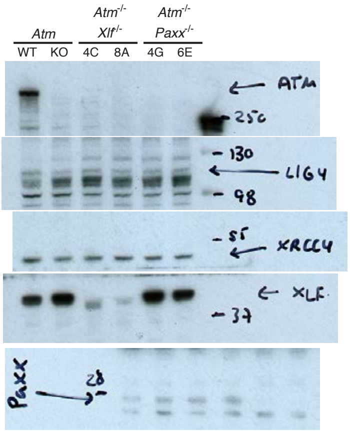

Related to Fig. 5c

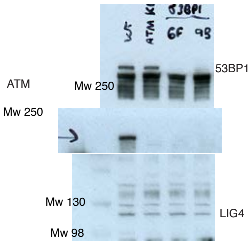

Related to Fig. 7a

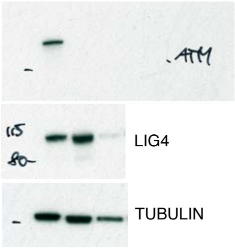

Related to Fig. 7c

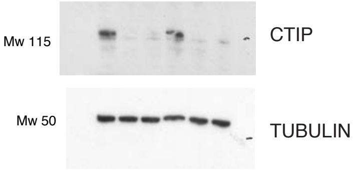

Related to Fig. 7e

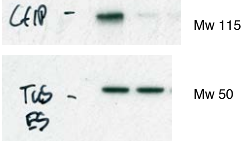

Balmus et al. Supplementary Figure 10b

Related to Fig. S1a

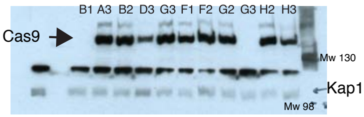

Related to Fig. S3b

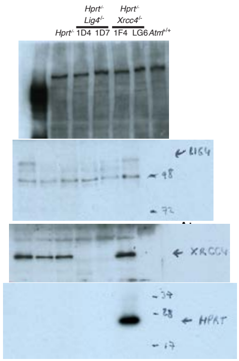

Related to Fig. S4a

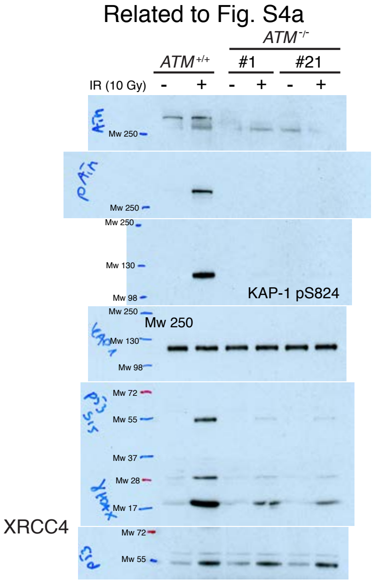

Related to Fig. S4e

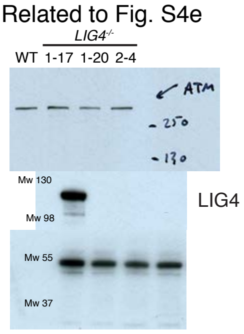

Related to Fig. S5b

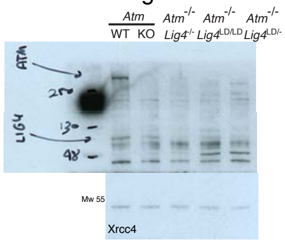

Related to Fig. S4g

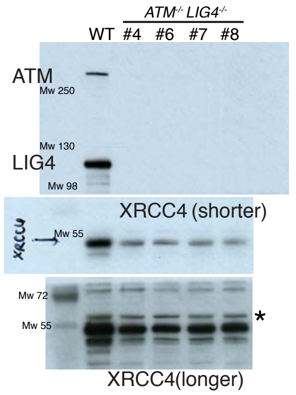

Related to Fig. S5c

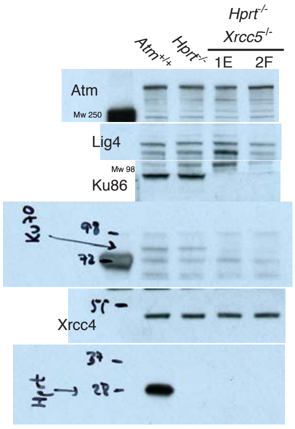

Related to Fig. S5d

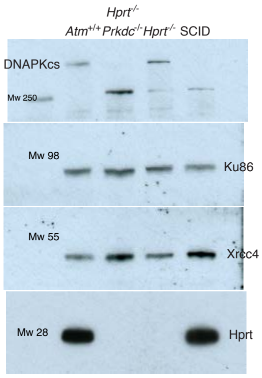

Related to Fig. S7b

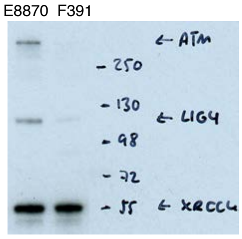

Related to Fig. S8a

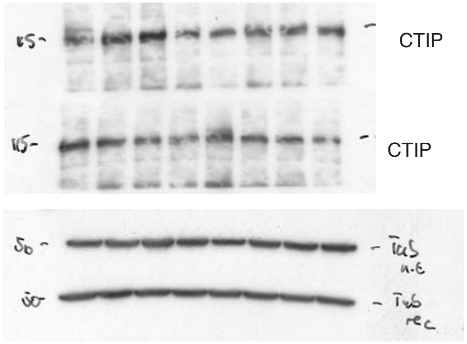

Balmus et al. Supplementary Figure 10c

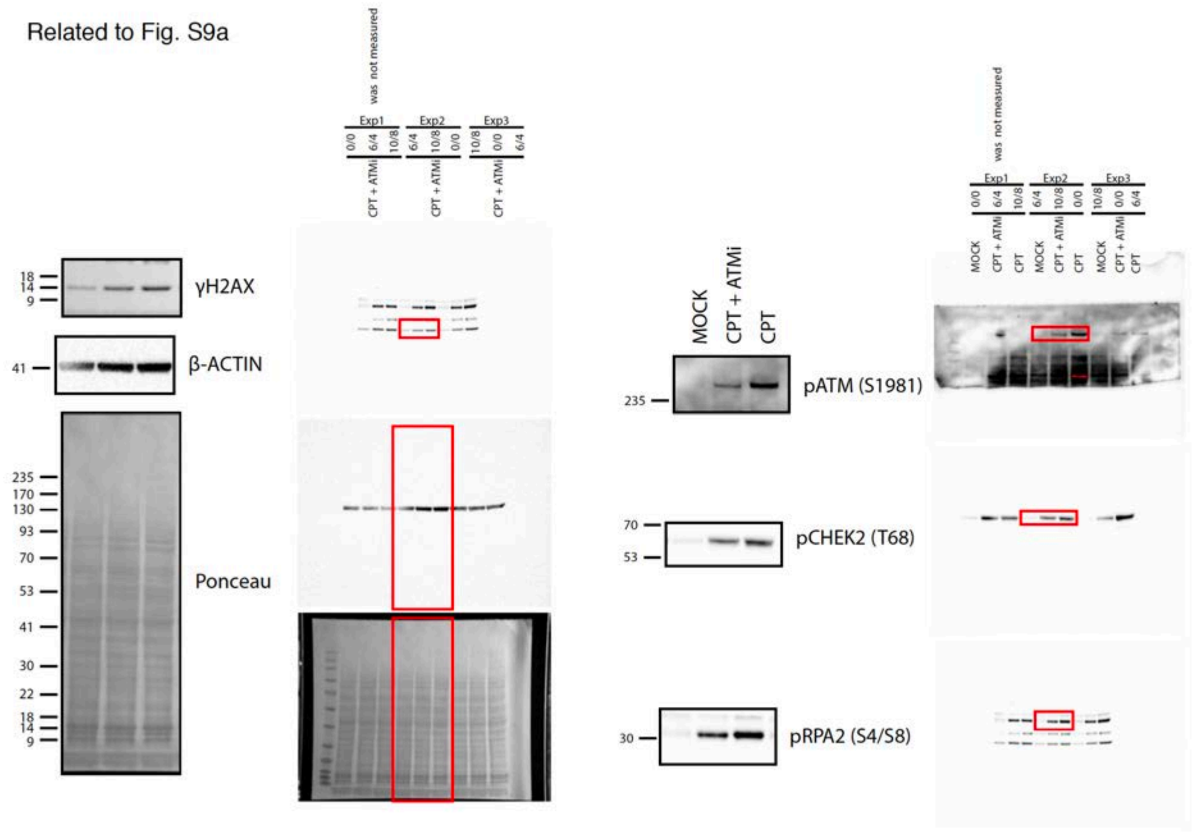

Supplementary Figure 10 (a, b and c). Image scans for all the western blot data used in the figures of the present manuscript.
