# Supplementary material for: ATM orchestrates the DNA-damage response to counter toxic non-homologous end-joining at broken replication forks
Source: Nat Commun. 2019 Jan 8;10:87. doi: 10.1038/s41467-018-07729-2 (PMC6325118; doi:10.1038/s41467-018-07729-2)
Supplement: Supplementary file 2 — Description of Additional Supplementary Files [file 41467_2018_7729_MOESM2_ESM.pdf]

Description of Additional Supplementary Files

File Name: Supplementary Data 1
Description: Gene analysis of CRISPR/Cas9 screen in Atm+/+ cells

File Name: Supplementary Data 2
Description: Gene analysis of CRISPR/Cas9 screen in Atm-/- cells

File Name: Supplementary Data 3
Description: Pathway analysis of CRISPR/Cas9 screen in Atm+/+ cells

File Name: Supplementary Data 4
Description: Pathway analysis of CRISPR/Cas9 screen in Atm-/- cells

File Name: Supplementary Data 5
Description: Gene analysis of CRISPR/Cas9 screen in HT-29 cells

File Name: Supplementary Data 6
Description: Phospho proteome in response to CPT treatment
