# Supplementary material for: ATM orchestrates the DNA-damage response to counter toxic non-homologous end-joining at broken replication forks
Source: Nat Commun. 2019 Jan 8;10:87. doi: 10.1038/s41467-018-07729-2 (PMC6325118; doi:10.1038/s41467-018-07729-2)
Supplement: Supplementary file 9 — Reporting Summary [file 41467_2018_7729_MOESM9_ESM.pdf]

nature research

Corresponding author(s): Jackson S.P.

Reporting Summary

Nature Research wishes to improve the reproducibility of the work that we publish. This form provides structure for consistency and transparency in reporting. For further information on Nature Research policies, see Authors & Referees and the Editorial Policy Checklist.

Statistical parameters

When statistical analyses are reported, confirm that the following items are present in the relevant location (e.g. figure legend, table legend, main text, or Methods section).

n/a	Confirmed	
☐	☒	The exact sample size (*n*) for each experimental group/condition, given as a discrete number and unit of measurement
☐	☒	An indication of whether measurements were taken from distinct samples or whether the same sample was measured repeatedly
☐	☒	The statistical test(s) used AND whether they are one- or two-sided<br>*Only common tests should be described solely by name; describe more complex techniques in the Methods section.*
☐	☒	A description of all covariates tested
☐	☒	A description of any assumptions or corrections, such as tests of normality and adjustment for multiple comparisons
☐	☒	A full description of the statistics including central tendency (e.g. means) or other basic estimates (e.g. regression coefficient) AND variation (e.g. standard deviation) or associated estimates of uncertainty (e.g. confidence intervals)
☐	☒	For null hypothesis testing, the test statistic (e.g. *F*, *t*, *r*) with confidence intervals, effect sizes, degrees of freedom and *P* value noted<br>*Give P values as exact values whenever suitable.*
☒	☐	For Bayesian analysis, information on the choice of priors and Markov chain Monte Carlo settings
☒	☐	For hierarchical and complex designs, identification of the appropriate level for tests and full reporting of outcomes
☒	☐	Estimates of effect sizes (e.g. Cohen's *d*, Pearson's *r*), indicating how they were calculated
☐	☒	Clearly defined error bars<br>*State explicitly what error bars represent (e.g. SD, SE, CI)*

Our web collection on statistics for biologists may be useful.

Software and code

Policy information about availability of computer code

Data collection	*Provide a description of all commercial, open source and custom code used to collect the data in this study, specifying the version used OR state that no software was used.*
Data analysis	for the CRISPR-Cas9 screen Mageck ; for statistical analyses GraphPad Prism 7; Microsoft Excel (Microsoft Inc, USA), Adobe Photoshop and Illustrator (Adobe Systems Inc, USA) were used to process data for publication

For manuscripts utilizing custom algorithms or software that are central to the research but not yet described in published literature, software must be made available to editors/reviewers upon request. We strongly encourage code deposition in a community repository (e.g. GitHub). See the Nature Research guidelines for submitting code & software for further information.

Data

Policy information about availability of data

All manuscripts must include a data availability statement. This statement should provide the following information, where applicable:

- Accession codes, unique identifiers, or web links for publicly available datasets
- A list of figures that have associated raw data
- A description of any restrictions on data availability

All relevant data are available from the authors upon request. The mass-spectrometry data can be downloaded from ProteomeXchange via the PRIDE database using PXD011108 project accession identifier.

Field-specific reporting

Please select the best fit for your research. If you are not sure, read the appropriate sections before making your selection.

☒ Life sciences ☐ Behavioural & social sciences ☐ Ecological, evolutionary & environmental sciences

For a reference copy of the document with all sections, see nature.com/authors/policies/ReportingSummary-flat.pdf

Life sciences study design

All studies must disclose on these points even when the disclosure is negative.

Sample size	*Describe how sample size was determined, detailing any statistical methods used to predetermine sample size OR if no sample-size calculation was performed, describe how sample sizes were chosen and provide a rationale for why these sample sizes are sufficient.*
Data exclusions	*Describe any data exclusions. If no data were excluded from the analyses, state so OR if data were excluded, describe the exclusions and the rationale behind them, indicating whether exclusion criteria were pre-established.*
Replication	All experiments were reliably reproduced as stated in the text, and detailed methods provided to aid in their replication by others. Where further methods/data are sought corresponding authors will oblige reasonable requests.
Randomization	*Describe how samples/organisms/participants were allocated into experimental groups. If allocation was not random, describe how covariates were controlled OR if this is not relevant to your study, explain why.*
Blinding	*Describe whether the investigators were blinded to group allocation during data collection and/or analysis. If blinding was not possible, describe why OR explain why blinding was not relevant to your study.*

Reporting for specific materials, systems and methods

Materials & experimental systems

n/a	Involved in the study
☐	☒ Unique biological materials
☐	☒ Antibodies
☐	☒ Eukaryotic cell lines
☐	☐ Palaeontology
☐	☒ Animals and other organisms
☐	☐ Human research participants

Methods

n/a	Involved in the study
☐	☐ ChIP-seq
☐	☒ Flow cytometry
☐	☐ MRI-based neuroimaging

Unique biological materials

Policy information about availability of materials

Obtaining unique materials	*Describe any restrictions on the availability of unique materials OR confirm that all unique materials used are readily available from the authors or from standard commercial sources (and specify these sources).*

Antibodies

Antibodies used	53BP1 Novus NB100-304 WB 1:5000

ABRAXAS1 Bing Wang lab WB 1:10000
ATM Serotec AHP392 WB 1:1000
ATM Epitomics 1549-1 WB 1:10000
ATM pS1981 Abcam ab81292 WB 1:5000
BABAM1/MERIT40 Cell Signaling 12711 WB 1:1000
BRCC3/BRCC36 Abcam ab207913 WB: 1:1000
BRE/BRCC45 Cell Signaling 12457S WB 1:1000
Cas9 Diagenode C15200203 WB 1:2000
CHK2 Millipore 05-649 WB 1:2000
CHK2 pT68 Cell Signaling 2661 WB 1:1000
CTIP Richard Baer lab WB 1:40
DNAPKcs Thermo Scientific MA5-13404 WB 1:200
GFP Thermo Scientific A-11122 IF 1:1000
gH2AX Cell Signaling 2577 FC 1:200
gH2AX Cell Signaling 2577 IF 1:200
Upstate (Millipore) 05-636 WB 1:1000
Upstate (Millipore) 05-636 IF 1:100
HPRT Abcam ab10479 WB 1:10000
KAP1 Abcam ab10483 WB 1:10000
KAP1 pS824 Bethyl A300-767A WB 1:1000
KU70 Abcam ab3114 WB 1:500/1:2000
KU86 Santa Cruz sc-1484 WB 1:100
KU86 Thermo Scientific Ab-2 IF 1:100
LIG4 Abcam ab80514 WB 1:1000
LIG4 Abcam ab193353 WB 1:1000
MRE11 Abcam ab33125 WB 1:10000
P53 Cell Signaling 2524 WB 1:1000
P53 pS15 Cell Signaling 9284 WB 1:1000
PAXX Abcam ab126353 WB 1:200
RAD51 Santa Cruz sc-8349 IF 1:100
RPA2 Abcam ab2175 FC 1:500
RPA2 Abcam ab2175 IF 1:200
TUBULIN Sigma-Aldrich T9026 WB 1:5000
XLF Bethyl A300-730A WB 1:5000
XRCC4 Santa Cruz sc-8285 WB 1:200
XRCC4 Abcam ab145 WB 1:5000

Validation

Each experiment had appropriate controls to validate the antibodies. Commercially available antibodies were validated by the supplier and by us using appropriate controls where needed; Supplementary Table 1. Non-commercial antibodies provided by colleagues were validated in their initial studies describing the corresponding antibody. Please also refer to the manufacturers' websites for further details.

Eukaryotic cell lines

Policy information about cell lines

Cell line source(s)

U2OS, RPE1, mESCs: S.P. Jackson lab (Gurdon Institute, Cambridge, UK)
HT29: (ECACC, Cat# 91072201)

Authentication

All cells were originally obtained from the ATCC cell repository if not otherwise stated, and we have authenticated cell lines used in our study by STR profiling,

Mycoplasma contamination

All cells are routinely tested to be mycoplasma free.

Commonly misidentified lines
(See ICLAC register)

No commonly misidentified cell lines were used.

Palaeontology

Specimen provenance

Provide provenance information for specimens and describe permits that were obtained for the work (including the name of the issuing authority, the date of issue, and any identifying information).

Specimen deposition

Indicate where the specimens have been deposited to permit free access by other researchers.

Dating methods

If new dates are provided, describe how they were obtained (e.g. collection, storage, sample pretreatment and measurement), where they were obtained (i.e. lab name), the calibration program and the protocol for quality assurance OR state that no new dates are provided.

☐ Tick this box to confirm that the raw and calibrated dates are available in the paper or in Supplementary Information.

Animals and other organisms

Policy information about studies involving animals; ARRIVE guidelines recommended for reporting animal research

Laboratory animals	Care and use of all mice used for this paper was carried out in accordance with UK Home Office regulations, UK Animals (Scientific Procedures) Act of 2013 under UK Home Office licenses which approved this work and is reviewed regularly by the WTSI Animal Welfare and Ethical Review Board and along the ARRIVE guidelines. Full description is provided in Supplementary Material and methods.
Wild animals	The study did not involve wild animals
Field-collected samples	The study did not involve samples collected from the field

Human research participants

Policy information about studies involving human research participants

Population characteristics	N/A
Recruitment	N/A

ChIP-seq

Data deposition

☐ Confirm that both raw and final processed data have been deposited in a public database such as GEO.

☐ Confirm that you have deposited or provided access to graph files (e.g. BED files) for the called peaks.

Data access links<br>*May remain private before publication.*	N/A
Files in database submission	N/A
Genome browser session<br>(e.g. UCSC)	N/A

Methodology

Replicates	N/A
Sequencing depth	N/A
Antibodies	N/A
Peak calling parameters	N/A
Data quality	N/A
Software	N/A

Flow Cytometry

Plots

Confirm that:

☒ The axis labels state the marker and fluorochrome used (e.g. CD4-FITC).

☒ The axis scales are clearly visible. Include numbers along axes only for bottom left plot of group (a 'group' is an analysis of identical markers).

☒ All plots are contour plots with outliers or pseudocolor plots.

☐ A numerical value for number of cells or percentage (with statistics) is provided.

Methodology

Sample preparation	after collection cells were fixed in ice-cold 70% ethanol; additional details are provided in Material and methods
Instrument	Samples were sorted using a BD LSRFortessa cell analyser (BD Biosciences)
Software	The data was collected and analysed using FlowJo(BD Inc, USA).

Cell population abundance	*Describe the abundance of the relevant cell populations within post-sort fractions, providing details on the purity of the samples and how it was determined.*
Gating strategy	Doublets were distinguished from single cells by plotting FSC height vs FCS area.

☒ Tick this box to confirm that a figure exemplifying the gating strategy is provided in the Supplementary Information.

Magnetic resonance imaging

Experimental design

Design type	*Indicate task or resting state; event-related or block design.*
Design specifications	*Specify the number of blocks, trials or experimental units per session and/or subject, and specify the length of each trial or block (if trials are blocked) and interval between trials.*
Behavioral performance measures	*State number and/or type of variables recorded (e.g. correct button press, response time) and what statistics were used to establish that the subjects were performing the task as expected (e.g. mean, range, and/or standard deviation across subjects).*

Acquisition

Imaging type(s)	*Specify: functional, structural, diffusion, perfusion.*
Field strength	*Specify in Tesla*
Sequence & imaging parameters	*Specify the pulse sequence type (gradient echo, spin echo, etc.), imaging type (EPI, spiral, etc.), field of view, matrix size, slice thickness, orientation and TE/TR/flip angle.*
Area of acquisition	*State whether a whole brain scan was used OR define the area of acquisition, describing how the region was determined.*

Diffusion MRI ☐ Used ☐ Not used

Preprocessing

Preprocessing software	*Provide detail on software version and revision number and on specific parameters (model/functions, brain extraction, segmentation, smoothing kernel size, etc.).*
Normalization	*If data were normalized/standardized, describe the approach(es): specify linear or non-linear and define image types used for transformation OR indicate that data were not normalized and explain rationale for lack of normalization.*
Normalization template	*Describe the template used for normalization/transformation, specifying subject space or group standardized space (e.g. original Talairach, MNI305, ICBM152) OR indicate that the data were not normalized.*
Noise and artifact removal	*Describe your procedure(s) for artifact and structured noise removal, specifying motion parameters, tissue signals and physiological signals (heart rate, respiration).*
Volume censoring	*Define your software and/or method and criteria for volume censoring, and state the extent of such censoring.*

Statistical modeling & inference

Model type and settings	*Specify type (mass univariate, multivariate, RSA, predictive, etc.) and describe essential details of the model at the first and second levels (e.g. fixed, random or mixed effects; drift or auto-correlation).*
Effect(s) tested	*Define precise effect in terms of the task or stimulus conditions instead of psychological concepts and indicate whether ANOVA or factorial designs were used.*

Specify type of analysis: ☐ Whole brain ☐ ROI-based ☐ Both

Statistic type for inference (See Eklund et al. 2016)	*Specify voxel-wise or cluster-wise and report all relevant parameters for cluster-wise methods.*
Correction	*Describe the type of correction and how it is obtained for multiple comparisons (e.g. FWE, FDR, permutation or Monte Carlo).*

Models & analysis

n/a	Involved in the study
☒	☐ Functional and/or effective connectivity
☒	☐ Graph analysis
☒	☐ Multivariate modeling or predictive analysis
